# Sustainable Electrochemical-Magnetic Biosensor Fabricated from Recycled Materials for Label-Free Detection of SARS-CoV-2 in Human Saliva

**DOI:** 10.1021/acssensors.4c03175

**Published:** 2025-03-14

**Authors:** Caio Lenon Chaves Carvalho, Steffane Q. Nascimento, Thiago Bertaglia, Luana C. I. Faria, Erika R. Manuli, Geovana M. Pereira, Welter Cantanhêde da Silva, Carlos M. Costa, Josu Fernández Maestu, Senentxu Lanceros-Méndez, Osvaldo N. Oliveira, Ester C. Sabino, Frank N. Crespilho

**Affiliations:** a São Carlos Institute of Chemistry, University of São Paulo (USP), São Carlos 13560-970, Brazil; b Institute of Tropical Medicine, Faculty of Medicine, University of São Paulo, São Paulo, São Paulo 05403-000, Brazil; c LIM-46 HC-FMUSP − Laboratory of Medical Investigation, Clinical Hospital, Faculty of Medicine, University of São Paulo, São Paulo, São Paulo 01246903, Brazil; d Supramolecular Self-Assembly Laboratory, Department of Chemistry, Federal University of Piauí, Teresina, Piauí 64049-550, Brazil; e Physics Centre of Minho and Porto Universities (CF-UM-UP) and Laboratory of Physics for Materials and Emergent Technologies, LapMET, University of Minho, Braga 4710-057, Portugal; f Institute of Science and Innovation for Bio-Sustainability (IB-S), University of Minho, Braga 4710-053, Portugal; g BCMaterials, Basque Center for Materials, Applications and Nanostructures, UPV/EHU Science Park, Leioa 48940, Spain; h IKERBASQUE, Basque Foundation for Science, Bilbao 48009, Spain; i Sao Carlos Institute of Physics, University of São Paulo, São Carlos 13560-970, Brazil

**Keywords:** COVID-19 diagnostics, magnetic electrochemical biosensor, sustainable sensing, SARS-CoV-2 detection, recycled materials, point-of-care testing, additive manufacturing, healthcare sustainability

## Abstract

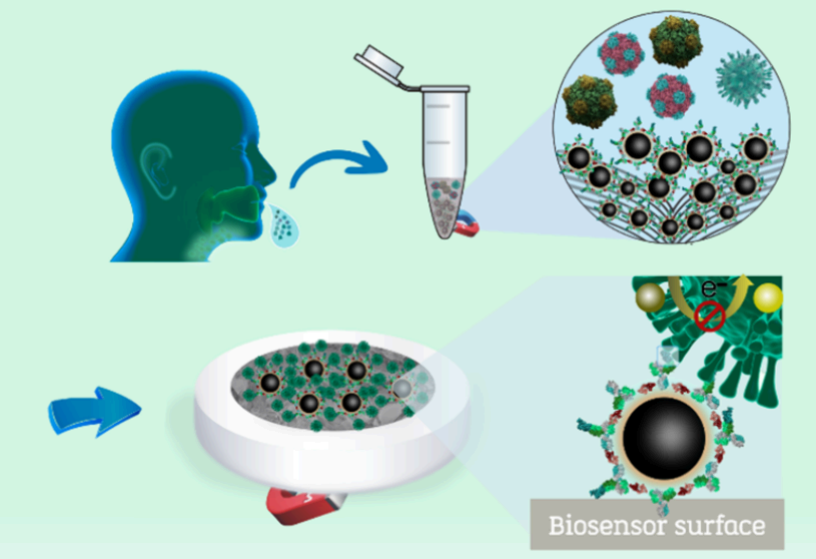

The COVID-19 pandemic has highlighted the critical need for scalable, rapid, and cost-effective diagnostic solutions, especially in resource-limited settings. In this study, we developed a sustainable magnetic electrochemical biosensor for the mass testing of SARS-CoV-2, emphasizing affordability, environmental impact reduction, and clinical applicability. By leveraging recycled materials from spent batteries and plastics, we achieved a circular economy-based fabrication process with a recyclability rate of 98.5%. The biosensor employs MnFe_2_O_4_ nanoparticles functionalized with anti-SARS-CoV-2 antibodies, integrated into a 3D-printed electrochemical device for decentralized testing. Advanced characterization confirmed the biosensor’s robust performance, including high sensitivity (LOD: 3.46 pg mL^–1^) and specificity, with results demonstrating a 95% correlation to RT-PCR gold standard testing. The cost of materials used per biosensor test is only USD 0.2, making it highly affordable and suitable for large-scale production using additive manufacturing. Key features include simple preparation, rapid response, and reusability, making it ideal for point-of-care diagnostics. Beyond COVID-19, this platform’s modularity allows for adaptation to other viral diseases, offering a versatile solution to global diagnostic challenges. This work highlights the potential of integrating electrochemical sensing with sustainable practices to address healthcare inequities and reduce environmental impact.

After the global COVID-19 pandemic, viral diseases like malaria, dengue fever, and HIV/AIDS continue to have a profound impact on global health, especially in low-income countries.^1^ These diseases affect millions annually and highlight the critical need for improved healthcare infrastructure and access to medical resources in vulnerable populations. After initially containing SARS-CoV-2 virus, many European and Asian countries experienced a resurgence of COVID-19 due to a large proportion of the population remaining susceptible after the first wave.^2^ Conversely, in Manaus, Brazil, 76% of the population had been infected. Despite this high attack rate, COVID-19 hospital admissions surged again in January 2021.^3^ Possible explanations include overestimated infection rates, waning immunity, new virus lineages with increased transmissibility or immune evasion, and behavioral factors.

Brazil has faced one of the fastest-growing SARS-CoV-2 epidemics globally, with the Amazon region, particularly Manaus, being the most affected. Manaus, a city with over 2 million people, saw its first SARS-CoV-2 case on March 13, 2020, followed by a rapid and severe outbreak peaking in early May with a significant increase in mortality.^4^ Despite relaxing nonpharmaceutical interventions (NPIs), new cases declined, suggesting the epidemic might have been contained due to reaching herd immunity or other factors like behavioral changes. The high attack rate in Manaus contrasts with the slower, more prolonged epidemic in São Paulo. To measure the true attack rate, antibody tests were conducted, revealing that test sensitivity varies based on disease severity and time since infection, with a decline in antibody levels over time.^4^

These findings highlight the complex dynamics of antibody responses and the need for ongoing surveillance to understand and manage the epidemic. Continued genomic and serological surveillance, alongside rapid data dissemination and vaccine efficacy studies, remain essential in navigating these resurgences. The prompt and precise detection of viral diseases is crucial for effective public health interventions. However, conventional diagnostic techniques often impose significant time and resource constraints, hindering outbreak control efforts. In response, we’ve initiated a collaborative initiative involving nine research centers and hospitals to formulate a swift, cost-effective strategy. This approach not only integrates cutting-edge procedures and protocols but also holds promise for technological advancement in mass testing. Magnetic electrochemical biosensors,^5,6^ employing magnetic nanoparticles for virus capture and detection in saliva samples, offer heightened sensitivity and align with sustainable principles. This method is very interesting because it does not depend on the amplification of the genetic material of the virus, like RT-PCR. Yet, the challenge lies in seamlessly integrating these biosensors into environmentally friendly platforms for widespread adoption, especially in resource-limited settings. Addressing this challenge effectively could pave the way for the development of reusable and recyclable devices tailored for mass testing in such contexts. In this work, we present the development of a label-free, universal platform for magnetic electrochemical biosensors for mass viral disease testing, leveraging recycled materials within a circular economy framework to promote sustainability and resource efficiency. By repurposing waste materials from batteries and plastics (Figure 1a), we reduce environmental impact and create a closed-loop system where materials are continuously recycled and reused. The use of material waste in electrochemical sensing devices has already been reported by other authors as an important paradigm for the introduction of circular economy in electrochemistry.^7,8^ This also means that electrochemistry, sustainability and recycling can share principles that promote the circular economy electrochemistry.

**Figure 1:**
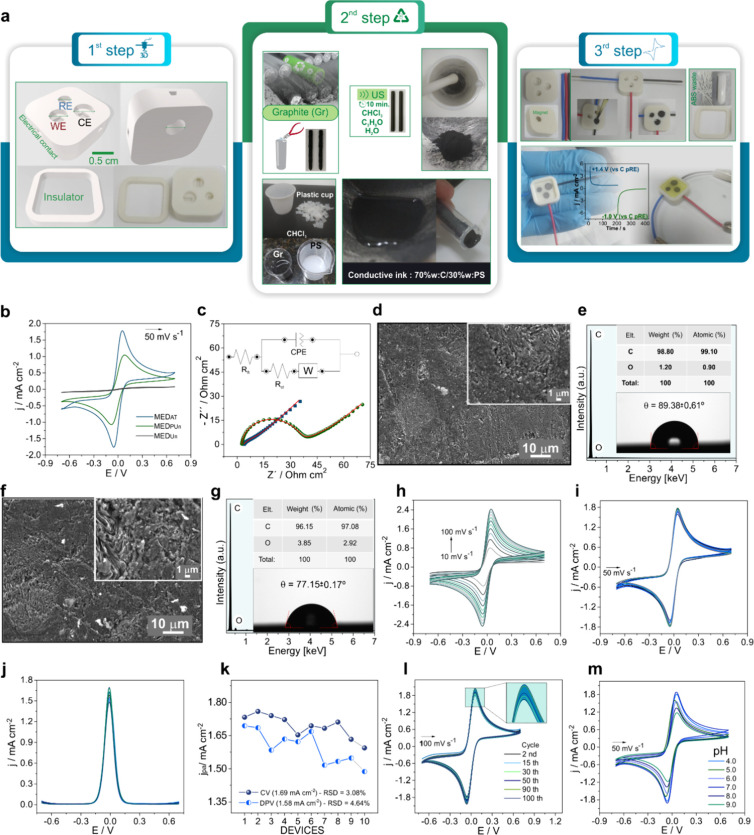
**Low-cost biosensors made from recycled materials (a)** Schematic representation for preparation of the 3D-printed magneto-integrated electrochemical device (MED) in 3-steps. (b) Cyclic voltammogram (CV) for MED untreated and unpolished (MEDUn), MED with surface polishing and untreated (MEDPUn), MED after electrochemical treatment (MEDAT) in 1 mol L^–1^ NaOH (+1.4 V/200 s and −1.0 V/200 s). (c) Electrochemical impedance spectroscopy (EIS) results from 100 kHz to 0.1 Hz at 0.01 V, inset: correspondence equivalent Nyquist circuit. (d) SEM image of MEDPUn_,_ (e) corresponding spectra EDX and measure of contact angle. (f) SEM image for MEDAT. (g) Spectra EDX and measure of contact angle for MEDAT. (h) CVs from 10 to 100 mV s^–1^ for MEDAT. **(i)** CVs of ten different devices independently prepared under alternate days (interday repeatability). **(j)** Study of interday repeatability (*n* = 10) for measurements of differential pulse voltammetry (DPV). **(k)** jpa0 versus number of electrodes. **(l)** Electrochemical behavior of 100 cycles at 100 mV s^–1^. **(m**) CVs for different pH (4.0, 5.0, 6.0, 7.0, 8.0, and 9.0). All electrochemical data were obtained in 5.0 mmol L^–1^ K_3_[Fe(CN)_6_]/K_4_[Fe(CN)_6_] (0.5 mol L^–1^ KCl). 95% confidence intervals for all error bars (*n* = 3).

Global expert panels and the scientific community have focused on environmentally sustainable strategies that aim to reduce pollution.^9,10^ In addition, the World Trade Organization (WTC) has discussed the global transition to a more circular economy.^9−12^ To this end, different targets need to be interconnected and scalable by 2050, including reducing consumption, choosing renewable materials, increasing recycling and maximizing waste removal to the environment.^9−11^ It is also important to mention that the development of more sustainable electrochemical sensors requires the discussion of the so-called “green credentials”.^13^ In the field of electrochemical sensing, researchers have used biodegradable and sustainable materials to manufacture environmentally safe devices. For example, electrochemical devices prepared by additive manufacturing (3D printing) based in the fabrication of Fused Filament Fabrication (FFF) from renewable and/or recycled sources are more sustainable strategies. Mainly due to the possibility of reducing environmental impact and low-cost of production of the devices.^14^

Our biosensors are integrated into point-of-care devices, enabling decentralized low-cost testing and reducing reliance on centralized laboratories. This is achieved through advanced manufacturing techniques like 3D printing, allowing rapid and cost-effective fabrication of customized biosensor platforms.^15^ The study on SARS-CoV-2 detection demonstrates the biosensors’ efficacy in clinical settings, with performance comparable to the gold standard RT-PCR.^16,17^ The fabrication of electrochemical device combines 3D printing technology and the preparation of a carbon-based conductive ink using recyclable materials (Figure 1a). The design of the biosensor includes advantageous features such as miniaturization, an integrated neodymium magnet, simple preparation, and high scalability for mass production. The manufacturing process can be easily developed by nonspecialists, which is crucial for point-of-care analyses. When designed to produce 1000 biosensors, the cost per device is only 0.106 USD (Supporting Information (SI), Figure S1 and Figure S2).

## Experimental Section

Details of the materials, reagents, and preparation of MnFe_2_O_4_ nanoparticles (MnFe_2_O_4_ NPs) are presented in the SI.

### Preparation of MnFe_2_O_4_ Bioconjugate

The bioconjugation of MnFe_2_O_4_ NPs with the anti-SARS-CoV-2 RBD antibody (S1-Ab) was performed using N-(3-(Dimethylamino)propyl)-N′-ethylcarbodiimide hydrochloride (EDC) and *N*-hydroxysuccinimide (NHS) coupling chemistry. Initially, 10 mg of MnFe_2_O_4_ NPs were dispersed in 6 mL of MES buffer (0.1 mol L^–1^, pH 5.0) and sonicated for 10 min. Following this, 300 μL of the nanoparticle dispersion, 20 mg of EDC, and 5.42 mg of NHS were mixed and stirred for 30 min at room temperature. With the aid of a neodymium magnet, the activated nanoparticles were washed five times with ultrapure water through their magnetic separation from the supernatant. The MnFe_2_O_4_ NPs were redispersed in 500 μL of phosphate-buffered saline (PBS, 1x, pH 7.4) containing 20 μg mL^–1^ of S1-Ab and incubated for 3 h at room temperature under moderate agitation. To block nonspecific binding sites, 500 μL of 1% (w/v) BSA solution was added and incubated for 30 min. The resulting MnFe_2_O_4_-EDC/NHS-S1-Ab/BSA bioconjugate was washed with PBS (1x, pH 7.4) and stored at 4 °C.

### Magneto-Integrated Electrochemical Device (MED)

The MED was fabricated using a three-step process, as illustrated in Figure 1a. *3D Printing of the Electrochemical Cell:* The miniaturized electrochemical cell was designed using FreeCAD0.21 software and printed using a Core A1 Dual 3D printer (GTMax3D, São Paulo-Brazil). The cell consists of a working electrode (WE), counter electrode (CE), pseudoreference electrode (pRE), and an insulator, with specific dimensions to accommodate a conductive carbon composite. The main printing parameters were configured in Simplify3Dv5.0 software, including 100% infill, nozzle temperature 230 °C, printing speed 37 mm s^–1^, and 0.2 mm layer thickness. The acrylonitrile butadiene styrene (ABS, Ø = 1.75 mm) premium was used as thermoplastic filament acquired from GTMax3D (Brazil). Printing each miniaturized cell takes approximately 15 min, which allows the manufacturing of multiple electrochemical devices per printing batch. The top surface of the newly printed electrochemical cell was polished with 400-grit sandpaper to eliminate deformations and make the surface flat. *Preparation of Conductive Graphite-Polystyrene Composite:* The composite was prepared using graphite (Gr) from spent zinc–carbon batteries and polystyrene (PS) from disposable cups. The graphite rods were cleaned, ground into fine powder, and mixed with PS dissolved in chloroform. The resulting suspension was stirred and sonicated to achieve a homogeneous and viscous mixture. It is important to highlight that all handling and volatilization of chloroform were carried under a fume hood exhaust to avoid harm to human health and the environment. *Assembly of the Magneto-Integrated Electrochemical Cell:* Flexible copper wires were inserted into the electrode holes for electrical contact, and aliquots of the Gr/PS mixture were placed in the electrode holes and dried. The electrodes were polished to achieve a smooth surface, and the insulator was glued to the MED using an ABS-acetone mixture. A neodymium magnet was placed at the bottom of the working electrode, and the surface was treated electrochemically for better performance. Thus, the surface of the WE underwent an electrochemical treatment procedure in alkaline medium. Initially, 250 μL of NaOH (1 mol L^–1^) was added inside the cell to cover the surface of the electrodes. Soon after, the electrochemical treatment was carried out by the sequential application of different potentials, + 1.4 V followed −1.0 V (vs pRE), both for 200 s. It is important to highlight that this is the first protocol reported in the literature to produce a conductive ink based on graphite from spent battery graphite and plastic cup (Supplementary Table 2).

Microstructural characterization of nanomaterials and electrodes in high resolution was investigated using a JEOL JSM-7200F field emission scanning electron microscope (FESEM, Japan). The measurements were performed with secondary electron in-lens (SEI) detector with acceleration voltage 5 to 15 kV and magnification up until x150,000. The microscope equipped with energy dispersive X-ray spectrometer (XFLASH 6–60 EDS, BRUKER) was also used to analyze the chemical composition of the samples.

The structure phase and crystallinity of the MnFe_2_O_4_-cys NPs were identified on a X-ray diffractometer Bruker D8 Advance ECO, operating at 25 mA, 40 kV, and applying Cu–Kα radiation of (λ = 1.5406 Å). Analysis was performed at a scan rate of 0.02°/min using the (2θ) from 10° to 90° under a continuous scan mode. The obtained XRD data for the MnFe_2_O_4_ NPs was compared with crystallographic data from No. 43462 ICSD (Inorganic Crystal Structure Database). The average size of nanocrystallites (D, in nm) was calculated using the Debye–Scherrer equation
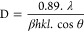
1where, β_hkl_ is half-height width of the maximum peak relative to the hkl plane (radians), θ is reflection angle (radians), 0.89 is constant related to the shape of the crystal (0.89, spherical geometry). Moreover, λ is the radiation wavelength (0.154056 nm), D is average size of nanocrystallites (nm). The lattice parameter a can be calculated from the Miller indices for (311) plane, using eq 2, where d is interplanar spacing. MnFe_2_O_4_ with cubic inverse spinel phase crystalline parameters: *a = b = c =* 8.45 Å.
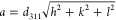
2

The FTIR spectra and micro-FTIR chemical mapping were obtained using a FTIR microscope (Hyperion 3000) coupled to a Bruker Vertex 70 V spectrometer (Bruker, Germany). All samples (MnFe_2_O_4_, MnFe_2_O_4_-EDC/NHS and MnFe_2_O_4_-EDC/NHS-S1-Ab) were prepared by *drop-casting* on a polycrystalline gold substrate. After optical focusing (36× magnification), the chemical images were collected using mercury telluride and cadmium detector (MCT) in an area of ∼78 × 78 μm^2^. All FTIR spectra were acquired in reflectance mode 64 accumulations with a spectral resolution of 8 cm^–1^ and a spectral window of 600–4000 cm^–1^. Only for MnFe_2_O_4_ NPs functionalized with l-cysteine were FTIR spectra collected in transmittance mode with scanning from 400 to 4000 cm^–1^ for the analysis of the (Fe–O and Mn–O) stretching vibrations. The sample was prepared by mixing MnFe_2_O_4_ NPs powder with KBr forming a pellet 1% w/w.

The spectroscopic characterization of the MEDPun and MEDAT electrodes was performed by Raman spectroscopy. The measurements were collected on Horiba LabRAM HR Evolution equipped with 1600 × 200 CCD detector (Symphony, Horiba), a 100× objective lens, operating with a 633 nm He–Ne red laser and filter 10%. All Raman spectra were obtained in the range of 150–3500 cm^–1^. The Lorentzian function was used for deconvolution of spectra referents to bands D, G and D′.

A MicroSense EZ7 vibrating sample magnetometer (VSM) was used to evaluate magnetic hysteresis loops at ambient temperature between −1.8 and 1.8 T. The superparamagnetic behavior of the nanoparticles is retained after functionalization and bioconjugation, essential for their use in magnetic separation and biosensing applications.

Electrochemical measurements were carried by cyclic voltammetry, EIS, and DPV techniques in the presence of 5.0 mmol L^-1^ K_3_[Fe(CN)_6_]/K_4_[Fe(CN)_6_] (0.1 mol L^-1^ PBS, pH 7.4). All experiments were performed with a μ-Autolab potentiostat/galvanostat (Metrohm Autolab, Utrecht, The Netherlands) Type III, coupled with FRA2 module, and for data acquisition was used Nova 2.1.5 software. The 3D-printed electrochemical minicell consisting of carbon ink as a working electrode, a carbon pseudo reference electrode (potential vs pseudo C) and a carbon counter electrode.

The electrochemical assays for detection of S1-RBD were conducted by DPV technique. The analysis conditions were performed in the range potential of −0.7 to 0.7 V, modulation amplitude of 25 mV, step potential of 5 mV, estimated duration of 139 s, and scan rate of 10 mV s^–1^. Analytical validation figures such as repeatability, precision, selectivity, storage stability and accuracy were obtained. Different concentrations of S1-RBD were magnetically immobilized onto surface of the MgNPs/S1-Ab/BSA|MED biosensor to determine the limit of detection in PBS buffer and negative saliva samples. Finally, the magnetic immunoassay for electrochemical detection of S-1 RBD in real samples was conducted using optimized conditions with thermally inactivated saliva.

### Application in SARS-CoV-2 Detection

The biosensor’s functionality for detecting SARS-CoV-2 S1-RBD was tested using a DPV method. *Preparation of the Bioconjugate:* 20 μL of MnFe_2_O_4_-EDC/S1-Ab/BSA was mixed with 25 μL of S1-RBD (known concentration)) and incubated for 15 min. The bioconjugate was collected and washed with PBS using a neodymium magnet (NdFeB, Ø = 4 mm). *Modification of the Working Electrode:* 20 μL of the bioconjugate dispersion was added to the working electrode’s surface and dried for 120 min. *Electrochemical Measurements:* The modified electrode was immersed in a solution of K_4_[Fe(CN)_6_]/K_3_[Fe(CN)_6_] and measurements were performed using a miniaturized electrochemical device. The electrochemical detection of the SARS-CoV-2 S1-RBD in the saliva samples was performed in the same way. Thus, 15 μL of MnFe_2_O_4_-EDC:NHS/S1-Ab/BSA bioconjugate were dispersed in 5 μL of samples saliva and incubated for 15 min. After this reaction, MnFe2O4-EDC:NHS/S1-Ab/BSA/S1-RBD nanomaterial was collected and washed three times with PBS (1x, pH 7,4) using a neodymium magnet. Subsequently, 20 μL of MnFe_2_O_4_-EDC:NHS/S1-Ab/BSA/S1-RBD dispersion was added on the working electrode surface for magnetic immobilization and drying of the bioconjugate for 120 min. After modification step, 250 μL of 5 mmol L^–1^ K_4_[Fe(CN)_6_]/K_3_[Fe(CN)_6_] (0.5 mol L^–1^ KCl) was added in the magneto-integrated cell for electrochemical measurements. The presence of S1-RBD was detected by monitoring changes in the electrochemical signal.

### Ethical Approval and Saliva Collection

The collection and use of patient saliva samples were approved by the Ethics Committee under the approval code CONEP-B-16, as part of the analysis of diagnostic proposals on the Brazilian platform CNEP (*Conselho Nacional de Pesquisa Ética e Clínica).* With *n*:60, the samples were inactivated by natural means and thermal treatment at 65 °C for 10 min. Saliva samples were obtained from healthy donors and SARS-CoV-2 positive donors, confirmed by RT-PCR. These samples encompassed a wide range of symptoms, from asymptomatic individuals to those with low viral loads and severe COVID-19, providing a comprehensive representation of the COVID-19 population.

### Recyclability Rate Calculation

The recyclability rate can be calculated by evaluating the proportion of materials that can be recovered and reused after the battery has reached the end of its life (Supplementary Figure 2 and Supplementary Table 1). The steps are as follows: *Identify Recyclable Components:* List all components of the battery that can be recycled. This typically includes metals, plastics, and other recoverable materials. *Determine the Mass of Each Component:* Measure the mass of each recyclable component. *Total Mass of Recyclable Materials:* Sum the mass of all recyclable components. *Total Mass of the Battery:* Measure the total mass of the battery. *Calculate the Recyclability Rate:* Use the following formula:
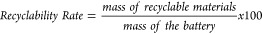
3

## Results and Discussion

### Sustainable Biosensor

After assembling the device (see details in Figure 1a), we conducted an in-depth characterization study of the biosensor. In Figure 1b, CV studies were conducted with the 5:5 mmol L^–1^ K_3_[Fe(CN)_6_]/K_4_[Fe(CN)_6_] redox probe in 0.5 mol L^–1^ KCl to investigate the electrochemical functionality of the developed device. For the unpolished and untreated device (MEDUn), there was practically no redox process observed, which was expected since the carbon particles are very compacted and completely encapsulated by nonconductive polystyrene (Figure S3). Additionally, the contact angle value after interaction with water molecules was 92.31 ± 0.34° (Figure S4), characteristic of hydrophobic surfaces.

However, after mechanical polishing, the electrode (MEDPun) showed a well-defined redox pair attributed to the oxidation/reduction between the [Fe^II^(CN)_6_]^4–^ and [Fe^III(^CN)_6_]^3–^ species. To further improve the electrochemical response, the device (MEDAT) was subjected to an electrochemical process in an electrolyte medium containing 1 mol L^–1^ NaOH. After this procedure, the electrode exhibited better electrochemical behavior, both in terms of lower peak potential separation (Δ*E*p) and higher current density (Figure S5). It is important to emphasize that even though Δ*E*p = 104.98 ± 2.25 mV for MEDAT is above the ideal value for reversible systems (57 mV ≤ Δ*E*p ≤ 60 mV),^18^ the prepared device performs much better than similar systems reported in the literature.^19,20^ These results demonstrate that the developed electrochemical cell is sensitive to redox probes, making it promising for detection assays.

The 3D-printed electrodes before and after treatment were also analyzed by EIS. Nyquist plots were used to investigate the charge transfer resistance (Rct) of the prepared 3D devices, as shown **in**Figure 1c. It can be noted that the inserted figure shows the equivalent Randle’s circuit, adjusted with solution resistance (Rs), constant phase element (CPE), Rct, and Warburg impedance (Zw). The CPE represents nonideal capacitance behavior due to surface heterogeneity and porosity of the electrode. Zw refers to the diffusion or transport process of species occurring at the electrode|electrolyte interface during the redox reaction. The MEDUn surface showed the highest Rct value (2730 ± 107.65 Ω cm^2^), consistent with the electrochemical response exhibited by CV. For this situation, the proposed equivalent circuit differs by the absence of Zw (Supplementary Figure 6). Meanwhile, the estimated Rct for MEDPun and MEDAT were 34.52 ± 6.1 and 14.93 ± 4.9 Ω cm^2^, respectively. These results indicate that the electrode after electrochemical treatment in alkaline medium presents faster electron transfer kinetics. The study of the best percentage composition between carbon and polystyrene (%C/PS) that provides good electrical conductivity and enhanced electrochemical performance was also conducted (Supplementary Figures 7–10). Thus, electrodes obtained from conductive inks of 40, 50, 60, 70, and 80%C/PS were analyzed. As shown by the data on current density, Δ*E*p, and Rct, the device with a 70% composition exhibited the best performance for use in electrochemical detection assays.

The morphology and elemental microanalysis of MEDPun and MEDAT were examined by SEM (Figures 1**d and**1**f**). The micrograph for MEDPun showed a surface formed by granules and plate-like structures like graphite, which were connected by polystyrene. EDX data showed that these structures are basically composed of carbon and oxygen. This morphology indicates that the graphite particles are not readily available for the electrode with only mechanical polishing performed. However, after electrochemical treatment (Figure 1f), the electrode surface had increased exposure to graphite plates and increased porosity. Another important finding is that the amount of oxygen increased. Consequently, the atomic ratio between O and C also increased, from 0.009 (MEDPun) to 0.03 (MEDAT). This behavior is possibly associated with the formation of oxygen-containing functional groups or the intercalation of OH^–^ ions between graphite layers. Additionally, the average contact angle value of the surface decreased to a typical value of hydrophilic materials. Raman spectroscopy was used to study the defects and degree of organization of the graphite carbon structures of MEDPun and MEDAT (Figures S11–12). For both electrodes, the appearance of D, D’, G, and 2D bands was observed. The D and D’ bands are attributed to the disordered mode of carbon edges, while the G band refers to the doubly degenerate phonon mode (E2g symmetry) of the sp^2^-ordered carbon atoms in the plane.^21^ On the other hand, the 2D band is related to the stacking of carbon layers and, consequently, to the number of graphene layers. Thus, the intensity ratio between the D and G bands (I_D_/I_G_) is generally interpreted as the degree of order/disorder of the carbon structure. The I_D_/I_G_ values for MEDPun and MEDAT are 0.22 and 0.35, respectively. The increase in I_D_/I_G_ for MEDAT suggests that electrochemical treatment in an alkaline and residual magnetization (Mr) medium promotes graphite oxidation and introduces oxygen functional groups. The hypothesis of the presence of functional groups can cause sp^2^ disorder at the edges of the graphite carbon structure. Through the ratio between the G and 2D bands (I_G_/I_2D_), the structural organization in terms of graphite carbon layers was analyzed. It is important to note that the lower I_G_/I_2D_ (1.81) value for MEDAT favors an organizational behavior with more layers, a spectroscopic finding consistent with SEM images showing the presence of several graphite plates.

Figure 1h shows the CVs recorded as a function of scan rate from 10 to 100 mV s^–1^. The plot of peak current densities versus the square root of the scan rate (Figure S13) revealed a linear profile, confirming the typical diffusion-limited behavior of the [Fe^II^(CN)_6_]^4–^/[Fe^III^(CN)_6_]^3–^ redox probe.^22^ The dependence of Δ*E*p and the ratio of peak current densities (jpa/jpc) indicates that the electron transfer process is quasi-reversible (Figure S14). The manufacturability of devices with reproducible electrochemical properties was analyzed by CV and DPV, as shown by the data in Figures 1**(i) and (j)**. Results of anodic peak current densities for 10 devices prepared on different days showed a relative standard deviation (RSD) below 5%, indicating that the manufacturing process is quite reproducible. Based on this good characteristic, we prepared 100 devices to conduct all experimental stages of the work. The electrodes exhibited very similar electrochemical performance (Figure S15) and did not show operational limitations even after successive reuse cycles. The electrochemical stability of the device was also analyzed by its good ability to respond to 100 successive scan cycles, evidenced by little change in the electrochemical profile (Figure 1**(l))**. Finally, the study in different pHs demonstrated that the electrode can operate in acidic, neutral, and basic media (Figure 1**(m))**. The peak potentials varied little with pH, while the lowest current densities were observed at more alkaline pH due to the electrostatic repulsion between OH- ions and [Fe^II^(CN)_6_]^4–^/[Fe^III^(CN)_6_]^3–^ species (Figures 16–17). One of the most relevant data from the pH study was the best system performance at pH 7.0, a condition close to physiological and suitable^23^ for assays with SARS-CoV-2 biomolecules and real samples.

### Magnetic Nanoparticles for Virus Capture

**Figure 2:**
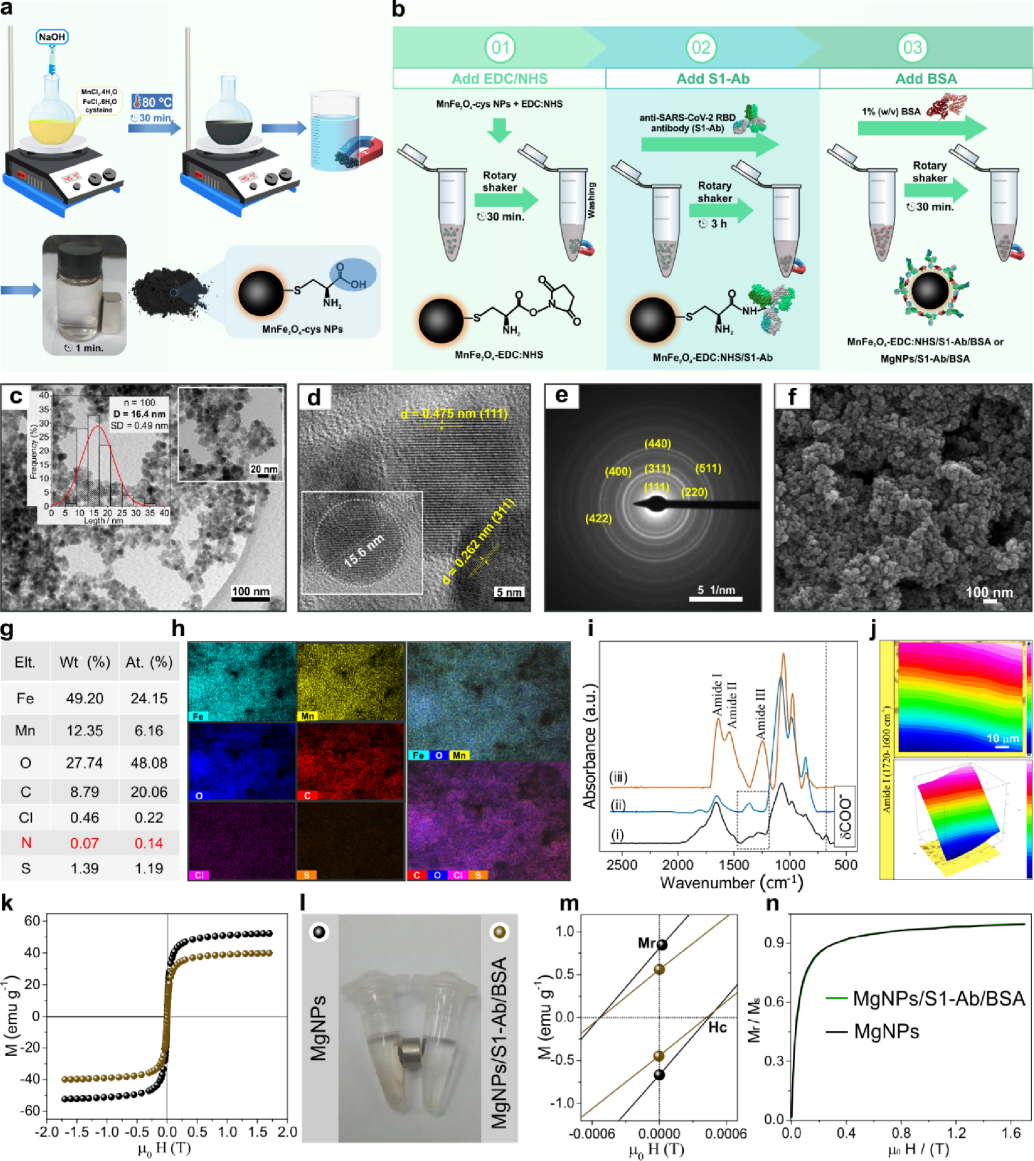
**Magnetic nanoparticles for virus capture. (a)** Schematic illustration for preparation l-cysteine functionalized MnFe_2_O_4_-cys NPs. (b) Schematic representation of the steps for synthesis of the MnFe_2_O_4_-EDC:NHS/S1-Ab/BSA bioconjugate. (c) TEM images with a log-normal nanoparticle size distribution histogram (inset). (d) HRTEM image and lattice fringes. (e) SAED pattern of the MnFe_2_O_4_–EDC:NHS/S1-Ab. **(f-h)** SEM image, chemical composition data obtained by EDX, corresponding EDX elemental mapping of Fe, Mn, O, C, Cl, S, Fe–O–Mn, and C–O–Cl-S of the MnFe_2_O_4_-EDC:NHS/S1-Ab. **(i)** FTIR spectrum of (i) MnFe_2_O_4_, (ii) MnFe_2_O_4_-EDC:NHS, and (iii) MnFe_2_O_4_-EDC:NHS-S1-Ab. **(j)** Micro-FTIR spectroscopy–2D chemical image obtained of the region of amide I, and corresponding 3D chemical mapping. **(k)** Magnetization curves of MnFe_2_O_4_-cys NPs and MnFe_2_O_4_-EDC:NHS-S1-Ab/BSA tested at room temperature. **(l)** photographs of aqueous dispersions of MnFe_2_O_4_-cys NPs and bioconjugate in the presence of a neodymium magnet. **(m)** zoomed of the magnetization curves for estimate the coercive force (Hc) and residual magnetization (Mr). **(n)** Remaining ratio (Mr/Ms) for MnFe_2_O_4_-cys NPs and bioconjugate.

The synthesis and characterization of a novel MnFe_2_O_4_-EDC/S1-Ab/BSA bioconjugate were designed to conduct magnetic immunoassays (Figures 2**a and b**). In Figure 2(a), the preparation of carboxyl-functionalized MnFe_2_O_4_ nanoparticles (MgNPs) was performed to allow stable covalent bonding with the anti-SARS-CoV-2 RBD antibody (S1-Ab). Figure 2(b) represents the modification steps for synthesizing the magnetic bioconjugate decorated with S1-Ab. The MgNPs-COOH were chemically conjugated with EDC/NHS to immobilize the S1-Ab antibodies covalently. In the final step, BSA was added to the reaction system to block unreacted NHS-ester groups, minimizing nonspecific interaction sites. The developed magnetic bioconjugate was used to detect the SARS-CoV-2 RBD antigen (S1-RBD). Thus, different characterization techniques were used to investigate the formation of the proposed bioconjugate. The phase composition and crystalline structure of the synthesized MgNPs were investigated by X-ray diffraction (XRD). The XRD pattern of MgNPs in Figure S18 showed the main reflection peaks that can be indexed to the cubic inverse spinel phase with space group *Fd*3̅*m* (ICSD card No. 43462). The average crystallite size (D, in nm) was calculated from Debye–Scherrer formula (eq 1). The average value was 14.28 ± 3.53 nm, confirming the formation of crystalline MnFe_2_O_4_ nanoparticles. Using the interplanar spacing value (d_311_, 2.54 Å) from the most intense peak, the lattice parameter a was estimated from eq 2. The calculated value of a is 8.42 Å, practically equal to the theoretical parameter (a = 8.45 Å) characteristic of the MnFe_2_O_4_ crystal cell from ICSD card No. 43462. The diffractogram exhibited intense, sharp, and broad Bragg peaks, indicating good crystallinity and small crystallites. Another important piece of information from the XRD data is that cysteine modification did not alter the crystalline structure of manganese ferrite. Additionally, no other diffraction peaks were observed, implying the preparation of high-purity MnFe_2_O_4_ NPs.

Transmission electron microscopy (TEM) and high-resolution TEM (HRTEM) images were used to investigate the shape and crystallographic features of the MnFe_2_O_4_-cys NPs and bioconjugate. TEM images in Figure S19(a) revealed the formation of spherical and quasi-spherical nanoparticles of various sizes. The distribution histogram showed an average diameter of 15.7 ± 4.6 nm with sizes ranging from 5 to 31 nm, which was estimated using ImageJ software. It is important to note that the average size was similar to that calculated by XRD. HRTEM micrograph provided a more detailed view of the planes with a lattice fringe (d) value of 0.256 nm (2.56 Å), corresponding to the (311) crystal plane of MnFe_2_O_4_ (Figure 19(b)). The selected area electron diffraction (SAED) pattern showed well-defined concentric rings, as shown in Supplementary Figure 19(c). This behavior was consistent with the (111), (220), (311), (400), (422), (511), and (440) reflection planes indexed in the XRD results.

The discontinuous diffraction rings confirmed the polycrystalline nature of the MnFe_2_O_4_–NPs. Surface morphology and chemical composition were also analyzed by SEM. The SEM micrograph of MgNPs (Figure 19(d)) shows that the nanoparticles formed polydisperse magnetic clusters with a quasi-spherical morphology, as observed in the TEM images. The aggregates had an average diameter of 98.73 ± 4.6 nm, with sizes ranging from 32 to 239 nm. The presence of C, O, Mn, and Fe in the MgNPs was determined by energy-dispersive X-ray spectroscopy (EDX). The presence of C, O, Mn, and Fe in the MgNPs was determined by energy-dispersive X-ray spectroscopy (EDX). The percentage composition values (Figure S19(e)) obtained by EDX were higher for Fe and O, respectively, consistent with the stoichiometry of MnFe_2_O_4_. The atomic percent ratio for O/Mn of 5.16 is higher than the theoretical value^24^ due to chemical functionalization with cysteine. Elemental maps of Mn, Fe, and O indicated that these species were uniformly distributed in the structure, confirming the formation of MnFe_2_O_4_ NPs. The carbon atom was also uniformly distributed throughout the mapped window, suggesting the incorporation of cysteine on the nanoparticle surface (Figures S19(f) and (g)). To corroborate this organization, the micrograph in (Figure S19(h)) reveals an organic coating on the magnetic clusters. Additionally, the inserted SEM image clearly shows that the aggregates consist of small MnFe_2_O_4_ NPs.

TEM was also used to analyze the structural properties of the MnFe_2_O_4_-EDC:NHS-S1-Ab bioconjugate nanoparticles. Figure 2(c) shows that the MnFe_2_O_4_-EDC:NHS-S1-Ab nanoparticles mostly have a quasi-spherical shape. The distribution histogram showed an average diameter of 16.4 ± 0.49 nm with sizes ranging from 7 to 35 nm, which was quite like the isolated MgNPs. HRTEM image also showed the presence of a spherical nanoparticle structure, with estimated size of 15.6 nm. The lattice fringes were estimated to be 0.262 nm (2.62 Å) and 0.475 nm (4.75 Å), which correspond to (311) and (111) crystal planes of MnFe_2_O_4_, respectively. The SAED result showed a typical behavior of a polycrystalline material with crystallographic planes attributed to cubic spinel structure of the MnFe_2_O_4_. The SEM results (Figure 2**(f**)) for the MnFe_2_O_4_-EDC/S1-Ab bioconjugate showed a different distribution and organization compared to the MgNPs. In this case, the SEM micrograph revealed a denser and more uniform formation of aggregates consisting of smaller nanoparticles. The nanoparticles were also nearly spherical and had an estimated length of 29.11 ± 0.86 nm, with an average diameter approximately 3.4 times smaller than that of the MgNPs (Figure S20). The sizes ranged from 12.8 to 51.5 nm, exhibiting a narrower and more homogeneous distribution in nanoparticle dimensions. EDX data for the bioconjugate nanoparticles showed significantly different behavior compared to the MgNPs, notably the appearance of new chemical elements (Figure 2(g)).

In this case, Cl and S were related to the chemical structures of EDC, NHS, and S1-Ab. The atomic percent ratio for O/Mn of 7.80 was higher than the theoretical value due to the incorporation of other oxygen-containing chemical components in the structure. Another interesting finding was the decrease in N to below the detection limit of the technique, compared to the MnFe_2_O_4_-EDC:NHS nanomaterial (Figures 21–23). The significant variation in N percentages was expected, as the S1-Ab antibody chemically binds to the surface of MnFe_2_O_4_-EDC via amide bonds (MnFe_2_O_4_-ROC-NH-R’-S1-Ab). As illustrated in the model in Figure 2(b), this covalent coupling occurs between the primary amines of S1-Ab and the carboxylate groups activated by EDC/NHS. The chemical map of the Fe–O–Mn elements revealed a uniform distribution related to the MnFe_2_O_4_ NP structure (Figure 2(h)). In contrast, the EDX mapping of C, O, Cl, and S was associated with the chemical composition of EDC, NHS, and the antibody. These observations suggest that the S1-Ab antibody was uniformly organized across almost the entire mapped microregion.

To confirm the conjugation with S1-Ab on the surface of the magnetic nanoparticles, FTIR spectroscopy was conducted to investigate the functional chemistry of the prepared nanomaterials. The FTIR spectra of MgNPs, MgNPs-EDC:NHS, and MgNPs-S1-Ab are shown in Figure 2**(i)**. For the MgNPs, peaks appeared at 1351, 1286, and 676 cm^–1^, attributed to COO^–^ stretching, C–O stretching, and COO^–^ deformation, respectively. Additionally, the FTIR spectrum for MgNPs from 4000 to 400 cm^–1^ (Figure S24) identified other vibrational modes, including asymmetric NH_3_^+^ stretching (1641 cm^–1^), Fe–O stretching (569 cm^–1^), and Mn–O stretching (452 cm^–1^). These chemical groups are characteristic of cysteine and manganese ferrite, suggesting chemical coupling between these (nano)materials. The absence of the absorption band around 2500 cm^-1^ corresponding to the cysteine S–H group was attributed to the Fe-SH interaction on the MnFe_2_O_4_ surface. The spectrum of MgNPs-EDC:NHS exhibited stretching of the COO-NHS groups (1656 cm^–1^), N–O (1081 cm^–1^), and NHS (985 and 861 cm^–1^), indicating modification with EDC and NHS. An important aspect is the disappearance of δCOO^–^, demonstrating chemical activation and interaction by the carboxylate groups. The chemical immobilization of S1-Ab in the bioconjugate was observed from the emergence of amide I (1639 cm^–1^), amide II (1540 cm^–1^), and amide III (1243 cm^–1^) stretching vibrations. Micro-FTIR analyses of the MgNPs-S1-Ab bioconjugate on a gold substrate were conducted to chemically visualize the antibody’s amide bands (Figure S25). The interpretation of chemical maps is based on the correlation between colors and intensity, with magenta indicating higher concentration and blue representing lower concentration of the band. Therefore, the heterogeneous spatial distribution of the amide I band (Figure 2**(j)**) shows that the protein aggregates of the bioconjugate accumulated at the edges due to the coffee-ring effect, typical of drop-casting immobilization on substrates.

The magnetic behavior of the MgNPs and MgNPs-S1-Ab/BSA bioconjugate was investigated using vibrating sample magnetometry (VSM) conducted at room temperature, as shown in Figure 2**(k)**. It can be observed that the saturation magnetization (Ms) values for the MgNPs and the bioconjugate were estimated at 48.83 ± 0.15 and 39.07 ± 0.03 emu g^–1^, respectively. It is interesting to note that even after successive modifications with nonmagnetic materials, the bioconjugate still presented a considerable Ms value (Table S3). This means that the bioconjugate can be easily separated from the reaction medium. The hysteresis curves exhibited insignificant values of coercivity (Hc) and remanent magnetization (Mr), classifying both nanomaterials as soft and superparamagnetic (Figure 2**(m)**). The magnetic field value of the 3D printed magneto-integrated electrochemical device (MED) at each position is shown in Supplementary Figure 26. The increase in the remanent ratio (Figure 2**(n)**) for the two analyzed materials indicated favoring magnetic dipolar interactions. These characteristics are responsible for the superparamagnetic bioconjugate not retaining residual magnetism, making it an excellent model for practical applications in magnetic immunoassays.

**Figure 3:**
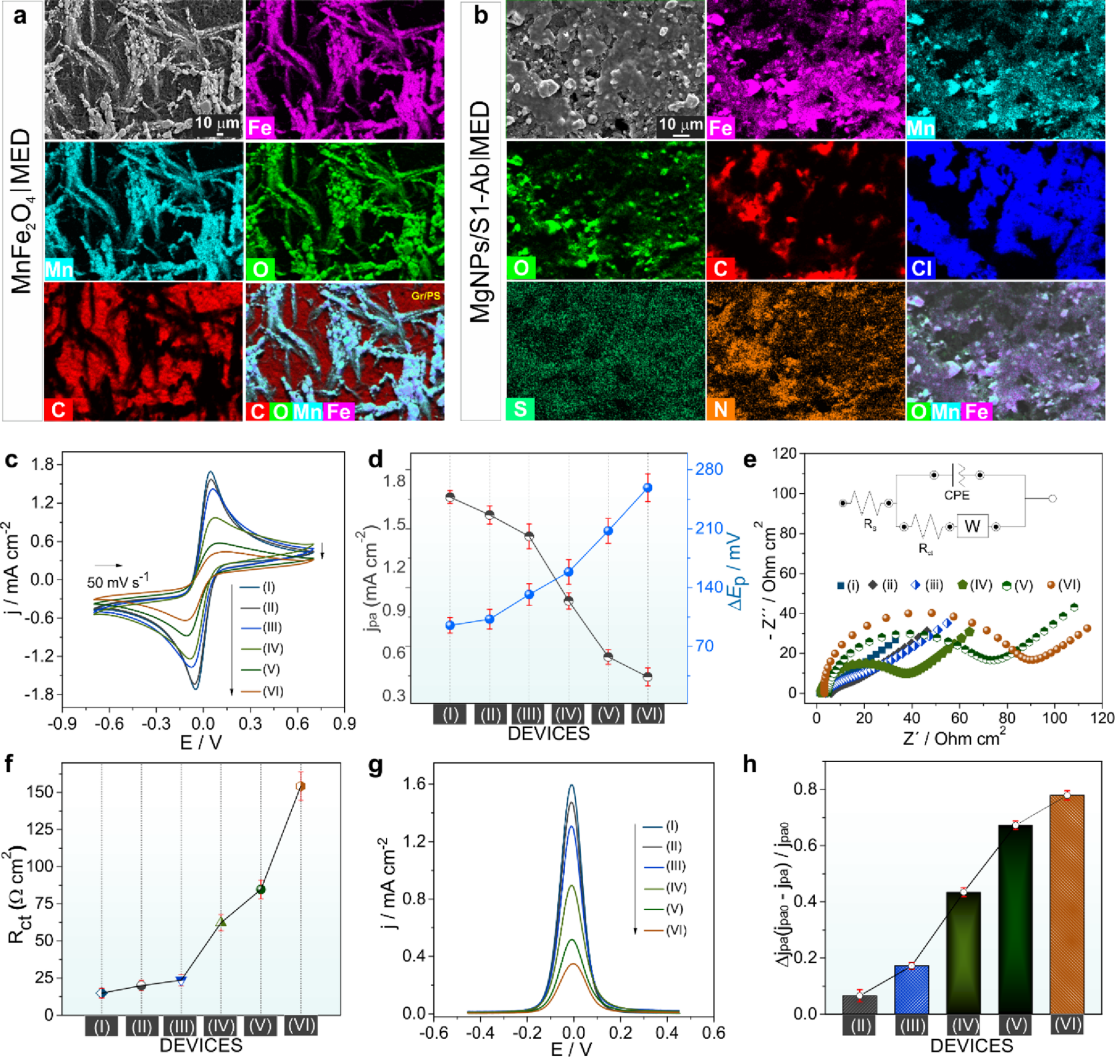
**Biosensor performance. (a)** SEM of the mapped area and EDX elemental mapping of Fe, Mn, O, C, and C–N–Mn-Fe of the MnFe_2_O_4_|MED device. (b) SEM of the mapped area with corresponding EDX elemental mapping of Mn, O, C, Cl, S, N, and O–Mn–Fe of the MgNPs/S1-Ab|MED. (c) CV responses at 50 mV s^–1^ for different (bio)devices (i) bare MED, (ii) MnFe_2_O_4_|MED, (iii), MnFe_2_O_4_-EDC:NHS|MED, (IV), MgNPs/S1-Ab|MED, (V) MgNPs/S1-Ab/BSA|MED (VI) MgNPs/S1-Ab/BSA/S1-RBD|MED (0.5 ng mL^–1^ S1-RBD). (d) jpa0 and Δ*E*p versus (bio)devices. (e) Corresponding EIS measures of the (bio)devices. **(f))** Rct versus (bio)devices. (g) DPV responses of the (bio)devices. (h) Corresponding decrease of the anodic peak current densities (jpa0–jpa/jpa0) versus (bio)devices. 95% confidence intervals for all error bars (*n* = 3). All electrochemical study was performed in 5.0 mmol L^–1^ K_3_[Fe(CN)_6_]/K_4_[Fe(CN)_6_] (0.1 mol L^–1^ PBS, pH 7.4).

The morphologies of the electrodes based on recyclable graphite conductive ink before and after modification with S1-Ab are shown in Figures 3**(a) and (b)**, respectively. For the MnFe_2_O_4_|MED device, a rough and porous surface formed by aggregates of nanoparticles supported on graphite microplates connected by polystyrene was observed. Micrographs at higher magnifications revealed the presence of graphite microplates/sheets and nearly spherical MnFe_2_O_4_ NPs with an average size of 36.14 ± 2.8 nm (Figure S27). The EDX spectrum identified signals of Fe, Mn, O, and C, confirming the chemical composition of the cysteine-functionalized MnFe_2_O_4_ NPs (Figure S28). The high value for C in the elemental percentages is notable, resulting from the contribution of carbon microparticles. Consequently, the atomic ratio between C/Mn increased significantly compared to the analysis of MnFe_2_O_4_ NPs dispersion, from 1.18 to 34.81. Individual elemental mapping in Figure 3a displayed a uniform distribution of all elements in the nanoparticle aggregate microregion. The overlaid map clearly showed that the superparamagnetic nanoparticles covered a substantial portion of the working electrode surface.

The surface morphology of MgNPs/S1-Ab|MED appeared more heterogeneous, identifying microregions of larger clusters, roughness, porosity, and smooth profile topography (Figure 3**(b))**. However, analyzing a more detailed region revealed the presence of graphite microplates and MnFe_2_O_4_ NPs aggregates surrounded by organic components, possibly EDC, NHS, and S1-Ab (Figure S29). The surface became more microstructured likely due to the probable formation of antibody aggregates. The smooth morphological behavior covering MnFe_2_O_4_ NPs aggregates was similar to that exhibited by MnFe_2_O_4_-EDC:NHS|MED, corroborating the chemical attribution to EDC and NHS (Figure S30). For MgNPs/S1-Ab|MED, an average size of 34.97 ± 1.9 nm was estimated for the MnFe_2_O_4_ NPs, which was quite like the size calculated for the nanoparticles of the MnFe_2_O_4_|MED device (Figure S31). The EDX spectra for MnFe_2_O_4_-EDC:NHS|MED and MgNPs/S1-Ab|MED detected the elements Fe, Mn, O, C, Cl, S, and N (Figure S32 and Figure S33). Since Cl is a chemical element of EDC, the atomic ratio between Cl/Mn was used to infer the presence of S1-Ab. Thus, a considerable decrease from 21.65 to 2.85 was observed after the nanoparticle modification with the antibody. The atomic ratio of N/Mn decreased even further from 78.03 to 1.40 after S1-Ab incorporation, indicating the conjugation of the antibody with the MnFe_2_O_4_-EDC:NHS surface occurs preferentially via nitrogen, forming amide bonds. Individual chemical maps showed the distribution of the mentioned elements, with a more homogeneous and dense organization of S observed in the mapped area **(**Figure 3**(b))**. The mapping for O, Mn, and Fe atoms demonstrated that the MnFe_2_O_4_ NPs were organized over much of the analyzed surface. Additionally, the overlaid maps of N–S–Cl and O–N–S for the MnFe_2_O_4_-EDC:NHS|MED and MgNPs/S1-Ab|MED electrodes showed a distinct distribution, with notable highlights for Cl and N (Figure S34 and Figure S35). All these results reinforce the FTIR spectroscopic information, confirming the bioconjugation of the antibody to the nanoparticles via EDC/NHS coupling chemistry.

### Biosensor Characterization

Electroanalytical techniques (CV, EIS, and DPV) were used to investigate the successive modifications and the effect of the antibody–antigen interaction on the electrochemical behavior of the MgNPs/S1-Ab/BSA|MED biosensor. In Figure 3(c), all CVs of the modified electrodes exhibited a decrease in oxidation and reduction responses. The most pronounced changes were for electrodes modified with S1-Ab, BSA, and S1-RBD. This electrochemical behavior can be attributed to the incorporation of bulky molecules on the device surfaces and the probable electrostatic repulsion of deprotonated functional groups with the redox probe [Fe^II^(CN)_6_]^4–^/[Fe^III^(CN)_6_]^3–^. These factors act to hinder and prevent electron transfer to the electrode surfaces. The most significant current decrease in the MgNPs/S1-Ab/BSA/S1-RBD|MED electrode suggests that effective interaction occurred between the target protein S1-RBD and the recognition antibody S1-Ab. Figure 3(d) shows that with each modification step, the anodic current density (jpa) decreased accompanied by an increase in Δ*E*p.

In addition to CV data, EIS information was used to investigate the electron transfer resistance after working electrode surface modifications. The results were discussed from Nyquist plots (Figure 3(e)). As shown in the **inset figure**, an equivalent circuit configuration, [Rs([RctW]CPE)], was proposed for all devices. In higher frequency regions, the semicircular profile of EIS measurements became more pronounced at each modification configuration. As expected, Figure 3(f) exhibits the increasing behavior of electron transfer resistance values, aligning with the trends observed in CVs. To further confirm the modifications, DPV voltammograms exhibited successive decreases in jpa (Figure 3(g)). Similar to the other electrochemical data, the MgNPs/S1-Ab/BSA/S1-RBD|MED electrode showed a more significant decrease in jpa. This behavior can be better analyzed by the graphical relationship between the decrease in anodic current density (Δjpa = jpa0 - jpa/jpa0) versus device type (Figure 3**(h))**. The relationship between the obtained electrochemical properties confirms that modifications occurred with the respective selected materials. Additionally, these electrochemical results suggest that an interaction likely occurred between S1-RBD and the antibody immobilized on the biosensor. In this context, a study was conducted to optimize experimental conditions (Figure S36 and Figure S37). In particular, the antibody, being the most expensive material of the biosensor and affecting the redox probe’s electrochemical response, was studied. Bioconjugates with different concentrations of S1-Ab (10, 15, 20, 25, and 30 μg mL^–1^) were prepared. The concentration of 20 μg mL^–1^ S1-Ab was selected as it presented the highest Δjpa value. Another optimization parameter studied was the incubation time for the interaction between S1-RBD and the MgNPs/S1-Ab/BSA|MED biosensor. DPV measurements were obtained between 5 and 60 min. The time of 15 min represented the highest reactivity index between the antibody and the target protein. Therefore, subsequent magnetic immunoassays were performed with 20 μg mL^–1^ S1-Ab and an incubation time of 15 min.

### Electroanalytical Performance of the Biosensor

**Figure 4:**
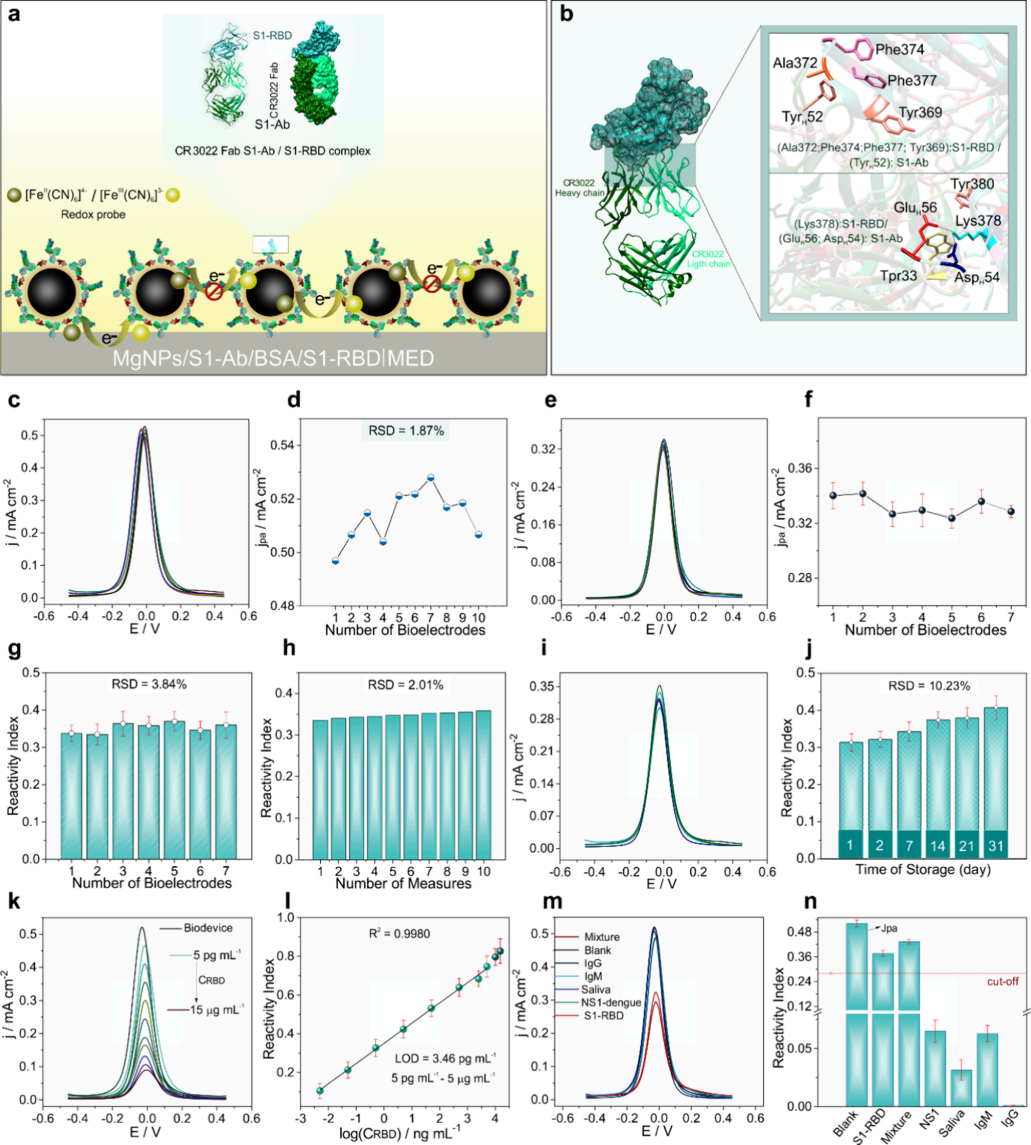
**SARS-CoV-2 S1-RBD detection and interferences. (a,b)** Schematic illustration of the current inhibition effect based in the formation of the CR3022(Fab)/SARS-CoV-2 S1-RBD complex onto MgNPs/S1-Ab/BSA|MED surface (c) DPV responses for different MgNPs/S1-Ab/BSA|MED bioelectrodes. (d) jpa versus bioelectrodes. (e) DPV responses for seven Bioelectrodes +0.5 ng mL^–1^ of SARS-CoV-2 S1-RBD (interelectrode). (f) Corresponding jpa versus bioelectrodes. (g) Corresponding reactivity index for seven bioelectrodes. (h) Reactivity index for ten bioelectrodes at (0.5 ng mL^–1^) of SARS-CoV-2 S1-RBD. **(i)** Long-term stability for different bioelectrodes storage by 30 days. **(j)** Corresponding reactivity index for long-term study. **(k)** DPV responses of the biosensor after incubation with different concentrations of S1-RBD. **(l)** Linear calibration plot for SARS-CoV-2 S1-RBD. **(m)** Cross-reactivity assays of the as-prepared bioelectrode after incubation with some off-target species (dengue NS1, IgM, IgG, at 5.8 ng mL^–1^), saliva (1:1 v/v) and a mixture (including the S1-RBD-0.58 ng mL^–1^). **(n)** Respective reactivity index for cross-reactivity study. All electrochemical experimental were realized with [Fe(CN)_6_]^4–^/[Fe(CN)_6_]^3–^ (5 mmol L^–1^, 0.1 mol L^–1^ PBS, pH 7.4). 95% confidence intervals for all error bars (*n* = 3).

Figure 4 illustrates the detection mechanism and key analytical parameters for validating the new S1-RBD biosensing method. Figures 4**(a) and (b)** are proposed models to formation of the CR3022FabS1-Ab/S1-RBD complex that occurs on the surface of the MgNPs/S1-Ab/BSA bioconjugate nanoparticles. The crystal structure of this type of complex has already been elucidated (PDB: 6W41) and widely discussed in other studies.^25−27^ A more detailed view shows that the interaction interfaces mainly occur through the amino acid residues of the heavy chains of CR3022 and the epitope residues of S1-RBD. In summary, the global interaction for the formation of CR3022FabS1-Ab/S1-RBD is governed by hydrophobic interactions, electrostatic interactions, and hydrogen bonding. The buried surface area of the complex is approximately 917 Å^2^ (9.17 nm^2^), creating structural blocks that hinder electron transfer on the electrode surface.

The repeatability for biosensor preparation was investigated by evaluating the electrochemical response of the redox probe [Fe(CN)6]^4–^/^3–^ obtained from ten independently prepared MgNPs/S1-Ab/BSA|MED bioelectrodes (Figure 4(c)). The interelectrode repeatability study’s jpa graphs revealed an RSD value of only 1.87% (Figure 4(d)), confirming excellent repeatability in biosensor fabrication. The detection repeatability for different bioelectrodes was evaluated through seven magnetic immunoassays in the presence of 0.5 ng mL^–1^ of SARS-CoV-2 S1-RBD (Figures 4**(e)-(h)**). No significant changes were observed in the DPVs profiles in terms of electrochemical behavior and jpa (Figures 4**(e) and (f)**). The calculated RSD for the reactivity index of the immunoassays was only 3.84%, demonstrating the biosensor’s high precision in detecting S1-RBD. Additionally, the capability of a single MgNPs/S1-Ab/BSA|MED biosensor to respond to ten immunoassays conducted with the same methodology was analyzed. The DPVs profiles of the intraelectrode repeatability immunoassays showed no significant electrochemical changes (Figure S38). In this case, the reactivity index of the measurements presented a very low RSD value (2.01%) (Figure 4(h)), indicating excellent repeatability for the same biosensor used. The biosensor’s stability was examined after monitoring the electrochemical response over a storage period of MgNPs/S1-Ab/BSA|MED bioelectrodes for 31 days at 4 °C. As shown in Figure 4**(i**), jpa decreased with the increase in the storage period. However, jpa exhibited good precision among the measurements with an RSD of 5.51% (Figure S39). In Figure 4**(j)**, the reactivity index analysis indicated a satisfactory RSD value (10.23%). These results indicate that the proposed biosensor is highly suitable for precise and reliable detection in point-of-care situations.

The effect of S1-RBD concentration on the DPV electrochemical response of the MgNPs/S1-Ab/BSA|MED biosensor was investigated to construct the calibration curve of the developed method. Figure 4**(k)** shows the DPVs after incubation with different S1-RBD concentrations (5 pg mL^–1^ to 15 μg mL^–1^) in a solution containing the redox probe [Fe(CN)_6_]^4–^/[Fe(CN)_6_]^3–^ at pH 7.4. The jpa decreased with increasing S1-RBD concentration. The reactivity index versus log(CRBD) plot showed a linear behavior in a wide range of 5 pg mL^–1^ to 5 μg mL^–1^ (Figure 4**(l)**). At concentrations greater than 5 μg mL^–1^, the available sites for protein binding to the bioconjugate antibody likely became saturated. Additionally, the limit of detection (LOD) was estimated using the equation LOD = 3s_y/x_/m, where s_y/x_ and m are the standard deviation of the intercept and the slope obtained from the calibration curve, respectively. Thus, a low detection limit of 3.46 pg mL^–1^ was calculated.

The overall electroanalytical performance of the biosensor was compared with modified working electrodes developed by other authors (Table S4). It was found that the MgNPs/S1-Ab/BSA|MED biosensor stood out by presenting an antibody-free transducer, operational simplicity, wide linear range, and low detection limit. Additionally, the low-cost biosensor performs analyses on real human saliva samples with a relatively short incubation time. To further confirm the proposed methodology’s validation, cross-reactivity studies with potential interferents that may be present in saliva samples were conducted (Figures 4**(m) and (n)**). Thus, the biosensor’s selectivity was tested with human IgG, human IgM, dengue NS1 protein, saliva (1:1 v/v), and a mixture of these components with S1-RBD. To evaluate the interference effect, the cutoff point was estimated based on the LOD for a concentration 10 times greater than 0.58 μg mL^–1^ of S1-RBD. For the studied interferents, the reactivity index values were insignificant, proving they do not effectively interact with the biosensing platform. The absence of cross-reactivity in saliva and dengue samples is a prominent fact since these are complex real matrices that usually lead to false positive and negative results. Only for S1-RBD and the mixture did the reactivity index exceed the calculated cutoff value, a completely expected behavior. The results show that the prepared biosensor has adequate selectivity and specificity for detecting S1-RBD in real saliva samples.

### Detection of SARS-CoV-2 S1-RBD in Human Saliva

**Figure 5:**
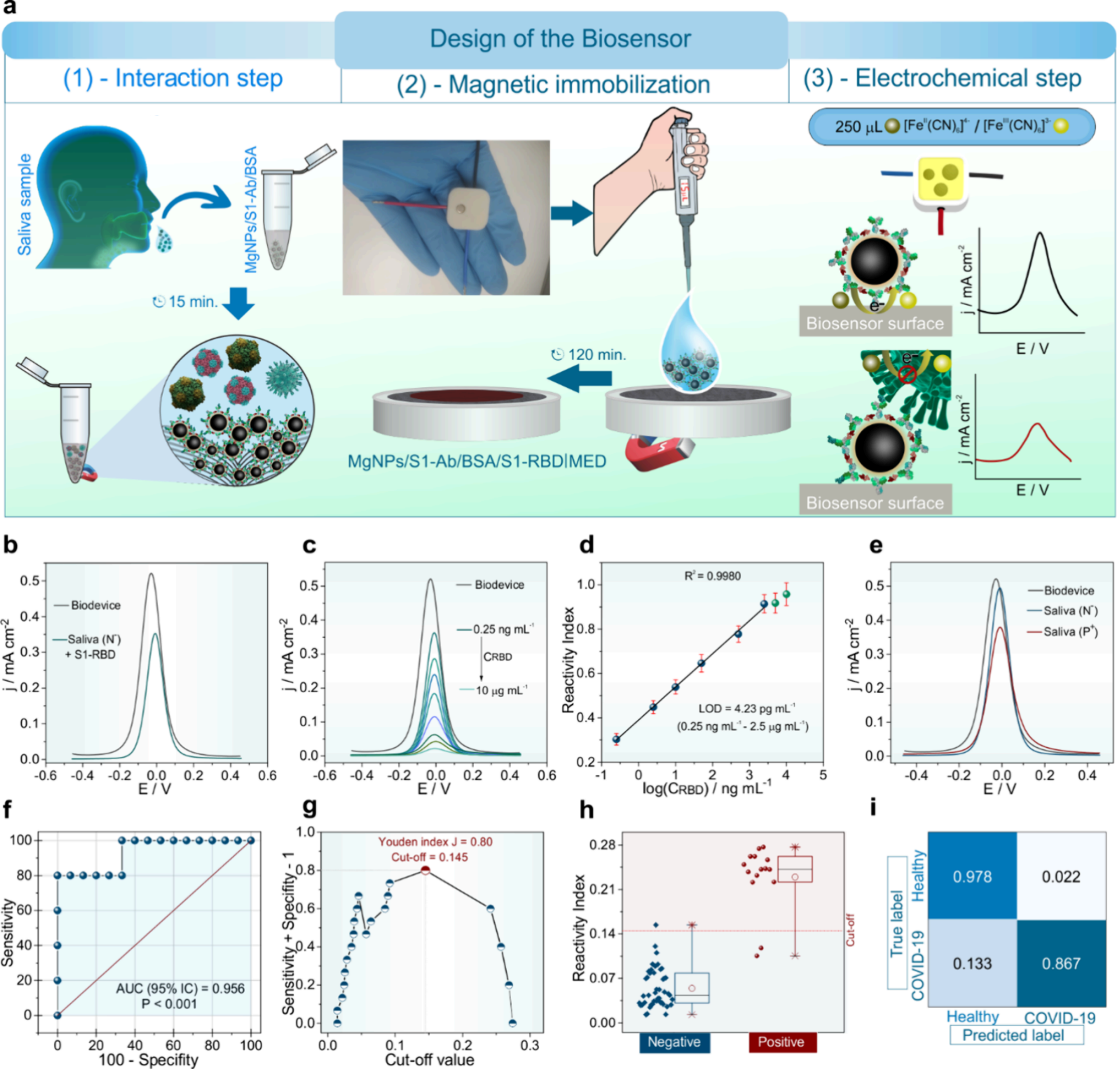
**Application of the MgNPs/S1-Ab/BSA|MED biosensor in saliva samples. (a)** Schematic of the setup for biosensor functioning in human saliva samples. (b) DPV for bare biodevice and biodevice + S1-RBD in negative saliva sample (N^–^) (c) DPV responses for biodevice in different concentrations of S1-RBD (0.25 ng mL^–1^ to 2.5 μg mL^–1^) N^–^. (d) Corresponding calibration curve obtained for S1-RBD in N^–^. (e) DPV voltammograms for N^–^ and positive saliva (P^+^) samples. (f) Receiver operating characteristic (ROC) curve for biosensor in inactive saliva samples. (g) Plot of Youden index J for determination of the cutoff value. (h) Corresponding boxplots of the data sets obtained for the assays performed for ten healthy individuals and five patients with SARS-CoV-2 virus, all for inactive saliva samples. **(i)** Respective confusion matrix for inactive samples. All error bars (*n* = 3) represent 95% confidence intervals. All electrochemical assays was performed in the presence of 5.0 mmol L^–1^ K_3_[Fe(CN)_6_]/K_4_[Fe(CN)_6_] (0.1 mol L^–1^ PBS, pH 7.4).

Figure 5 shows the application of the biosensor in real samples, evaluating how the matrix effect influences the performance of the MgNPs/S1-Ab/BSA|MED. Figure 5(a) is a schematic illustration of the miniaturized configuration that simplifies the operational steps of the MgNPs/S1-Ab/BSA biosensor in human saliva samples. The interaction step with the S1-RBD detection antigen is facilitated by the superparamagnetic properties of the MgNPs/S1-Ab/BSA bioconjugate. The enrichment and magnetic immobilization of the MgNPs/S1-Ab/BSA/S1-RBD immunocomplex on the electrode surface were feasible using an external magnet attached to the electrochemical cell. Another advantage is the elimination of the electrode surface washing step after interaction with the saliva matrix solution, making the procedure faster. Consequently, the strong magnetic interaction allowed conducting immunoassays without the need to dilute the samples. Another feature of the biosensor design is the ability to protect the upper part of the electrochemical cell using readily available materials, such as film paper or adhesive tape. This simple yet effective measure minimizes the risk of contamination of the bioconjugate/SARS-CoV-2 S1-RBD complex during the immobilization and drying stages. By ensuring sample integrity while relying on inexpensive and widely accessible materials, this approach significantly enhances the practicality and cost-effectiveness of the biosensor for mass testing, particularly in resource-limited regions. As previously explained (Figures 4**(a) and (b)**), the principle of electrochemical detection of the magnetic immunoassay is based on the recognition interaction between the CR3022(Fab) antibody and the SARS-CoV-2 S1-RBD.^23,24^

We investigated the impact of the matrix on the detection of the S1-RBD protein (Figure 5**(b))**. The differential pulse voltammograms (DPVs) of the biosensor are presented before and after interaction with negative saliva. Reading the DPVs shows a reduction in current density in response to the presence of the S1-RBD antigen. This electrochemical data is consistent with the proposed interaction between the biosensor and S1-RBD, emphasizing the previously discussed mechanism. The result highlights the biosensor’s high sensitivity to the protein’s presence, even in complex matrices such as saliva. Besides sensitivity, we also evaluated the biosensor’s behavior at different S1-RBD concentrations, as shown in Figure 5(c). The analysis shows how the biosensor’s reactivity varies with increasing S1-RBD concentration, demonstrating its sensitivity to different target analyte concentrations in negative saliva samples. The study shows the increase in S1-RBD concentration (CRBD) with the increment of the biosensor’s reactivity index (RI), highlighting its sensitivity to the protein’s concentration variation. The linear analytical range suggests that the biosensor can be successfully applied in samples with various S1-RBD concentrations. Thus, the proposed electrochemical device can be used in samples with different levels of viral protein concentration, making the biosensor promising for practical applications in various scenarios. The graph in Figure 5(d) of RI versus CRBD is the calibration curve in a negative saliva sample. A logarithmic behavior of RI as a function of CRBD was verified, with an excellent correlation coefficient (R^2^) of 0.9980, a wide linear range from 0.25 ng mL^–1^ to 2.5 μg mL^–1^, and a low LOD of 4.23 pg mL^–1^. Based on these parameters, the biosensor was tested in negative saliva samples enriched with low and high concentrations of S1-RBD. Thus, the recovery values ranged between 95.74 and 105% and RSD from 2.69 to 4.76% (Table S5), indicating good precision and that the complex saliva matrix did not significantly influence the electrochemical sensing of S1-RBD. The DPVs and results for the addition and recovery assays in negative samples are in Figure S40.

Based on the previously determined appropriate sensitivity of the biosensor, we proceeded to apply it in real samples in detecting SARS-CoV-2 S1-RBD. Figure 5(e) represents the comparative DPVs for the biosensor in PBS (pH 7.4), as well as in the presence of a negative sample and a positive sample. For systems containing negative saliva samples, there is no significant decrease in the redox probe current. However, a substantial reduction is seen in the presence of the positive sample. This means that the biosensor is sensitive and capable of differentiating between positive and negative samples. For this purpose, several biosensors were utilized with n= 60. The biosensor’s performance and applicability as a diagnostic test for COVID-19 were evaluated by estimating the ROC curve. Thus, the biosensor classification for the samples was compared with the RT-PCR gold standard data. Figure 5(f) shows the relationship between sensitivity (true positive rate) and specificity (true negative rate) at different cutoff values. The ROC curve analysis revealed an area under the curve (AUC) of 0.956 (*p* < 0.001), indicating excellent test classification performance. The ROC curve’s behavior shows that there is a trade-off between sensitivity and specificity (Table S6), that is, as sensitivity increases, specificity decreases. Furthermore, the AUC means that the test has a 93% probability of correctly classifying a SARS-CoV-2 patient, indicating low diagnostic errors. Through the curve, we also identified the optimal cutoff to maximize the test’s sensitivity and specificity **(**Figure 5**(g))**. This cutoff point was found at a value of 0.145, based on the highest Youden index (Supplementary Table 6). It is important to note that the cutoff value was maximized to improve the test’s sensitivity and specificity to avoid incorrect disease diagnoses. Therefore, based on the ROC curve data, we can conclude that the biosensor demonstrated satisfactory results for COVID-19 diagnosis with adequate sensitivity and specificity. The individual study of each sample was also conducted with several immunoassay replicates (n = 60).

Figure 5(h) shows the boxplots of the reactivity index for the studied samples. From this graph, it is evident that the biosensor was able to clearly distinguish and classify negative and positive samples. Despite the asymmetric distribution of the reactivity index values, no outliers were observed, indicating little effect of operational errors. However, based on these data, a false positive result and two false negatives were observed (Table S7). These results, which differ from RT-PCR classification, can be attributed to the thermal treatment to which the samples were subjected for virus inactivation. Possibly, the thermal procedure altered the protein structure of a certain concentration of S1-RBD, preventing its binding to the MgNPs/S1-Ab/BSA bioconjugate. Based on the tabulated data (Table S8), the biosensor showed good performance for sensitivity (86.67%), excellent specificity (97.78%), and accuracy (95%), as well as a precision of 92.86%. These parameters obtained show that the developed magnetic biosensor has a good correlation and diagnostic capability comparable to the RT-PCR gold standard molecular test. Despite the denaturation of a certain amount of S1-RBD in viral samples, the biosensor showed good sensitivity, making it a promising candidate for sensing biomarkers in real samples that need to be inactivated. The inactivation of infectious clinical samples is a point-of-care diagnostic trend because it offers significant advantages, including ease of transport and analysis in laboratories that do not require biosafety levels. Finally, the confusion matrix (Figure 5**(i)**) was also constructed, and the data demonstrated good ability to differentiate healthy individuals from those with COVID-19. The interpretation of this predictive model indicates that the true positive and false negative rates for the disease are 86.7% and 13.3%, respectively. The true negative and false positive rates for COVID-19 are 97.8% and 2.20%, respectively. Moreover, another factor that makes the proposed biosensor attractive is the manufacturing cost of approximately USD 0.2 (Table S9), which is significantly lower than currently available commercial COVID-19 diagnostics.

## Conclusions

The biosensor proposed in this work represents an innovative and practical solution for affordable and effective virus detection, with significant potential to expand diagnostic access in resource-limited regions. Utilizing recycled materials from spent batteries and plastics within a circular economy framework, the biosensor achieved a remarkable 98.5% recyclability rate, aligning with global sustainability goals. The device demonstrated high clinical efficacy, achieving a 95% correlation with the gold standard RT-PCR for COVID-19 detection, with a material cost of only USD 0.2 per test. Its robust electrochemical performance, characterized by a low detection limit of 3.46 pg mL^–1^, a wide linear detection range, and high selectivity, underscores its reliability.

The integration of 3D printing technology enabled the rapid and scalable production of the biosensor, allowing for decentralized point-of-care testing and reducing dependence on centralized laboratories. Additionally, its reusability and compatibility with complex sample matrices, such as human saliva, further enhance its applicability in real-world scenarios.

By combining high sensitivity, specificity, and environmental benefits, this biosensor addresses critical healthcare inequities while minimizing environmental impact. Beyond COVID-19, the modular design and adaptability of this platform offer opportunities for detecting a broad spectrum of viral diseases.

## Supporting Information Available

Complete protocols; materials used for fabrication of thousand MED; strategy used to select recyclable materials; characterization of the morphological surface of the MEDUn; interaction between water molecules and the surface of the MEDUn surface; electrochemical parameters of the MEDPUn and MEDAT (PDF)

## References

[ref1] de Souza W. M.; Buss L. F.; Candido D. d. S.; Carrera J. P.; Li S.; Zarebski A. E.; Pereira R. H. M.; Prete C. A. Jr.; de Souza-Santos A. A.; Parag K. V.; Belotti M. C. T. D.; Vincenti-Gonzalez M. F.; Messina J.; da Silva Sales F. C.; Andrade P. d. S.; Nascimento V. H.; Ghilardi F.; Abade L.; Gutierrez B.; Kraemer M. U. G.; Braga C. K. V.; Aguiar R. S.; Alexander N.; Mayaud P.; Brady O. J.; Marcilio I.; Gouveia N.; Li G.; Tami A.; de Oliveira S. B.; Porto V. B. G.; Ganem F.; de Almeida W. A. F.; Fantinato F. F. S. T.; Macário E. M.; de Oliveira W. K.; Nogueira M. L.; Pybus O. G.; Wu C. H.; Croda J.; Sabino E. C.; Faria N. R. Epidemiological and clinical characteristics of the COVID-19 epidemic in Brazil. Nat. Human Behav. 2020, 4, 856–865. 10.1038/s41562-020-0928-4. 32737472

[ref2] Okell L. C.; Verity R.; Watson O. J.; Mishra S.; Walker P.; Whittaker C.; Katzourakis A.; Donnelly C. A.; Riley S.; Ghani A. C.; Gandy A.; Flaxman S.; Ferguson N. N.; Bhatt S. Have deaths from COVID-19 in Europe plateaued due to herd immunity?. Lancet 2020, 395, e110–e111. 10.1016/S0140-6736(20)31357-X. 32534627 PMC7289569

[ref3] Sabino E. C.; Buss L. F.; Carvalho M. P. S.; Prete C. A. Jr.; Crispim M. A. E.; Fraiji N. A.; Pereira R. H. M.; Parag K. V.; da Silva Peixoto P.; Kraemer M. U. G.; Oikawa M. K.; Salomon T.; Cucunuba Z. M.; Castro M. C.; de Souza Santos A. A.; Nascimento V. H.; Pereira H. S.; Ferguson N. M.; Pybus O. G.; Kucharski A.; Busch M. P.; Dye C.; Faria N. R. Resurgence of COVID-19 in Manaus, Brazil, despite high seroprevalence. Lancet 2021, 397, 452–455. 10.1016/S0140-6736(21)00183-5. 33515491 PMC7906746

[ref4] Buss L. F.; Prete C. A. Jr.; Abrahim C. M. M.; Mendrone A.; Salomon T.; de Almeida-Neto C.; França R. F. O.; Belotti M. C.; Carvalho M. P. S. S.; Costa A. G.; Crispim M. A. E.; Ferreira S. C.; Fraiji N. A.; Gurzenda S.; Whittaker C.; Kamaura L. T.; Takecian P. L.; da Silva Peixoto P.; Oikawa M. K.; Nishiya A. S.; Rocha V.; Salles N. A.; de Souza Santos A. A.; da Silva M. A.; Custer B.; Parag K. V.; Barral-Netto M.; Kraemer M. U. G.; Pereira R. H. M.; Pybus O. G.; Busch M. P.; Castro M. C.; Dye C.; Nascimento V. H.; Faria N. R.; Sabino E. C. Three-quarters attack rate of SARS-CoV-2 in the Brazilian Amazon during a largely unmitigated epidemic. Science 2021, 371, 288–292. 10.1126/science.abe9728. 33293339 PMC7857406

[ref5] Fabiani L.; Saroglia M.; Galatà G.; De Santis R.; Fillo S.; Luca V.; Faggioni G.; D'Amore N.; Regalbuto E.; Salvatori P.; Terova G.; Moscone D.; Lista F.; Arduini F. Magnetic beads combined with carbon black-based screen-printed electrodes for COVID-19: A reliable and miniaturized electrochemical immunosensor for SARS-CoV-2 detection in saliva. Biosens. Bioelectron. 2021, 171, 112686 10.1016/j.bios.2020.112686. 33086175 PMC7833515

[ref6] Mattioli I. A.; Hassan A.; Oliveira O. N. Jr.; Crespilho F. N. On the challenges for the diagnosis of SARS-CoV-2 based on a review of current methodologies. ACS Sens. 2020, 5, 3655–3677. 10.1021/acssensors.0c01382. 33267587

[ref7] Sigley E.; Kalinke C.; Crapnell R. D.; Whittingham M. J.; Williams R. J.; Keefe E. M.; Janegitz B. C.; Bonacin J. A.; Banks C. E. Circular economy electrochemistry: creating additive manufacturing feedstocks for caffeine detection from post-industrial coffee pod waste. ACS Sustain Chem. Eng. 2023, 11, 2978–88. 10.1021/acssuschemeng.2c06514. 36844748 PMC9945317

[ref8] Crapnell R. D.; Sigley E.; Williams R. J.; Brine T.; Ferrari A. G-M.; Kalinke C.; Janegitz B. C.; Bonacin J. A.; Banks C. E. Circular economy electrochemistry: recycling old mixed material additively manufactured sensors into new electroanalytical sensing platforms. ACS Sustainable Chem. Eng. 2023, 11 (24), 9183–9193. 10.1021/acssuschemeng.3c02052. 37351461 PMC10284352

[ref9] Huang J.; Boles S. T.; Tarascon J.-M. Sensing as the key to battery lifetime and sustainability. Nature Sustainability 2022, 5, 194–204. 10.1038/s41893-022-00859-y.

[ref10] Bachmann M.; Zibunas C.; Hartmann J.; Tulus V.; Suh S.; Guillén-Gosálbez G.; Bardow A. Towards circular plastics within planetary boundaries. Nature Sustainability 2023, 6, 599–610. 10.1038/s41893-022-01054-9.

[ref11] Vidal F.; van der Marel E. R.; Kerr R. W. F.; McElroy C.; Schroeder N.; Mitchell C.; Rosetto G.; Chen T. T. D.; Bailey R. M.; Hepburn C.; Redgwell C.; Williams C. K. Designing a circular carbon and plastics economy for a sustainable future. Nature 2024, 626, 45–57. 10.1038/s41586-023-06939-z. 38297170

[ref12] Zisopoulos F. K.; Fath B. D.; Toboso-Chavero S.; Huang H.; Schraven D.; Steuer B.; Stefanakis A.; Clark O. G.; Scrieciu S.; Singh S.; Noll D.; de Jong M. Inequities blocking the path to circular economies: A bio-inspired network-based approach for assessing the sustainability of the global trade of waste metals. Resour., Conserv. Recycl. 2025, 212, 107958 10.1016/j.resconrec.2024.107958.

[ref13] Crapnell R. D.; Banks C. E. Electroanalysis overview: addressing the green credentials in the use of electroanalytical sensors. Green Carbon 2023, 1, 85–93. 10.1016/j.greenca.2023.09.003.

[ref14] Kalinke C.; Crapnell R. D.; de Oliveira P. R.; Janegitz B. C.; Bonacin J. A.; Banks C. E. How to Improve Sustainability in Fused Filament Fabrication (3D Printing) Research?. Global Challenges 2024, 8, 2300408 10.1002/gch2.202300408. 39006060 PMC11237179

[ref15] Sharma A.; Faber H.; Khosla A.; Anthopoulos T. D. 3D printed electrochemical devices for bio-chemical sensing: a review. Mater. Sci. Eng., R 2023, 156, 100754 10.1016/j.mser.2023.100754.

[ref16] Chaibun T.; Puenpa J.; Ngamdee T.; Boonapatcharoen N.; Athamanolap P.; O’Mullane A. P.; Vongpunsawad S.; Poovorawan Y.; Lee S. Y.; Lertanantawong B. Rapid electrochemical detection of coronavirus SARS-CoV-2. Nat. Commun. 2021, 12, 802 10.1038/s41467-021-21121-7. 33547323 PMC7864991

[ref17] Pradela-filho L. A.; Andreotti I. A. A.; Carvalho J. H. S.; Araújo D. A. G.; Orzari L. O.; Gatti A.; Takeuchi R. M.; Santos A. L.; Janegitz B. C. Glass varnish-based carbon conductive ink: A new way to produce disposable electrochemical sensors. Sens. Actuators, B 2020, 305, 127433 10.1016/j.snb.2019.127433.

[ref18] Marchianò V.; Tricase A.; Caputo M.; Farinini E.; Leardi R.; Imbriano A.; Leech D.; Kidayaveettil R.; Gentile L.; Torsi L.; Macchia E.; Bollella P. Tailoring Water-Based Graphite Conductive Ink Formulation for Enzyme Stencil-Printing: Experimental Design to Enhance Wearable Biosensor Performance. Chem. Mater. 2024, 36, 358–370. 10.1021/acs.chemmater.3c02229.

[ref19] Henrique J. M.; Camargo J. R.; de Oliveira G. G.; Stefano J. S.; Janegitz B. C. Disposable electrochemical sensor based on shellac and graphite for sulfamethoxazole detection. Microchem. J. 2021, 170, 106701 10.1016/j.microc.2021.106701.

[ref20] Silva M. K.; Sousa G. S.; Simoes R. P.; Cesarino I. Fabrication of paper-based analytical devices using a PLA 3D-printed stencil for electrochemical determination of chloroquine and escitalopram. J. Solid State Electrochem. 2022, 26 (2), 581–586. 10.1007/s10008-021-05075-w. 34751209 PMC8566020

[ref21] Dreimol C. H.; Guo H.; Ritter M.; Keplinger T.; Ding Y.; Günther R.; Poloni E.; Burger I.; Panzarasa G. Sustainable wood electronics by iron-catalyzed laser-induced graphitization for large-scale applications. Nat. Commun. 2022, 13, 3680 10.1038/s41467-022-31283-7. 35760793 PMC9237073

[ref22] Vadivel S.; Tejangkura W.; Sawangphruk M. Graphite/graphene composites from the recovered spent Zn/carbon primary cell for the high-performance anode of lithium-ion batteries. ACS omega 2020, 5, 15240–15246. 10.1021/acsomega.0c01270. 32637797 PMC7331064

[ref23] Pimentel G. J. C.; Ayres L. B.; Costa J. N. Y.; Paschoalino W. J.; Whitehead K.; Kubota L. T.; de Oliveira Piazzetta M. H.; Gobbi A. L.; Shimizu F. M.; Garcia C. D.; Lima R. S. Ultradense Electrochemical Chips with Arrays of Nanostructured Microelectrodes to Enable Sensitive Diffusion-Limited Bioassays ACS Appl. Mater. Interfaces 2024 10.1021/acsami.4c01159. 38537173

[ref24] Microelectrodes to Enable Sensitive Diffusion-Limited Bioassays. ACS Appl. Mater. Interfaces 2024, 27. 10.1021/acsami.4c01159 38537173

[ref25] Torrente-Rodríguez R. M.; Lukas H.; Tu J.; Min J.; Yang Y.; Xu C.; Rossiter H. B.; Gao W. SARS-CoV-2 RapidPlex: a graphene-based multiplexed telemedicine platform for rapid and low-cost COVID-19 diagnosis and monitoring. Matter 2020, 3, 1981–1998. 10.1016/j.matt.2020.09.027. 33043291 PMC7535803

[ref26] Wrobel A. G.; Benton D. J.; Hussain S.; Harvey R.; Martin S. R.; Roustan C.; Rosenthal P. B.; Skehel J. J.; Gamblin S. J. Antibody-mediated disruption of the SARS-CoV-2 spike glycoprotein. Nat. Commun. 2020, 11, 5337 10.1038/s41467-020-19146-5. 33087721 PMC7577971

[ref27] Yuan M.; Wu N. C.; Zhu X.; Lee C-C. D.; So R. T. Y.; Lv H.; Mok C. K. P.; Wilson I. A. A highly conserved cryptic epitope in the receptor binding domains of SARS-CoV-2 and SARS-CoV. Science 2020, 368, 630–633. 10.1126/science.abb7269. 32245784 PMC7164391

