# Supplementary material for: Sustainable Electrochemical-Magnetic Biosensor Fabricated from Recycled Materials for Label-Free Detection of SARS-CoV-2 in Human Saliva
Source: ACS Sens. 2025 Mar 14;10(3):1970–85. doi: 10.1021/acssensors.4c03175 (PMC11959605; doi:10.1021/acssensors.4c03175)
Supplement: Supplementary file 1 — se4c03175_si_001.pdf [file se4c03175_si_001.pdf]

Supplementary information

Sustainable Electrochemical-Magnetic Biosensor Fabricated from Recycled Materials for Label-free Detection of SARS-CoV-2 in Human Saliva

Caio L. C. Carvalho[a], Steffane Q. Nascimento[a], Thiago Bertaglia[a], Luana C. I. Faria[a], Erika R. Manuli[b,c], Geovana M. Pereira[b,c], Welter Cantanhêde[d], Carlos M. Costa[e,f], Josu Fernández Maestu[g], Senentxu Lanceros-Méndez[e,f,g,h], Osvaldo Novais Oliveira Jr.[i], Ester C. Sabino[b,c], Frank N. Crespilho[a,e,g,].*

[a]*São Carlos Institute of Chemistry, University of São Paulo (USP), 13560-970, São Carlos, Brazil.*

[b]*Institute of Tropical Medicine, Faculty of Medicine, University of São Paulo, São Paulo, SP, 05403-000, Brazil.*

[c]*LIM-46 HC-FMUSP – Laboratory of Medical Investigation, Clinical Hospital, Faculty of Medicine, University of São Paulo, São Paulo, SP, 01246903, Brazil.*

[d]*Supramolecular Self-Assembly Laboratory, Department of Chemistry, Federal University of Piauí, 64049-550 Teresina, PI, Brazil.*

[e]*Physics Centre of Minho and Porto Universities (CF-UM-UP) and Laboratory of Physics for Materials and Emergent Technologies, LapMET, University of Minho, 4710-057 Braga, Portugal.*

[f]*Institute of Science and Innovation for Bio-Sustainability (IB-S), University of Minho, 4710-053, Braga, Portugal.*

[g]*BCMaterials, Basque Center for Materials, Applications and Nanostructures, UPV/EHU Science Park, 48940 Leioa, Spain.*

[h]*IKERBASQUE, Basque Foundation for Science, Bilbao, 48009, Spain.*

[i]*Sao Carlos Institute of Physics, University of São Paulo, São Carlos, Brazil.*

[*]Correspondence authors:

E-mail addresses: frankcrespilho@iqsc.usp.br (Frank N. Crespilho)

Table of Contents

**Fig. S1** Infographic of the materials used for fabrication of thousand MED and the estimated price per device.	4
**Fig. S2** Illustration of the strategy used to select recyclable materials to calculate the recyclability rate using Equation 3.	5
**Fig. S3** Characterization of the morphological surface of the MEDUn by scanning electron microscopy (SEM).	6
**Fig. S4** Interaction between water molecules and the surface of the MEDUn surface.	7
**Fig. S5** Evaluation of the electrochemical parameters of the MEDPUn and MEDAT.	8
**Fig. S6** Study of electron transfer resistance for MEDUn.	9
**Fig. S7** Optimization of the amount of graphite carbon and polystyrene in the MEDAT.	10
**Fig. S8** Evaluation of the electrochemical parameters for different compositions (%) of carbon.	11
**Fig. S9** Behavior of transfer resistance for electrodes with different carbon composition.	12
**Fig. S10** Results of charge-transfer resistance (Rct) for different compositions (%) of carbon.	13
**Fig. S11** Raman spectra of MEDPUn and MEDAT from 3500 to 100 cm^{-1}.	14
**Fig. S12** Deconvolution data of Raman spectra of the bands D, G and D´ for MEDPUn and MEDAT.	15
**Fig. S13** Investigation of the electron transfer mechanism of $[Fe^{III}(CN)_6]^{3-}/[Fe^{II}(CN)_6]^{4-}$ onto MEDAT surface.	16
**Fig. S14** Study of the electrochemical reversibility criteria of $[Fe^{III}(CN)_6]^{3-}/[Fe^{II}(CN)_6]^{4-}$ onto MEDAT surface.	17
**Fig. S15** Mass production capability test of the MEDAT.	18
**Fig. S16** Plots of pH *versus* Epa for MEDAT.	19
**Fig. S17** Plots of jpa *versus* pH for MEDAT.	20
**Fig. S18** Crystal structure analysis of the $MnFe_2O_4$-cys NPs.	21
**Fig. S19** Morphological characterization of the $MnFe_2O_4$-cys NPs.	22
**Fig. S20** Structural organization and distribution of nanoparticles of the bioconjugate.	23
**Fig. S21** Morphology of $MnFe_2O_4$ NPs modified with EDC and NHS.	24
**Fig. S22** Microanalysis of the chemical composition of the $MnFe_2O_4$ NPs modified with EDC and NHS.	25
**Fig. S23** Chemical micro-mapping of the $MnFe_2O_4$ NPs modified with EDC and NHS.	26
**Fig. S24** Fourier transform infrared spectroscopy (FTIR) for functionalized with cysteine.	27
**Fig. S25** Chemical images of the bioconjugate.	28
**Fig. S26** Investigation of magnetic properties on the electrode surface with an integrated magnet.	29
**Fig. S27** Structural organization and distribution of $MnFe_2O_4$ NPs onto MED surface.	30
**Fig. S28** Microanalysis of the chemical composition for electrode modified with $MnFe_2O_4$ NPs.	31
**Fig. S29** Morphology surface of the MED modified with bioconjugate.	32
**Fig. S30** Morphology surface of the MED modified with $MnFe_2O_4$-EDC:NHS|MED.	33
**Fig. S31** Structural organization and distribution of bioconjugate onto MED surface.	34
**Fig. S32** Microanalysis of the chemical composition for MED modified with $MnFe_2O_4$-EDC:NHS.	35
**Fig. S33** Chemical composition for MED modified with bioconjugate.	36
**Fig. S34** Chemical mapping of the MED modified with $MnFe_2O_4$-EDC:NHS nanoparticles.	37
**Fig. S35** Chemical micro-mapping of the MED modified with $MnFe_2O_4$-EDC:NHS nanoparticles and bioconjugate.	38
**Fig. S36** Study of the optimization experimental conditions.	39
**Fig. S37** Optimization of immunoreaction time.	40
**Fig. S38** Electrochemical behavior of the same biosensor for magnetic detection immunoassays.	41
**Fig. S39** Profile of current densities of storage stability study for different biosensors.	42
**Fig. S40** Electrochemical behavior of the biosensor for recovery assays in saliva samples.	43
**Table S1** Experimental data to estimate the recyclability of the spent Zn/C batteries.	44
**Table S2** Comparison of the MED with some articles that used circular economy concept for preparation of electrochemical devices.	45
**Table S3** Values of the main studied magnetic parameters of the investigated nanomaterials.	46
**Table S4** Electroanalytical performance of the developed electrochemical-magnetic biosensor.	47
**Table S5** Details of data obtained in recovery tests.	48
**Table S6** Main parameters obtained in the ROC curve construction procedure.	49
**Table S7** Average reactivity index values for the saliva samples analyzed.	50
**Table S8** Main clinical validation criteria for the proposed biosensor.	51
**Table S9** Analysis of the economic viability of the biosensor.	52
**References**	53

Materials and methods

Chemicals and Materials

All chemical products were of analytical grade and used without any type of purification. Manganese(II) chloride tetrahydrate ($MnCl_2.4H_2O$, ≥ 99%), iron(III) chloride hexahydrate ($FeCl_3.6H_2O$, ≥ 99%), L-cysteine (Cys, $C_3H_7NO_2S$, 97%), and sodium hydroxide (NaOH, ≥ 98%) were acquired from Sigma-Aldrich® (St Louis, MO, USA). N-(3-Dimethylaminopropyl)-N′-ethylcarbodiimide hydrochloride (EDC, ≥ 98%), N-hydroxysuccinimide (NHS, 98%), bovine serum albumin (BSA, ≥ 98%), and 2-(N-morpholino) ethanesulfonic acid (MES, ≥ 99.5%) were purchased from Sigma-Aldrich®. Recombinant anti-SARS-CoV-2 Spike Glycoprotein S1 antibody CR3022 (S1-Ab) was obtained from Abcam® (Cambridge, MA, USA). SARS-CoV-2 Spike Protein S1 Receptor-Binding Domain (S1-RBD) was acquired from Biolinker® (São Paulo, Brazil). Monobasic potassium phosphate (99%, KH_2PO_4), sodium phosphate dibasic (99%, Na_2HPO_4), potassium chloride (99%, KCl) and sodium chloride (99%, NaCl) were supplied from Dinâmica® (Indaiatuba, SP, Brazil). Chloroform (99.8 %, CH_3Cl), isopropyl alcohol (70%, C_3H_7O), and ethanol (99.5%, C_2H_6O) were acquired from Dinâmica®. Potassium hexacyanoferrate(II) trihydrate ($K_4[Fe(CN)_6].3H_2O$, ≥98.5%), and potassium hexacyanoferrate(III) ($K_3[Fe(CN)_6]$, ≥99%) were obtained from Sigma-Aldrich®. All solutions were prepared with ultrapure water (resistivity of 18.2 MΩ cm at 25ºC) purified by a Millipore Direct-Q®3 UV system.

Synthesis of $MnFe_2O_4$ Nanoparticles

Manganese ferrite ($MnFe_2O_4$) nanoparticles (NPs) have garnered significant interest due to their superparamagnetic properties and potential applications in biomedicine, particularly in biosensing and targeted drug delivery. Modification of these nanoparticles with biocompatible molecules such as L-cysteine (Cys) enhances their stability, dispersibility, and functionality. This study focuses on the synthesis, characterization, and bio-conjugation of $MnFe_2O_4$ nanoparticles functionalized with L-cysteine ($MnFe_2O_4$-cys NPs), emphasizing their application in developing a biosensor for detecting the SARS-CoV-2 spike protein receptor-binding domain (S1-RBD). The $MnFe_2O_4$-cys NPs were synthesized using a chemical co-

precipitation method. Initially, 0.02 mol (5.406 g) of $FeCl_3 \cdot 6H_2O$, 0.01 mol (1.979 g) of $MnCl_2 \cdot 4H_2O$, and 0.01 mol (1.2116 g) of L-cysteine were dissolved in 100 mL of ultrapure water. The mixture was sonicated for 10 minutes at room temperature. This solution was then purged with nitrogen gas at 80°C for 10 minutes. Subsequently, 50 mL of 2.4 mol L^{-1} NaOH (0.12 mol, 4.8 g) were added dropwise to the mixture under vigorous stirring at 80°C. The appearance of a black precipitate indicated the formation of $MnFe_2O_4$ nanoparticles. The suspension was stirred for an additional 90 minutes, after which the product was magnetically separated and washed with ultrapure water until the supernatant reached a neutral pH. The resulting $MnFe_2O_4$-cys NPs were dried in an oven at 60°C for 12 hours.

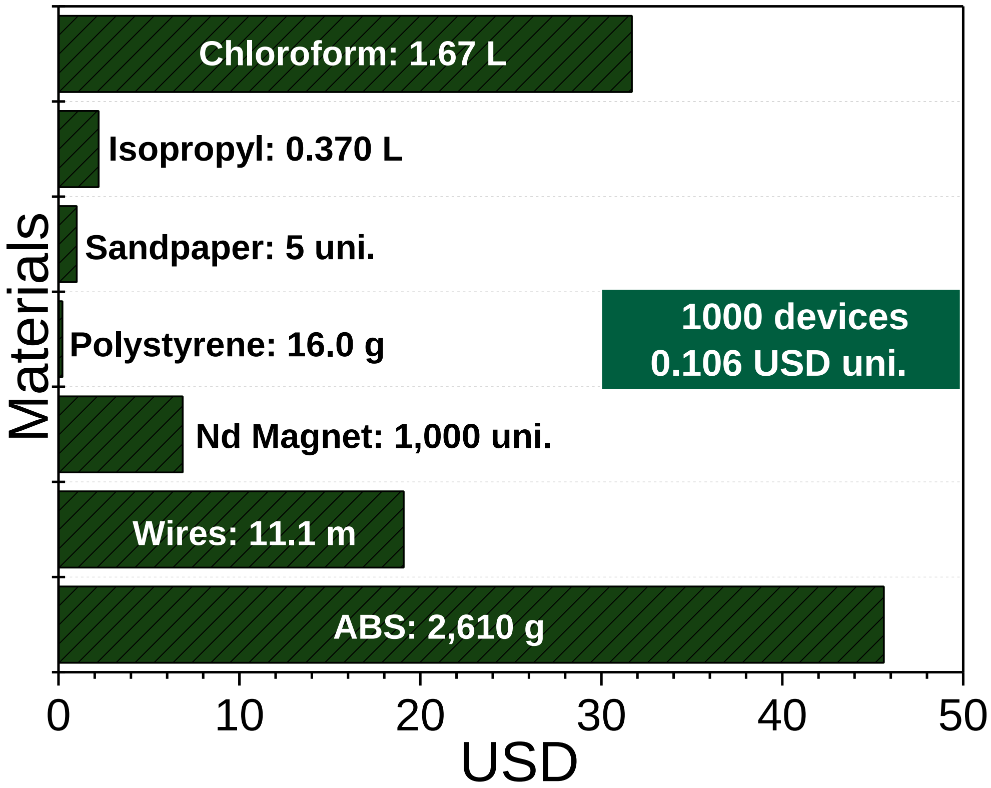

Fig. S1. Infographic of the materials used for fabrication of thousand MED and the estimated price per device. This figure is designed to estimate the cost of producing 1000 MED devices. It is important to highlight that we consider as zero cost the graphite carbon from of sepent Zn-C batteries (AA type). The batteries were collected from domestic and electronic waste in the city of São Carlos-SP/Brazil. Commercial disposable cup polystyrene was used to fabrication the devices after they were used. This means that before they are discarded in the trash, we reuse them. For this purpose, the glasses were washed with soap and water to completely remove beverage residues. This point demonstrates that we can use polystyrene cups that are discarded in trash.

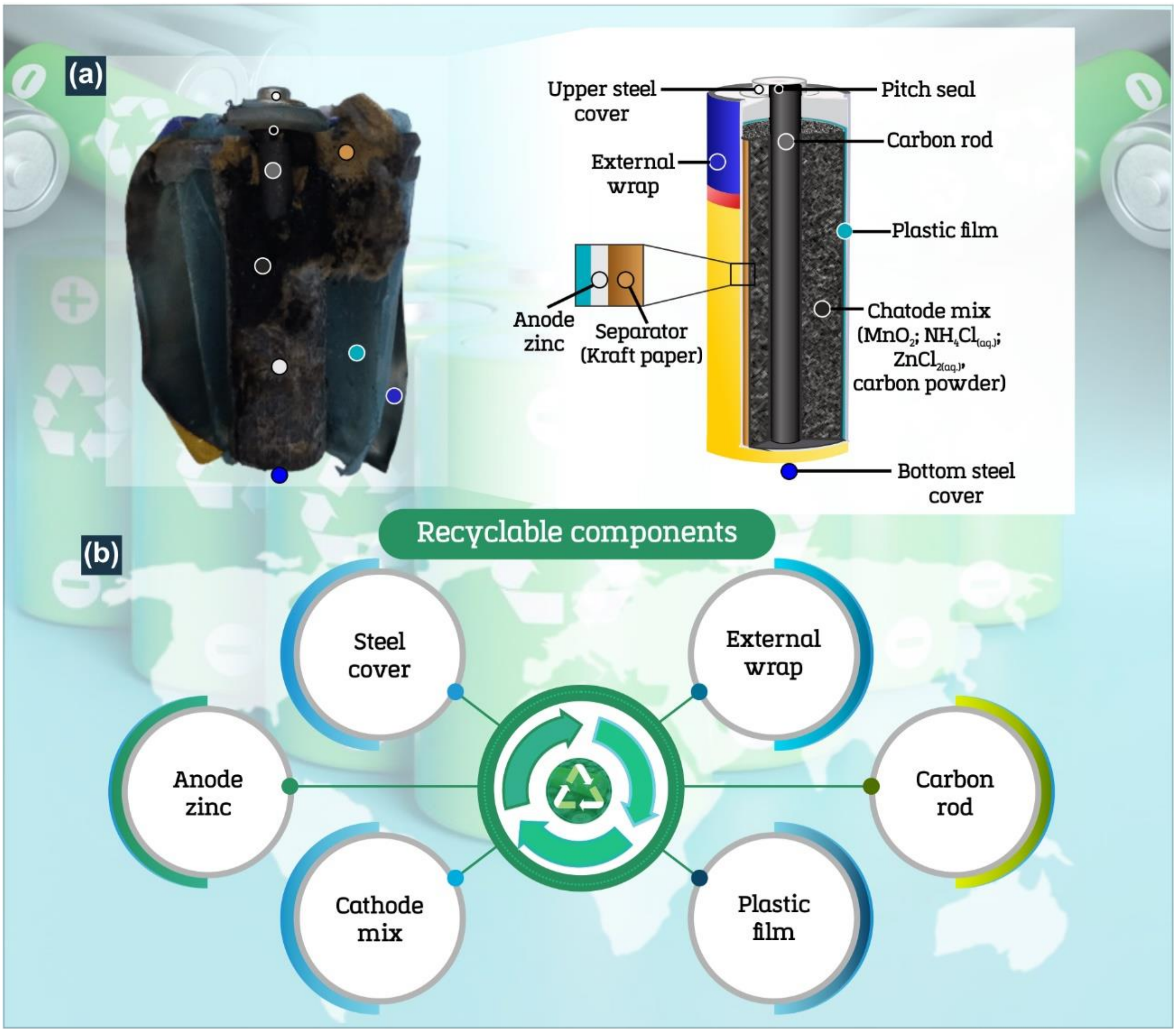

Fig. S2. Illustration of the strategy used to select recyclable materials to calculate the recyclability rate using Equation 3. (a) Photograph of spent Zn-C battery and illustration of its main components. (b) respective components that can be recovered or recycled. We carry out complete disassembly of a discharged Zn-C battery to check which components can be recovered or recycled.

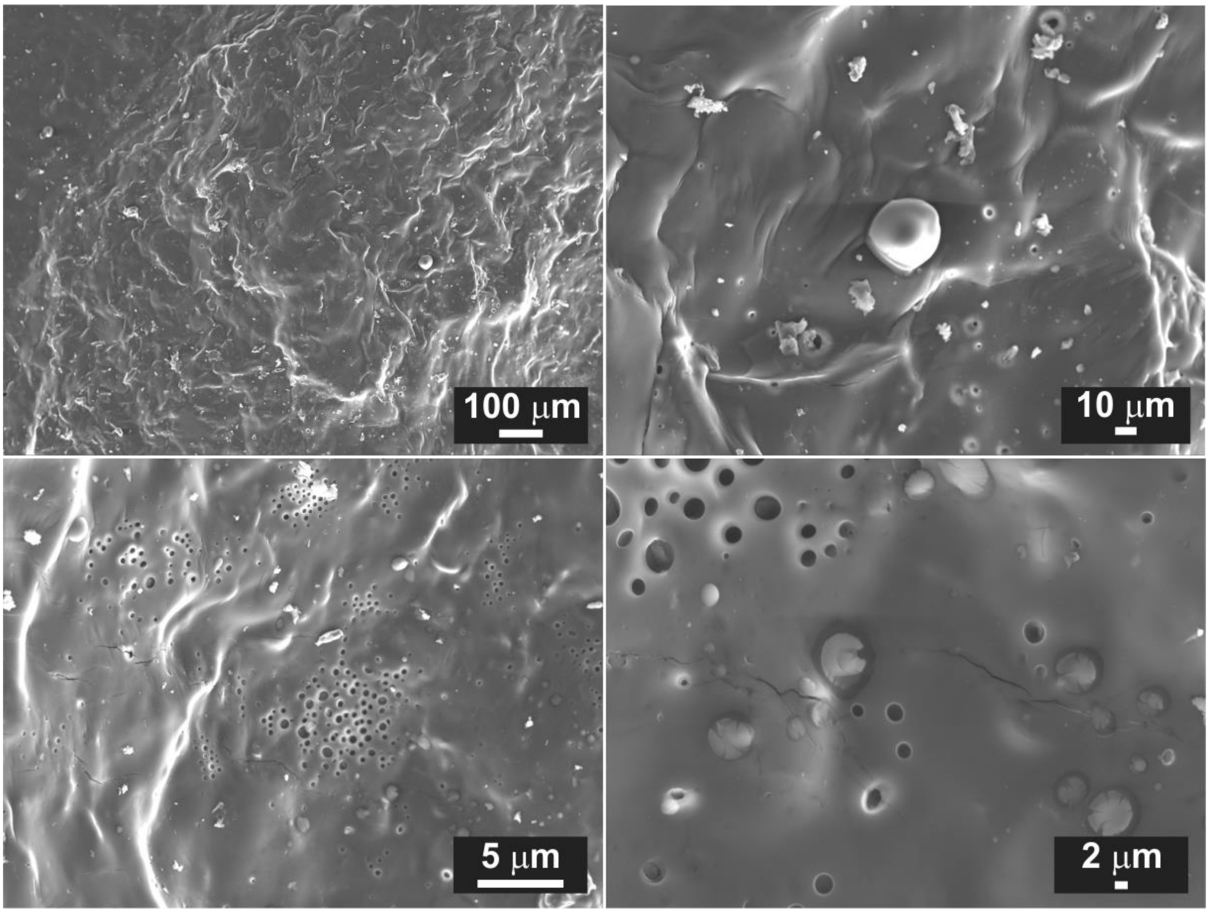

Fig. S3. Characterization of the morphological surface of the MEDUn by scanning electron microscopy (SEM). Conditions:15kV acceleration voltage and x200, x1000, x800 and x3,000 magnifications. In the micrograph, the pores, surface and spherical particles are characteristics of polystyrene from disposable cups.

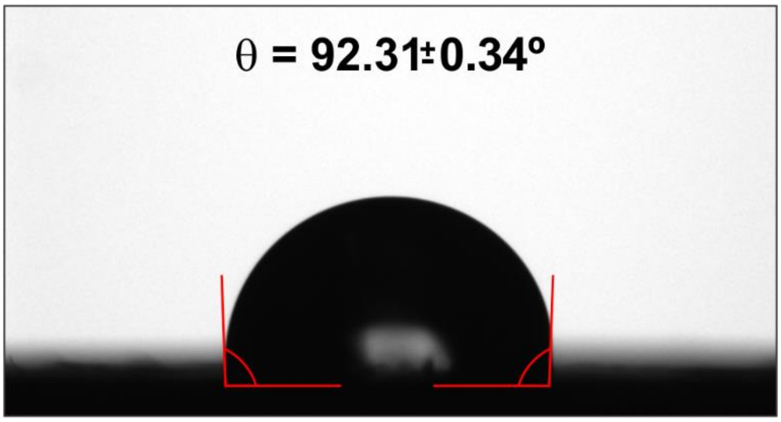

Fig. S4. Interaction between water molecules and the surface of the MEDUn surface. Estimating the contact angle is a method to evaluate the wettability of the electrode surface as well as its hydrophobic and hydrophilic character. For values greater than 90º the surface is classified as hydrophobic. While for values lower than 90º the surface has hydrophilic behavior.

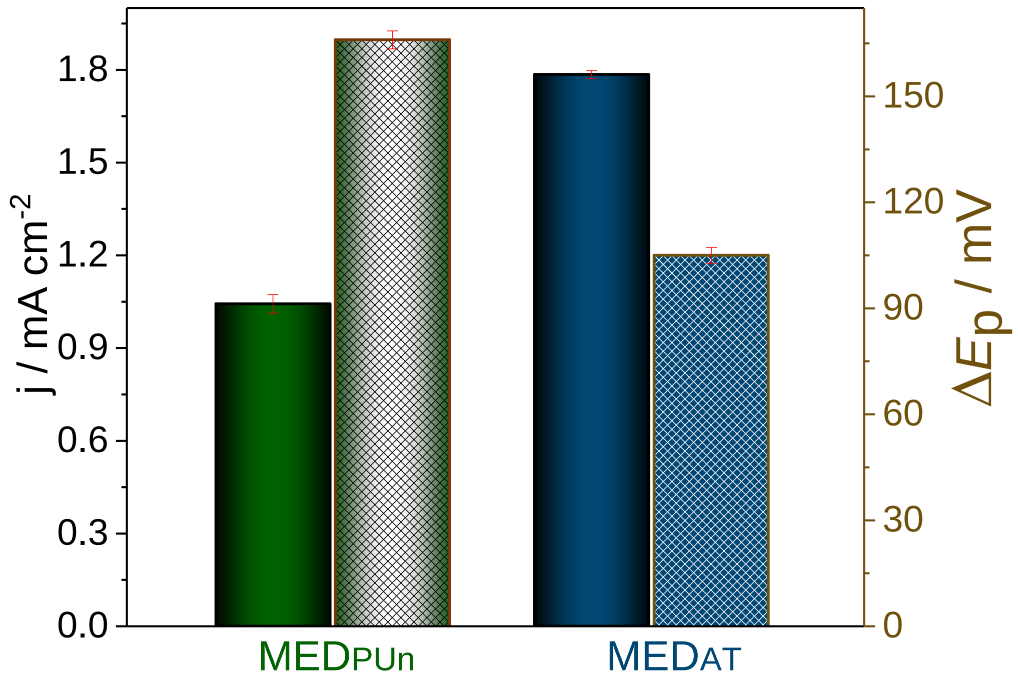

Fig. S5. Evaluation of the electrochemical parameters of the MEDPUn and MEDAT. Plots of jpa and ΔEp *v e r s u s* devices were crucial to define the best electrochemical performance of the electrodes in terms of reversibility and anode current density. 95% confidence intervals for all error bars (n = 3).

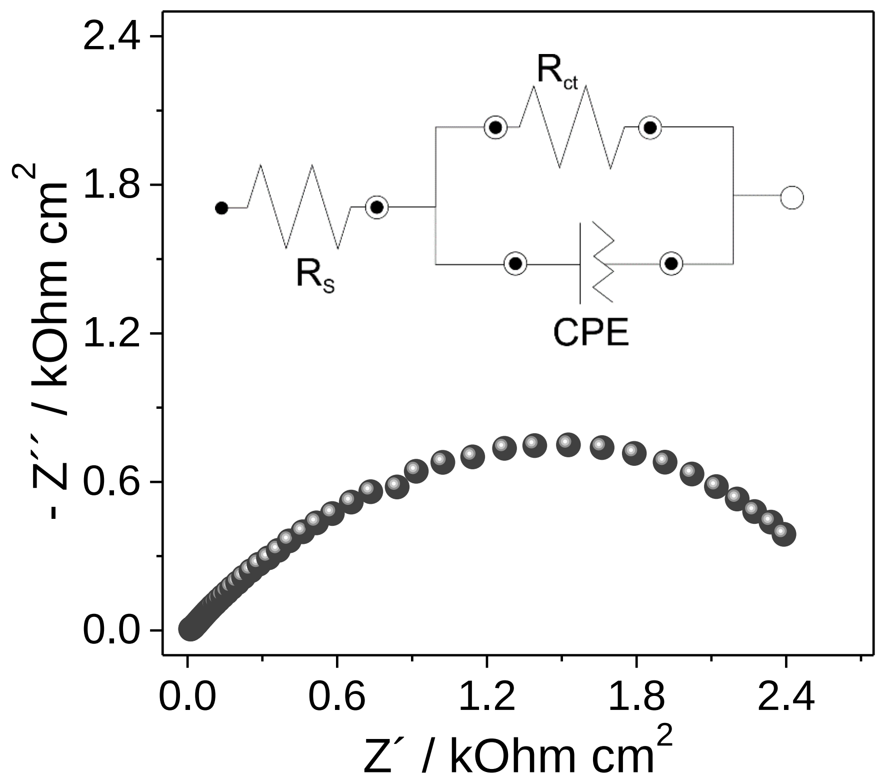

Fig. S6. Study of electron transfer resistance for MEDUn. EIS experiments were performed from 100 KHz to 0.1 Hz at 0.01 V for MEDUn. Inset showed equivalent circuit with absence of the element related to the redox process. The absence of the non-diffusion zone is consistent with the CV from Fig. S4.

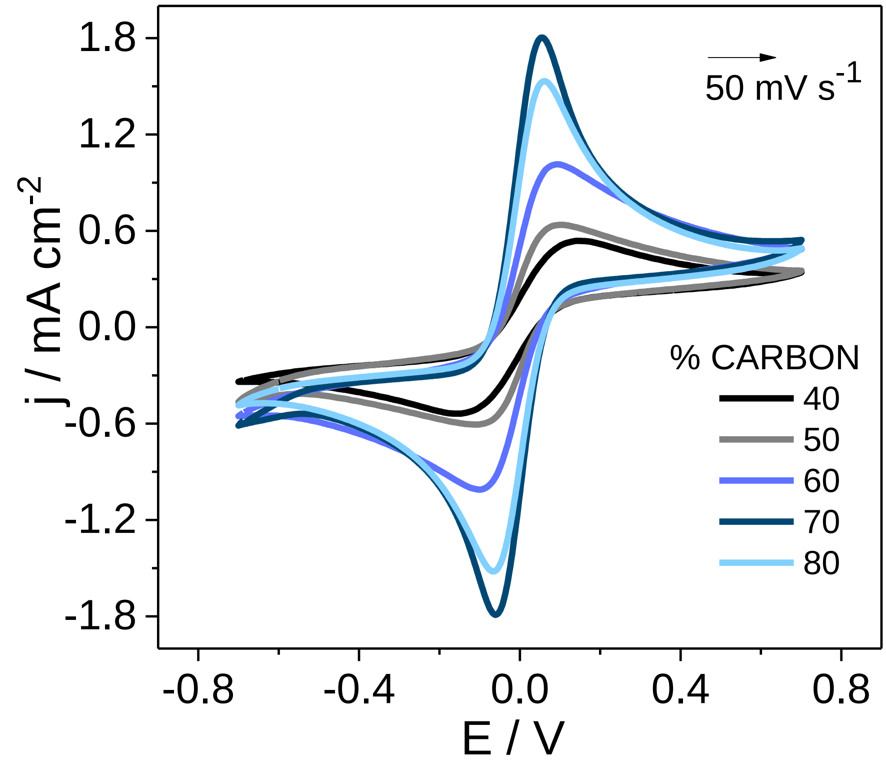

Fig. S7. Optimization of the amount of graphite carbon and polystyrene in the MEDAT. Cyclic voltammograms (CVs) for different compositions of carbon and polystyrene were obtained with 5.0 mmol L^{-1} $K_3[Fe(CN)_6]/K_4[Fe(CN)_6]$ (0.5 mol L^{-1} KCl), at 50 mV s^{-1}.

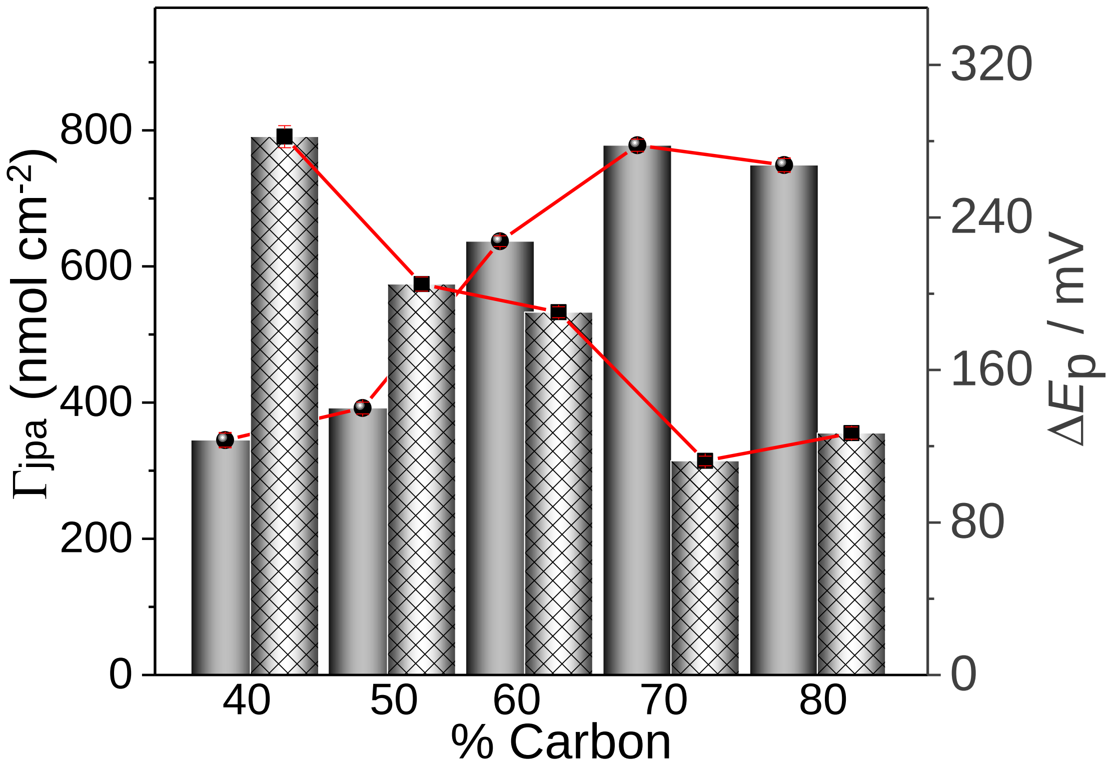

Fig. S8. Evaluation of the electrochemical parameters for different compositions (%) of carbon. The surface coverage (Γjpa) and ΔEp *versus* % carbon. This data were used to define the composition of the carbon ink that concentrates a greater amount of electroactive species and better electrochemical reversibility. 95% confidence intervals for all error bars (n = 3).

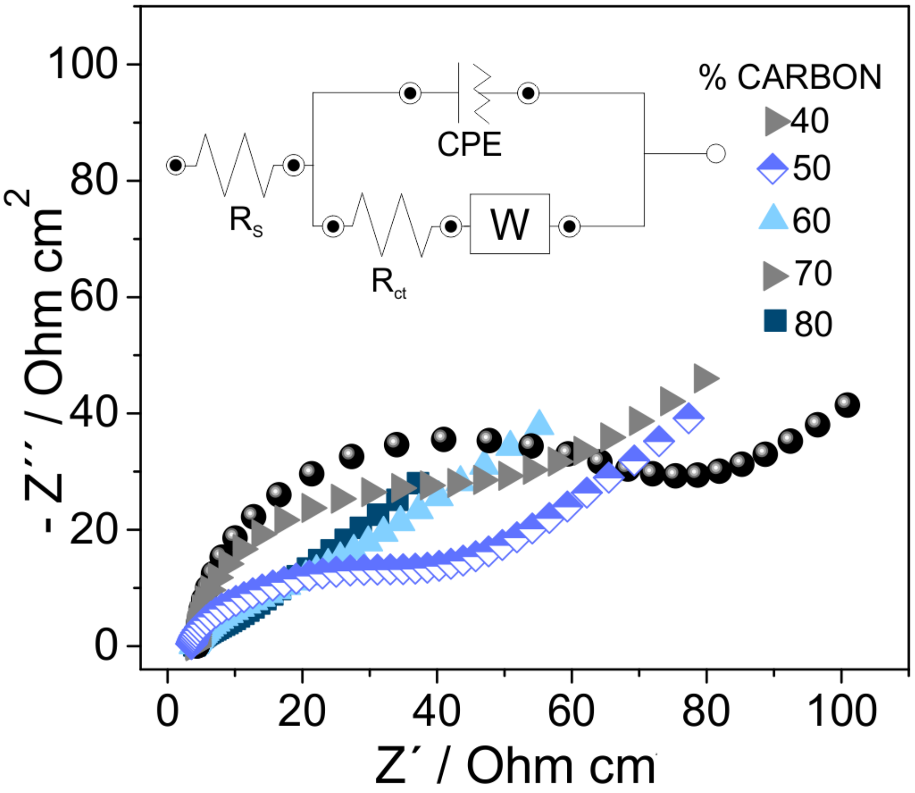

Fig. S9. Behavior of transfer resistance for electrodes with different carbon composition. EIS results were performed from 10000 to 0.1 Hz at 0.01 V.

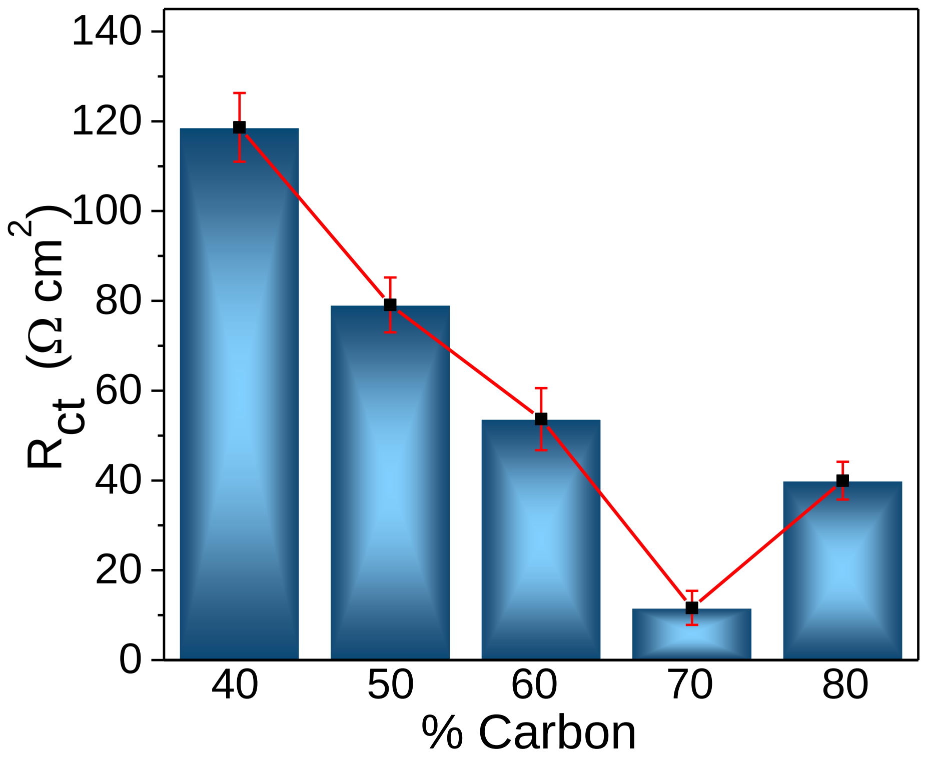

Fig. S10. Results of charge-transfer resistance (Rct) for different compositions (%) of carbon. . For this plot, the best proportion of carbon in the ink will be the system that exhibited the lowest Rct. 95% confidence intervals for all error bars (*n* = 3).

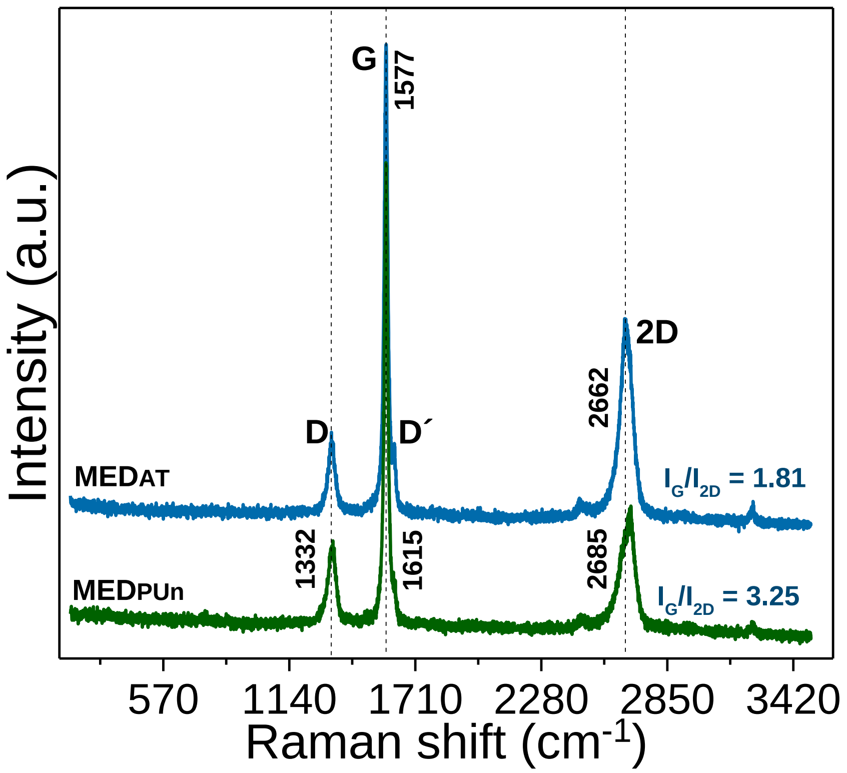

Fig. S11. Raman spectra of MEDPUn and MEDAT from 3500 to 100 cm^{-1}. The relationship between the intensity of the G and 2D (I_G/I_{2G}) bands was useful to evaluate the graphite multilayer behavior of the electrodes.

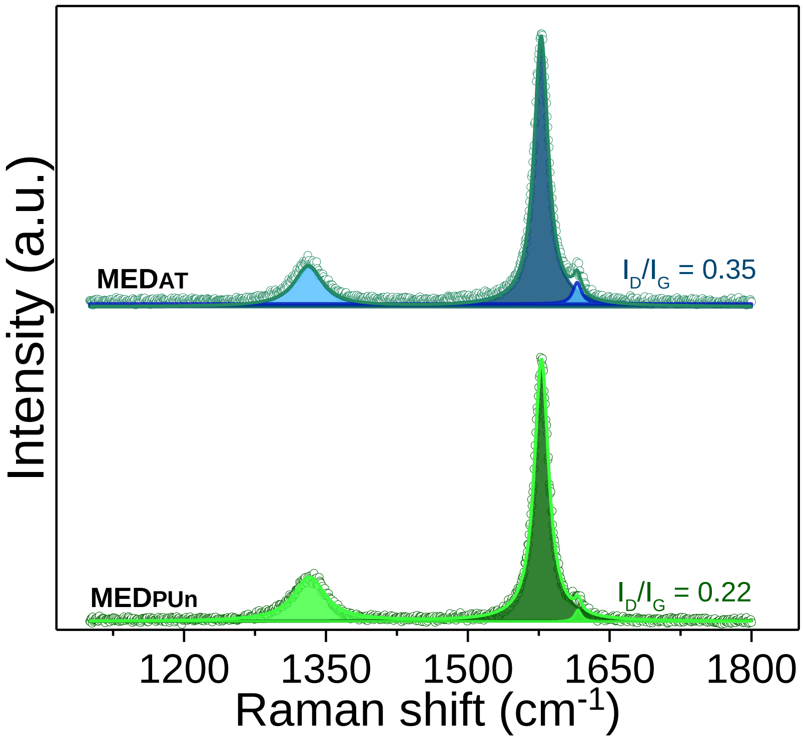

Fig. S12. Deconvolution data of Raman spectra of the bands D, G and D´ for MEDPUn and MEDAT. The relationship between the intensity of the D and G (I_D/I_G) bands was useful to analyze the degree of order of the graphite structures in the electrodes.

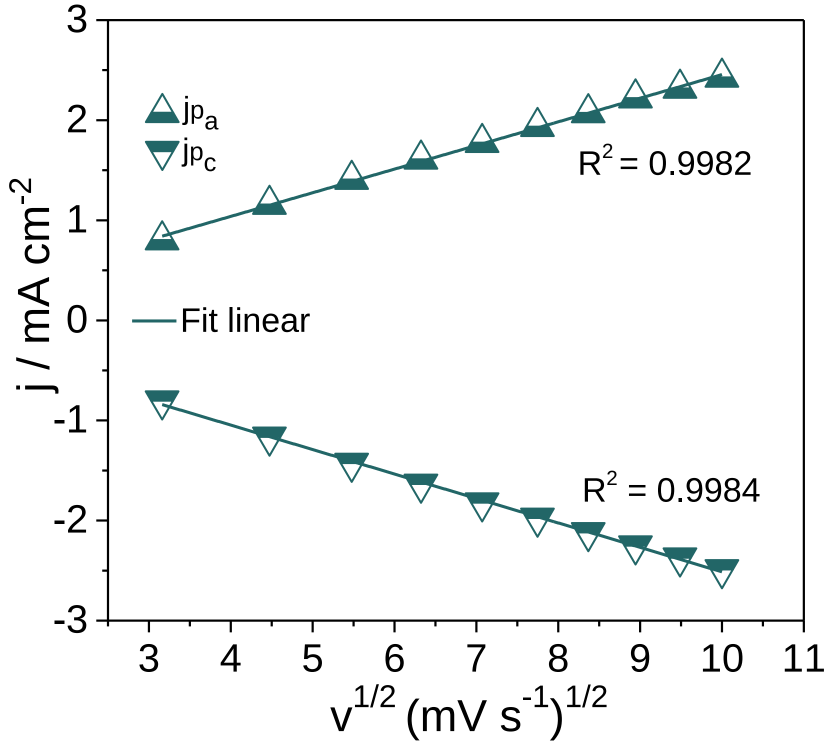

Fig. S13. Investigation of the electron transfer mechanism of $[Fe^{III}(CN)_6]^{3-}/[Fe^{II}(CN)_6]^{4-}$ onto MEDAT surface. The analysis of the linear behavior of jpa *versus* $v^{1/2}$ assists in identifying diffusion processes.

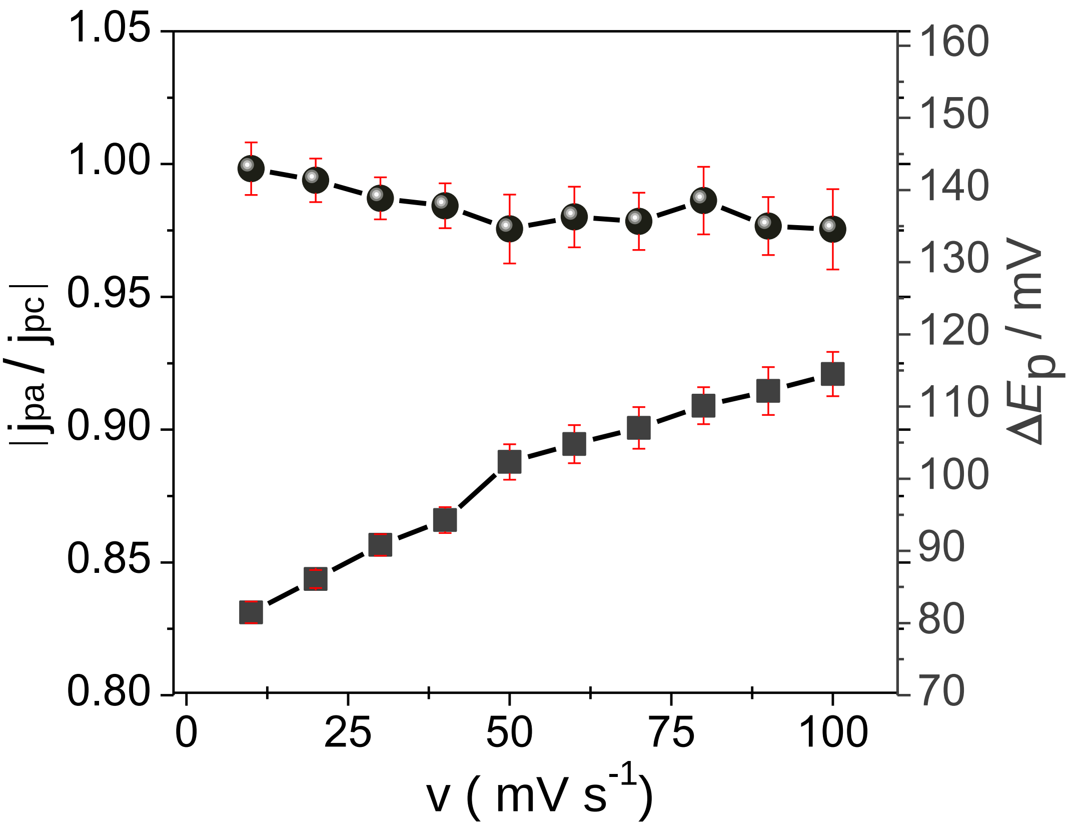

Fig. S14. Study of the electrochemical reversibility criteria of $[Fe^{III}(CN)_6]^{3-}/[Fe^{II}(CN)_6]^{4-}$ onto MEDAT surface. The analysis of the behavior of |jpa/jpc| and (ΔEp) *versus* scan rates is important to classify the reversibility of the $[Fe^{III}(CN)_6]^{3-}/[Fe^{II}(CN)_6]^{4-}$ redox couple. 95% confidence intervals for all error bars (n = 3).

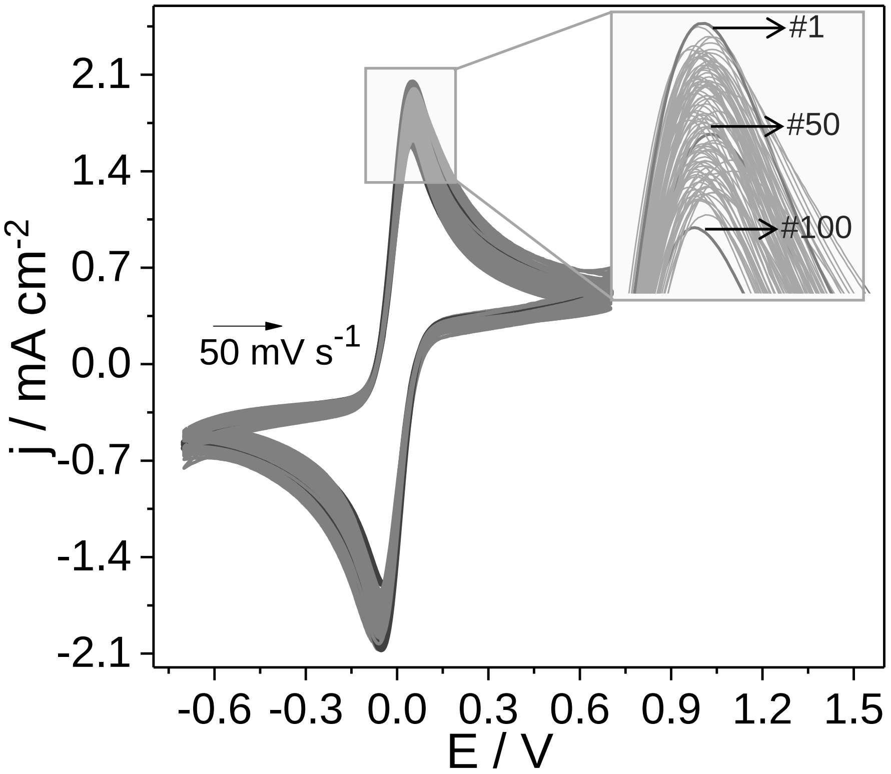

Fig. S15. Mass production capability test of the MEDAT. CVs of 100 MEDAT independently prepared under alternate days obtained in 5.0 mmol L^{-1} $K_3[Fe(CN)_6]/K_4[Fe(CN)_6]$ (0.5 mol L^{-1} KCl). Highlighting for electrochemical responses of the first, fiftieth and hundredth device manufactured.

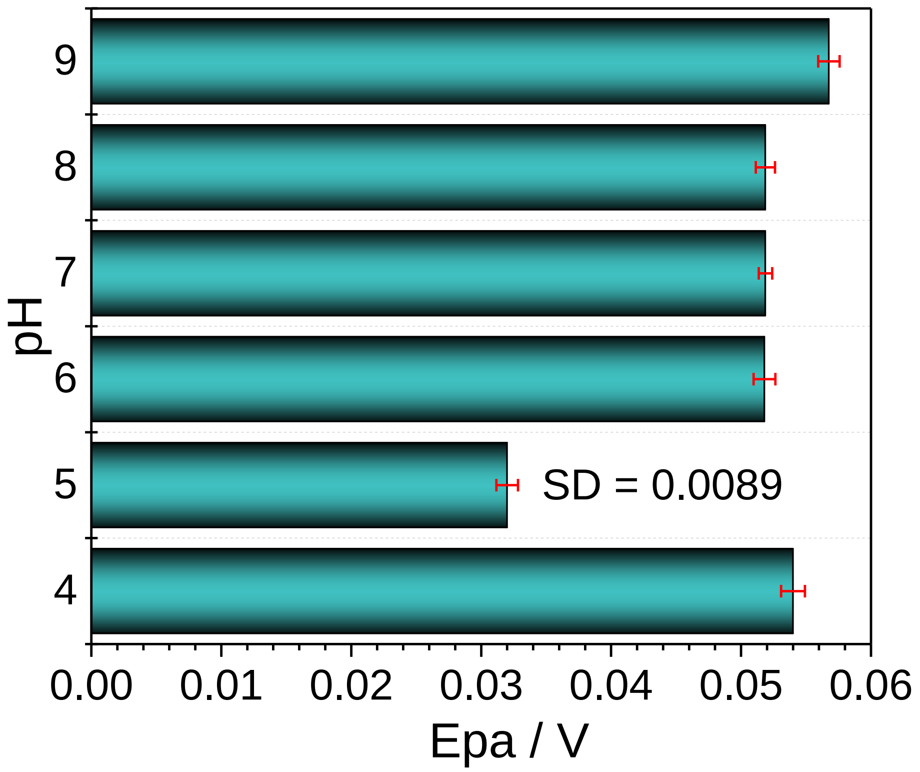

Fig. S16. Plots of pH *versus* Epa for MEDAT. The anodic peak potential values had a low standard deviation (SD). 95% confidence intervals for all error bars (n = 3).

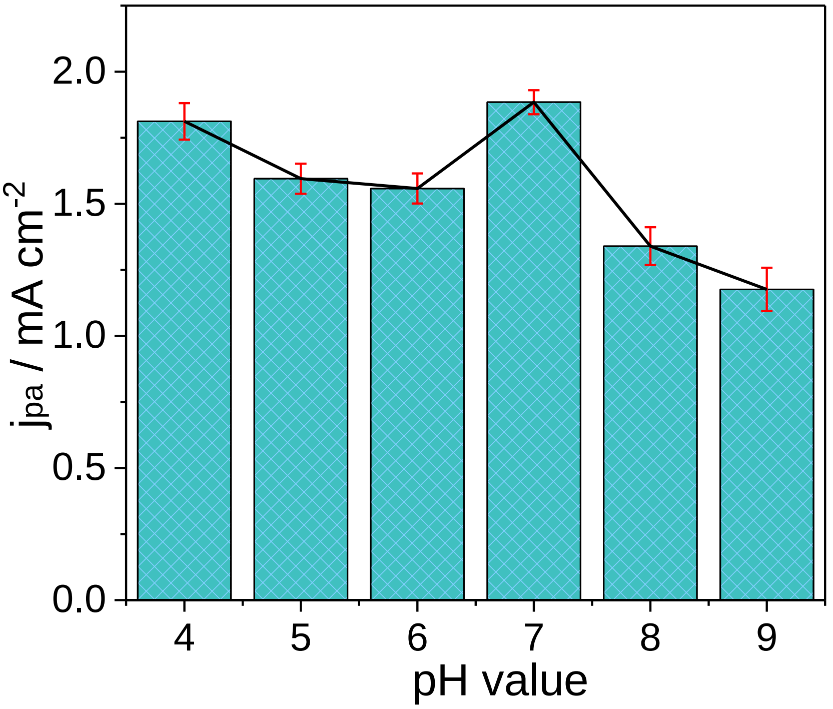

Fig. S17. Plots of jpa *versus* pH for MEDAT. The better performance in terms of anode current density was close to the pH of physiological systems. 95% confidence intervals for all error bars (n = 3).

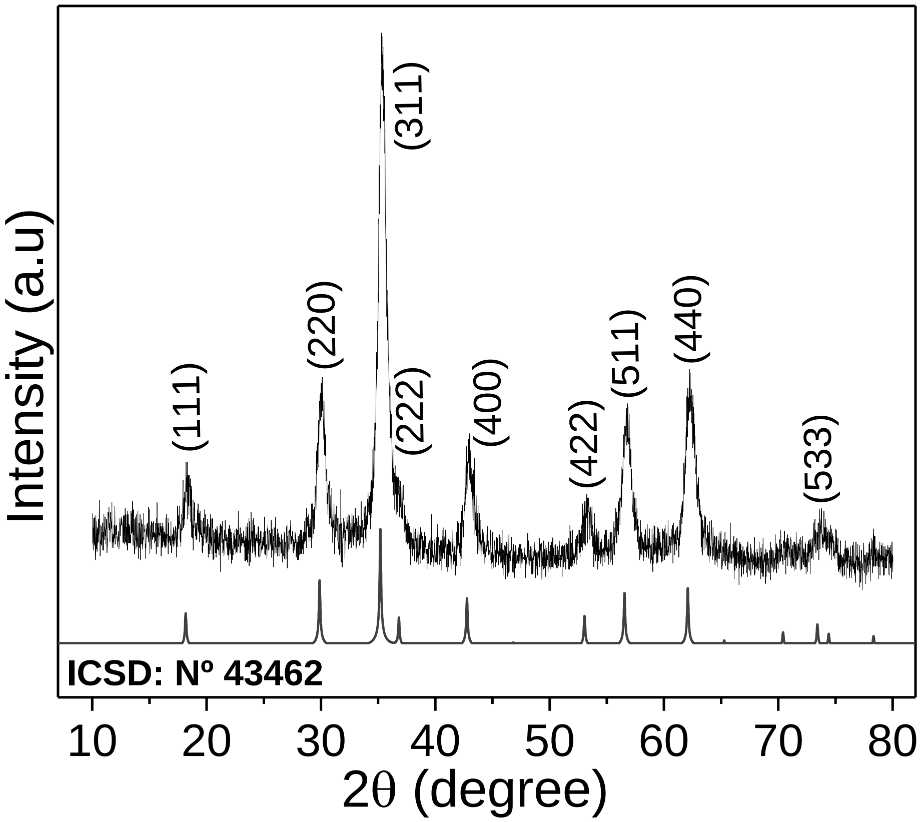

Fig. S18. Crystal structure analysis of the $MnFe_2O_4$-cys NPs. Experimental data of the XRD diffractogram and crystallographic pattern from Inorganic Crystal Structure Database (ICSD) of the cell structure of cubic spinel crystal (*fcc*) of the $MnFe_2O_4$.

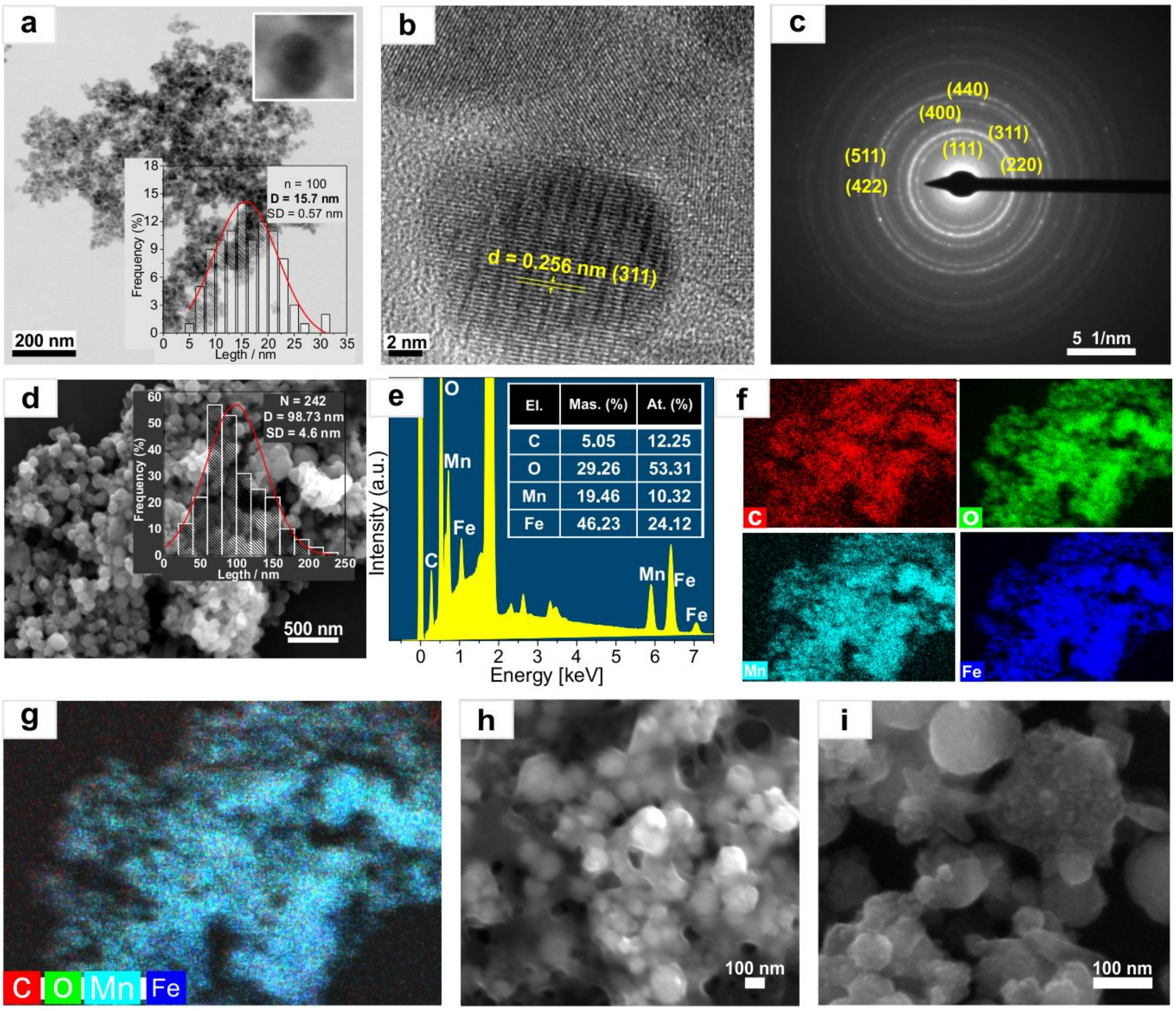

Fig. S19. Morphological characterization of the $MnFe_2O_4$-cys NPs. (a) TEM images and respective size distribution histogram. (b) HRTEM image. (c) SAED pattern. (d) SEM micrograph. (e–g) respective EDX spectra and elemental chemical mapping. (h,i) SEM micrographs in different microregions of the $MnFe_2O_4$-cys NPs.

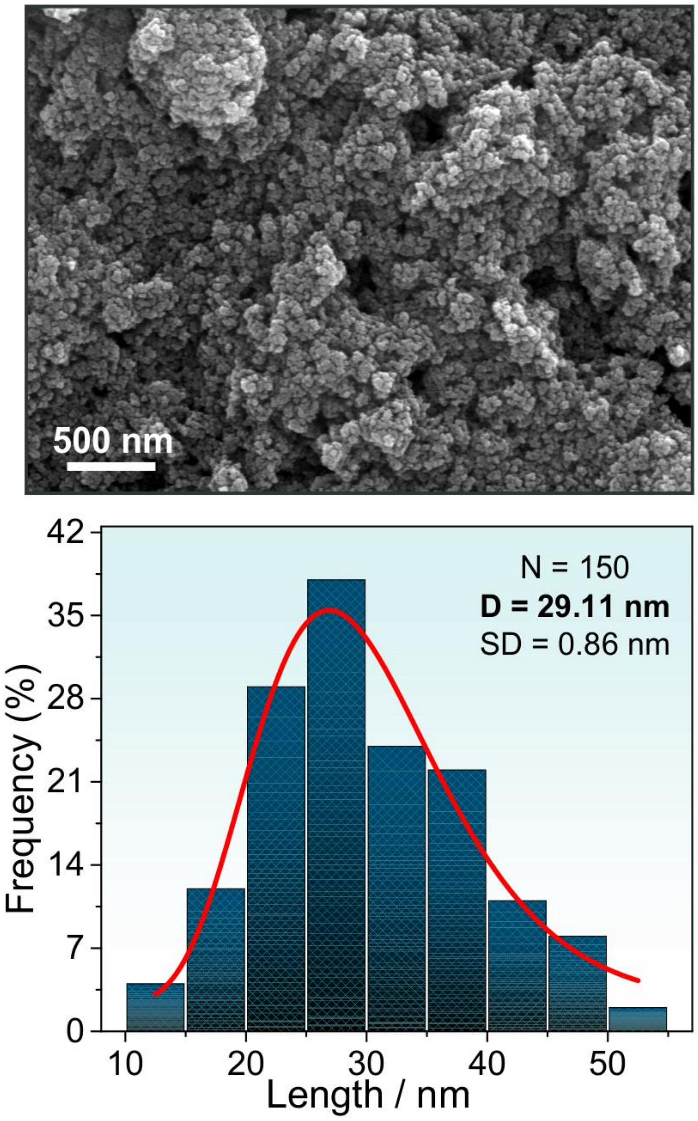

Fig. S20. Structural organization and distribution of nanoparticles of the bioconjugate. SEM micrograph (x25,000 magnification and 15kV acceleration voltage) of $MnFe_2O_4$–EDC:NHS-S1-Ab and corresponding size distribution histogram. The average size of the nanoparticles was defined from the Gaussian curve.

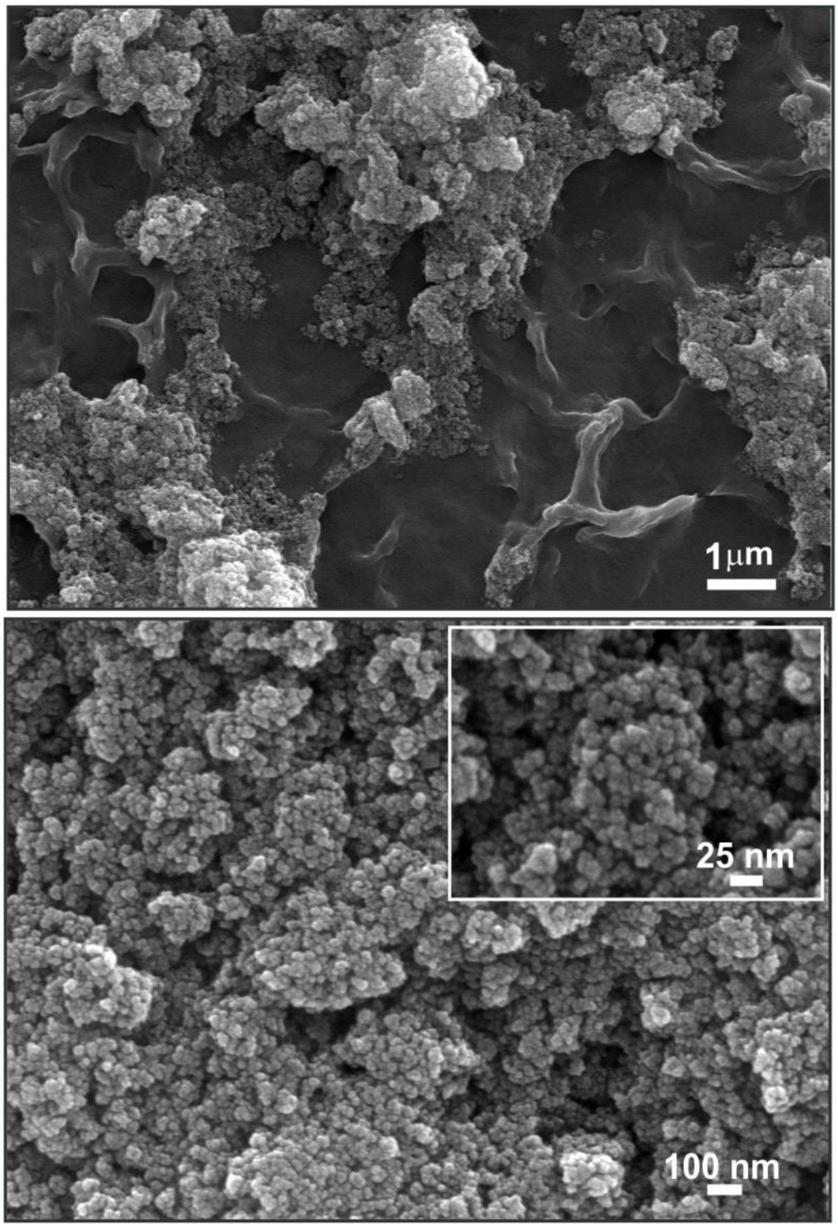

Fig. S21. Morphology of $MnFe_2O_4$ NPs modified with EDC and NHS. SEM micrographs of $MnFe_2O_4$–EDC:NHS at 15kV acceleration voltage and different magnifications (x10,000, x50,000 and x150,000).

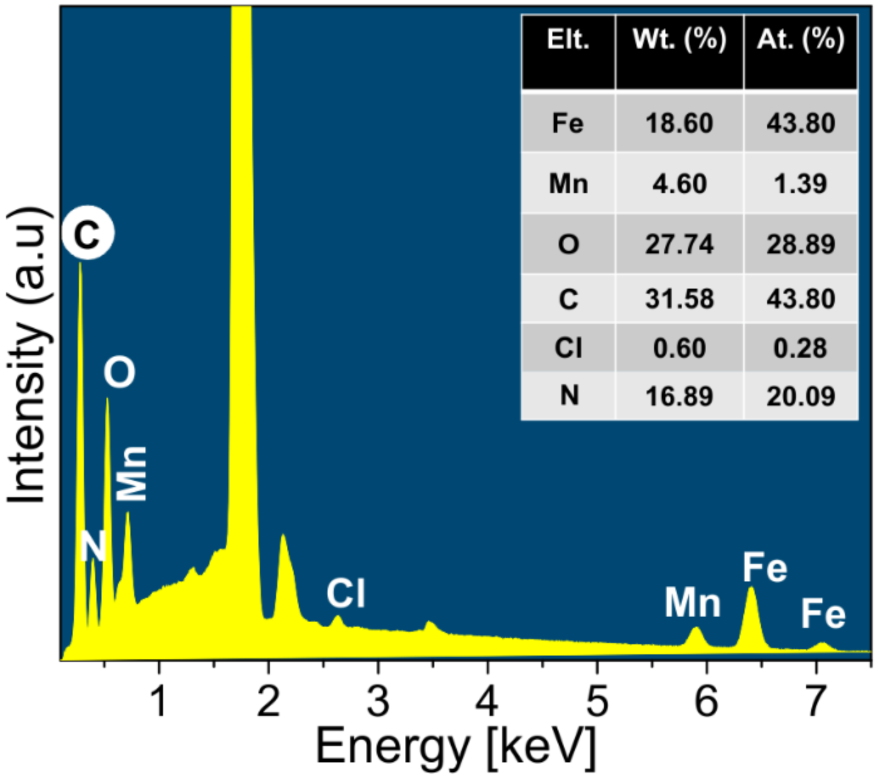

Fig. S22. Microanalysis of the chemical composition of the $MnFe_2O_4$ NPs modified with EDC and NHS. Spectra SEM-EDX and composition values for $MnFe_2O_4$–EDC:NHS.

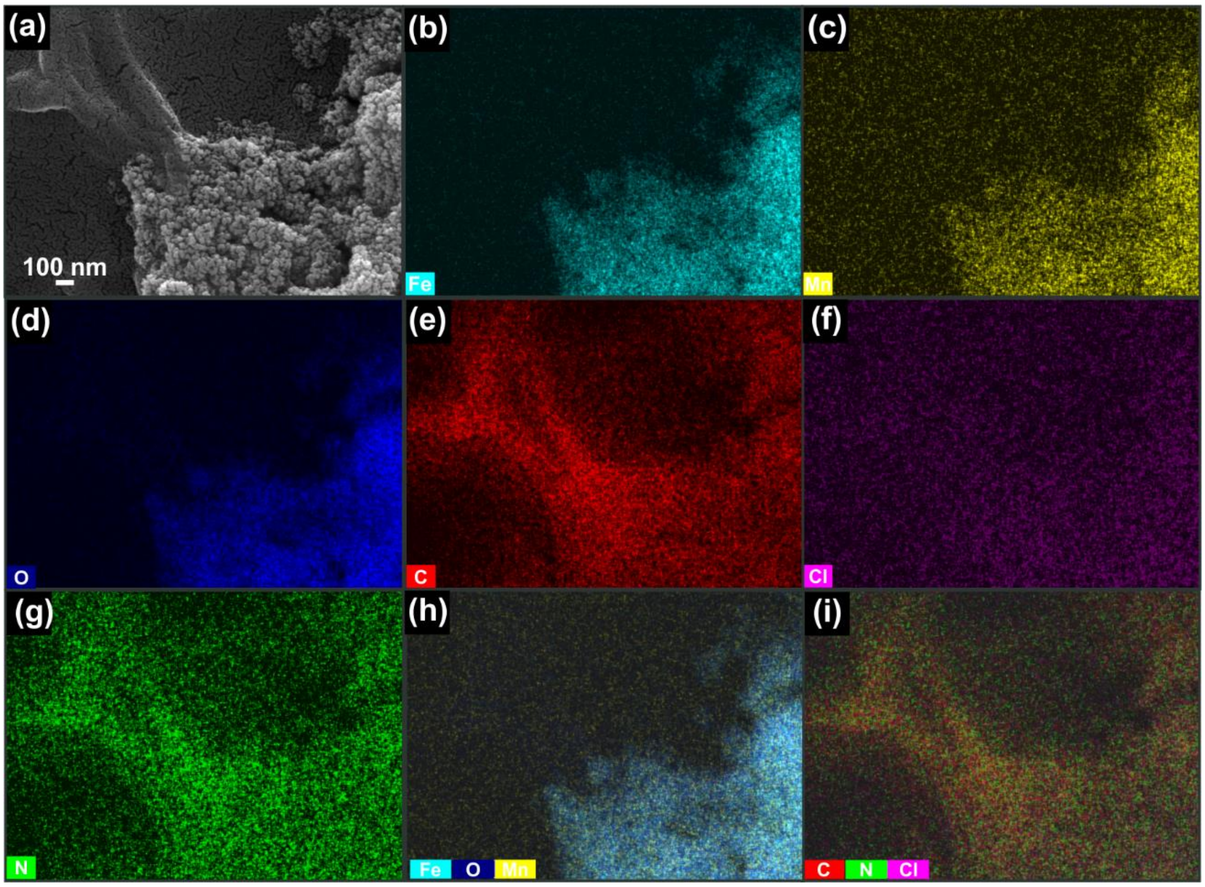

Fig. S23. Chemical micro-mapping of the $MnFe_2O_4$ NPs modified with EDC and NHS.(a) SEM of the mapped area (b-i) EDX elemental mapping of Fe, Mn, O, C, Cl, N, Fe-O-Mn, and C-N-Cl for $MnFe_2O_4$–EDC:NHS. Conditions: 15kV acceleration voltage and x50,000 magnification.

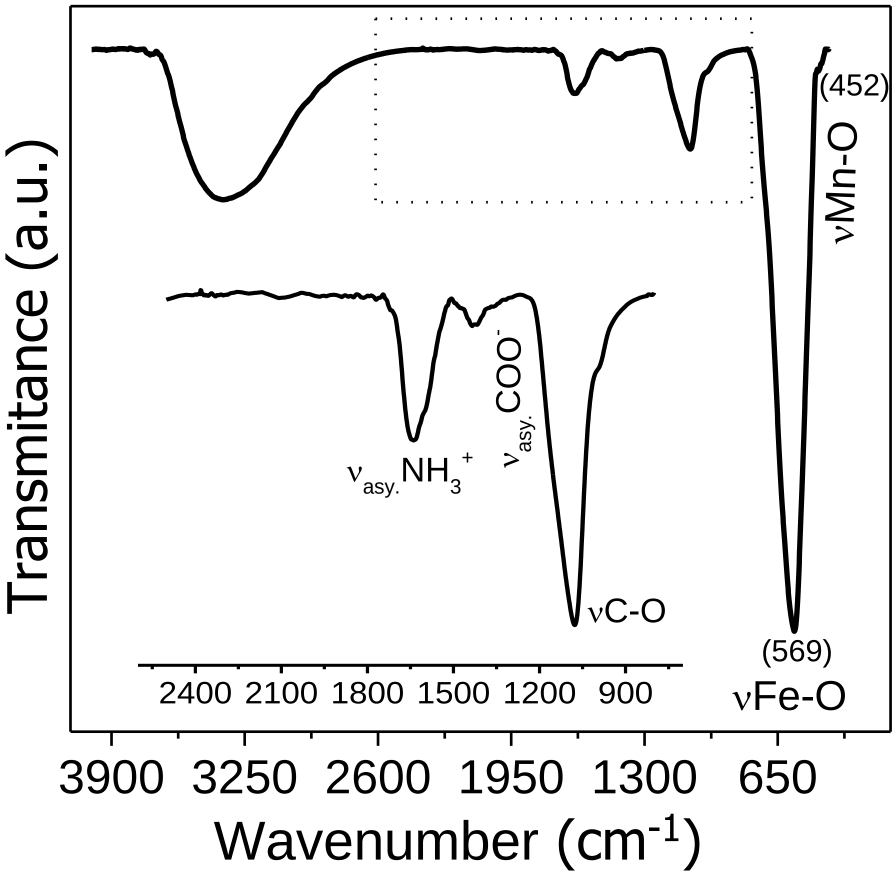

Fig. S24. Fourier transform infrared spectroscopy (FTIR) for functionalized with cysteine. FTIR spectrum of $MnFe_2O_4$-cys in KBr pellet between 4000 to 400 cm^{-1} . The highlighted region shows the vibrational modes of the main chemical groups of cysteine.

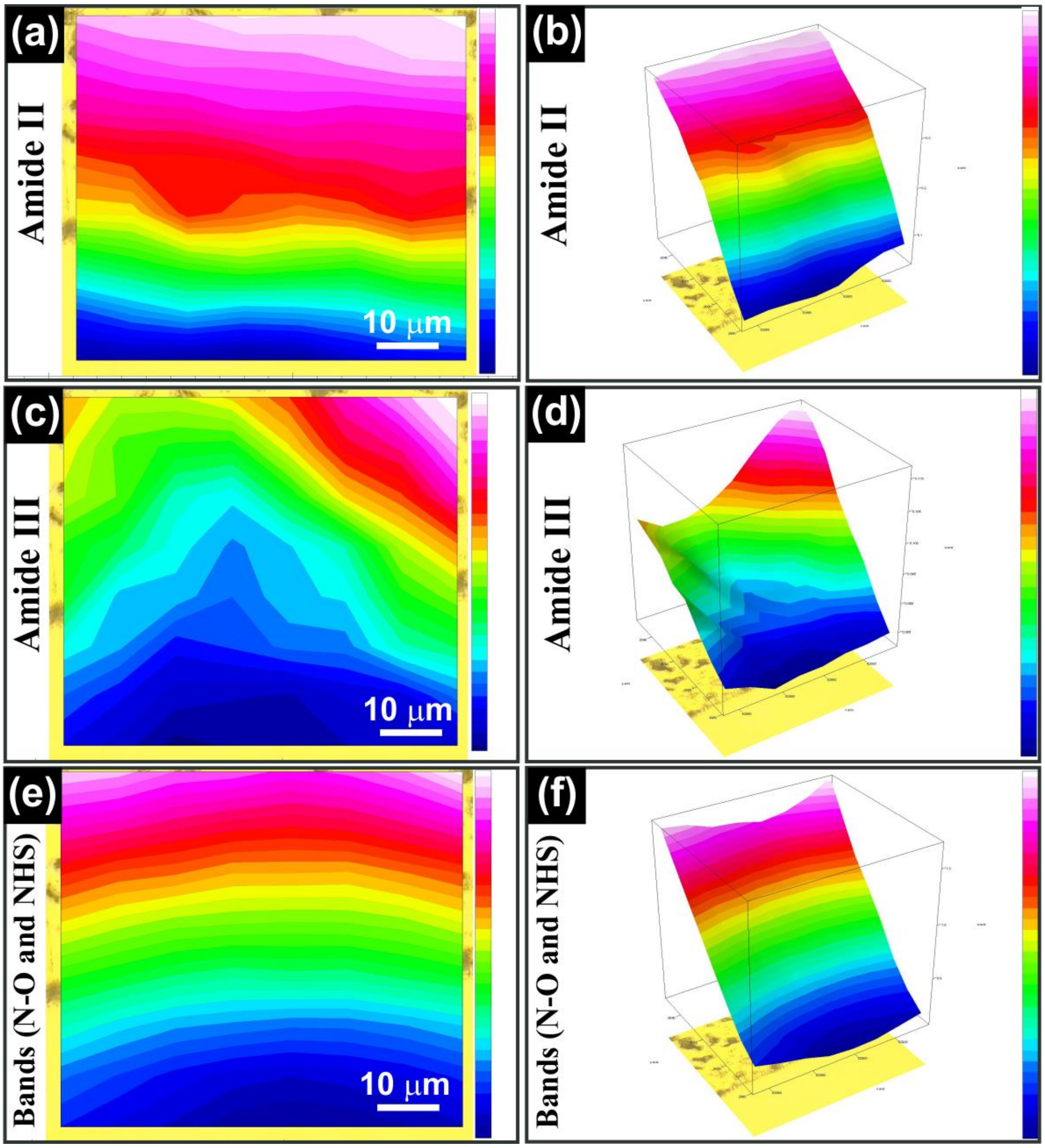

Fig. S25. Chemical images of the bioconjugate. Micro-FTIR spectroscopy of the $MnFe_2O_4$–EDC:NHS-S1-Ab – 2D chemical and 3D image obtained of the region of amide II (a, b), 2D chemical and 3D image obtained of the region of amide III (c,d), 2D chemical and 3D image obtained of the N-O and NHS bands, referent to EDC:NHS chemical coupling (e,f). The bands concentration at each pixel is interpreted according to the color gradient of the chemical maps, red cor indicated higher bands signals.

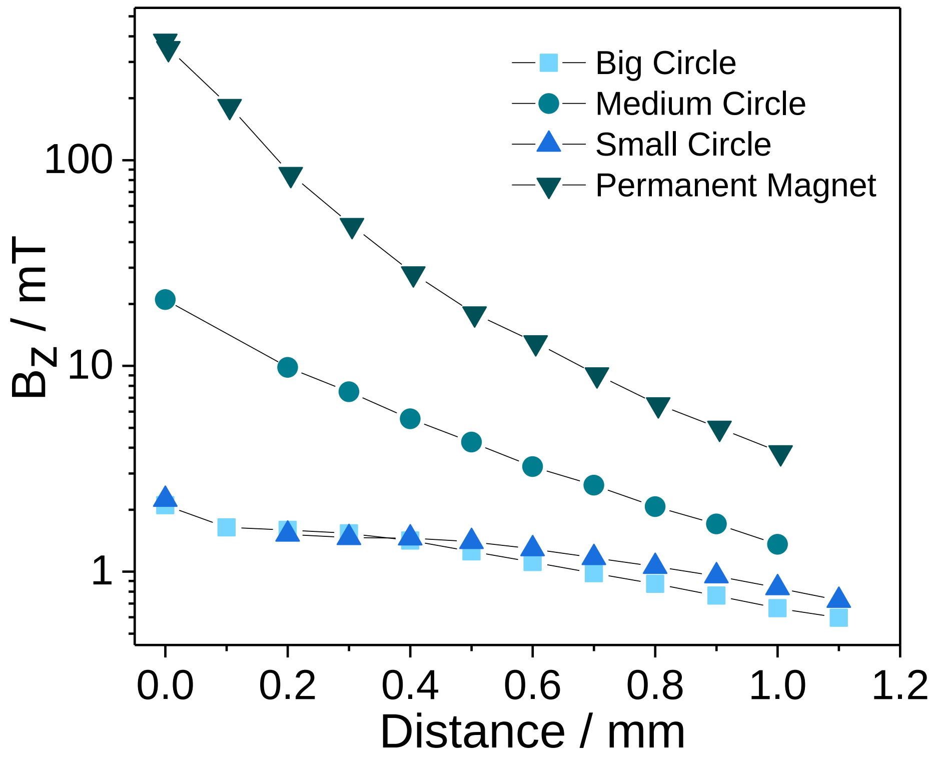

Fig. S26. Investigation of magnetic properties on the electrode surface with an integrated magnet. Behavior of the magnetic field as a function of the distance of the MED surface.

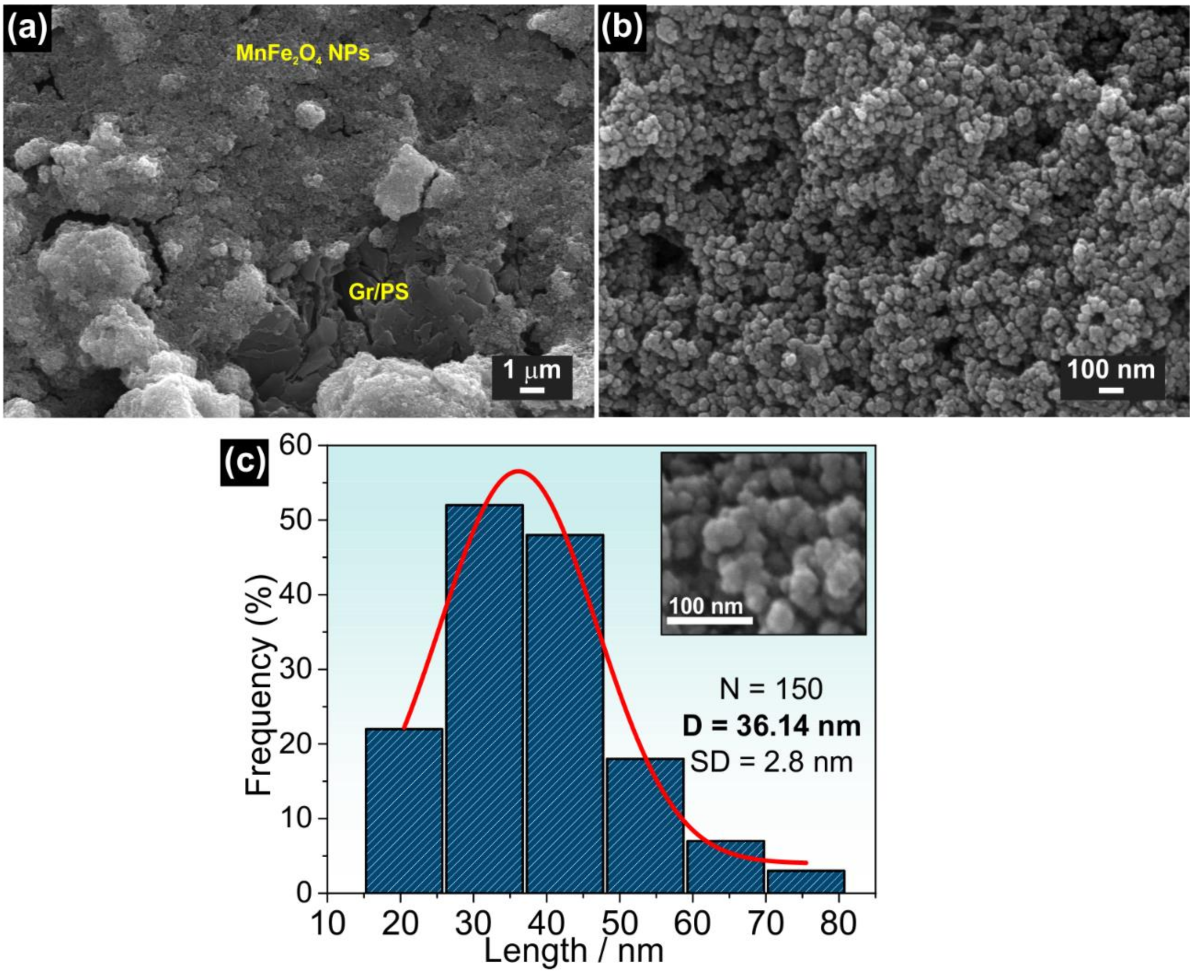

Fig. S27. Structural organization and distribution of $MnFe_2O_4$ NPs onto MED surface. (a-b) SEM images different microregions (x5,000 and x50,000 magnification), and (c) SEM micrograph inserted (x150,000 magnification) and corresponding size distribution histogram $MnFe_2O_4$|MED. The average size of the nanoparticles was defined from the curve based in the log-normal distribution function.

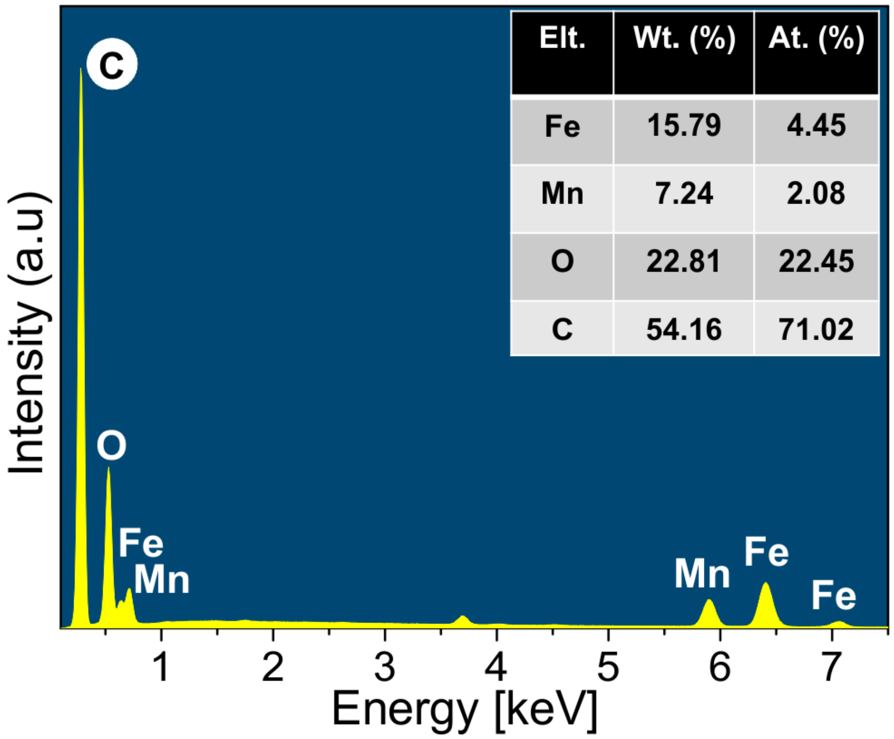

Fig. S28. Microanalysis of the chemical composition for electrode modified with $MnFe_2O_4$ NPs. Spectra SEM-EDX and percentage composition values for $MnFe_2O_4$|MED.

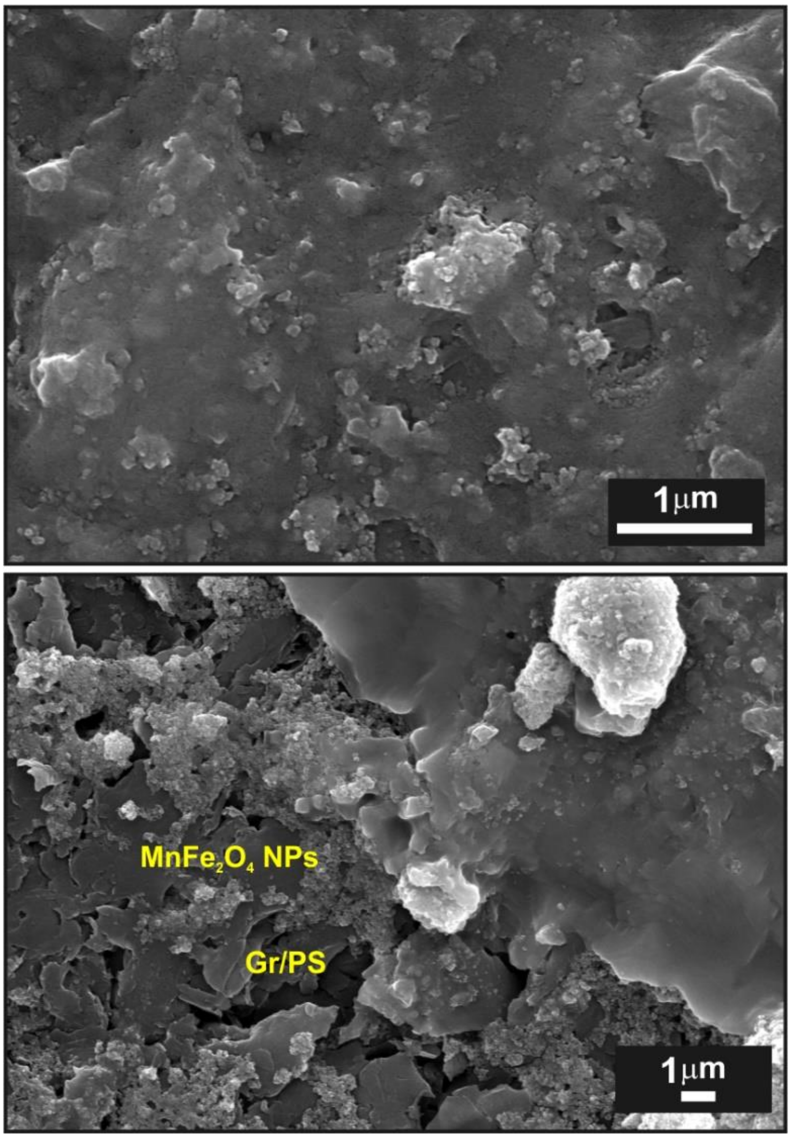

Fig. S29. Morphology surface of the MED modified with bioconjugate. SEM images different microregions for $MnFe_2O_4$-EDC:NHS/S1-Ab|MED. Conditions: 15kV acceleration voltage and x20,000 and x5,000 magnification.

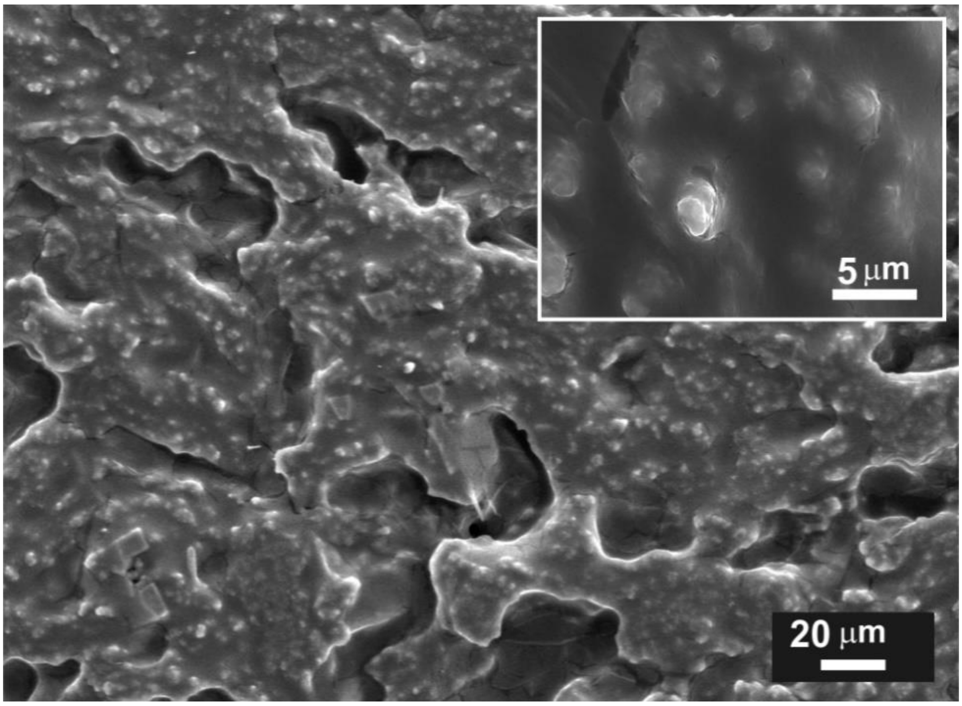

Fig. S30. Morphology surface of the MED modified with $MnFe_2O_4$-EDC:NHS|MED. SEM images different microregions for $MnFe_2O_4$-EDC:NHS|MED. Conditions: 15kV acceleration voltage and x400 and x5,000 magnification. The EDC and NHS covering surface does not allow for more detailed visualization of the $MnFe_2O_4$ NPs.

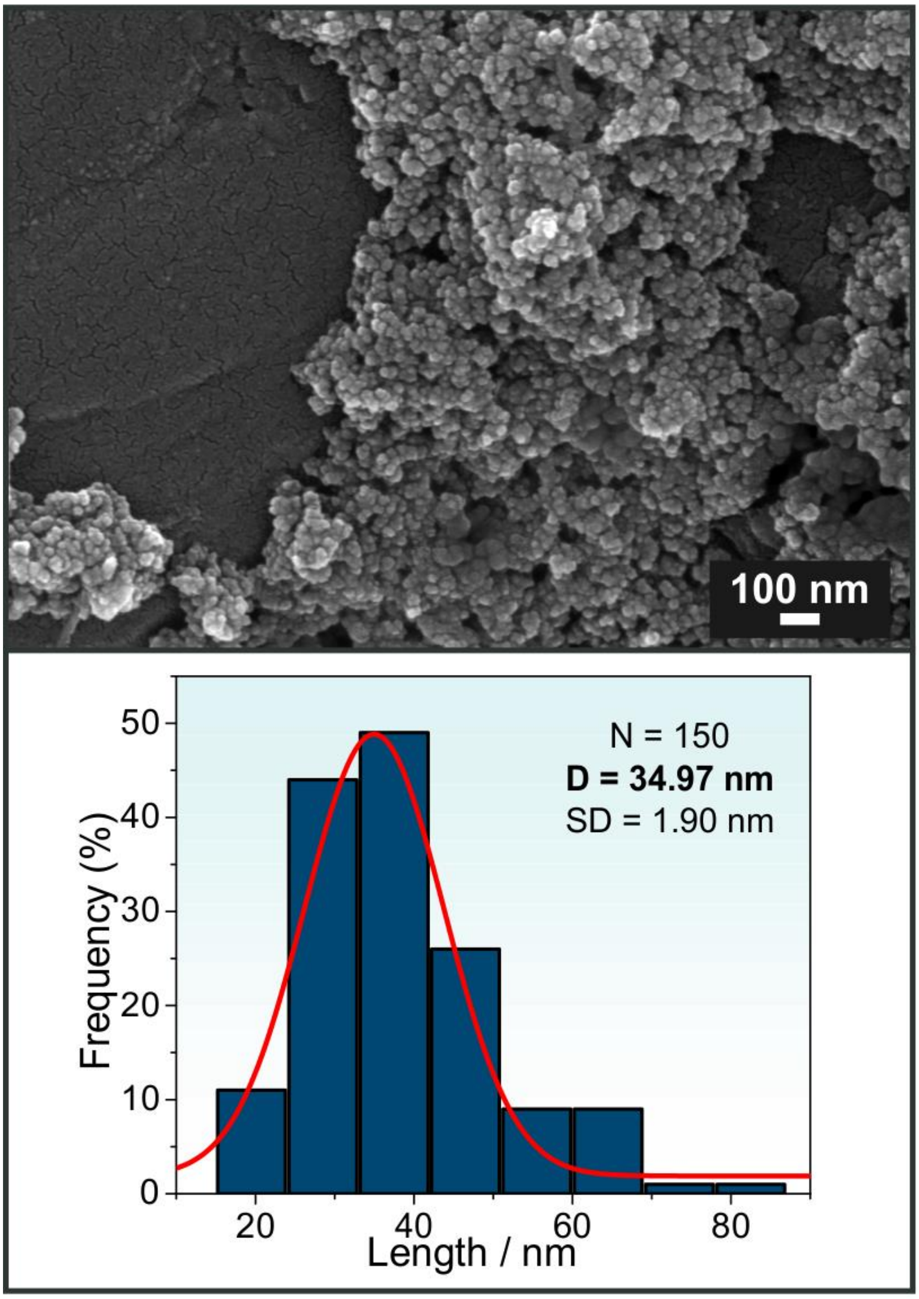

Fig. S31. Structural organization and distribution of bioconjugate onto MED surface. SEM micrograph (x50,000 magnification and 15kV acceleration voltage) of $MnFe_2O_4$-EDC:NHS/S1-Ab|MED and corresponding size distribution histogram. The average size of the nanoparticles was defined from the curve based in the log-normal distribution function.

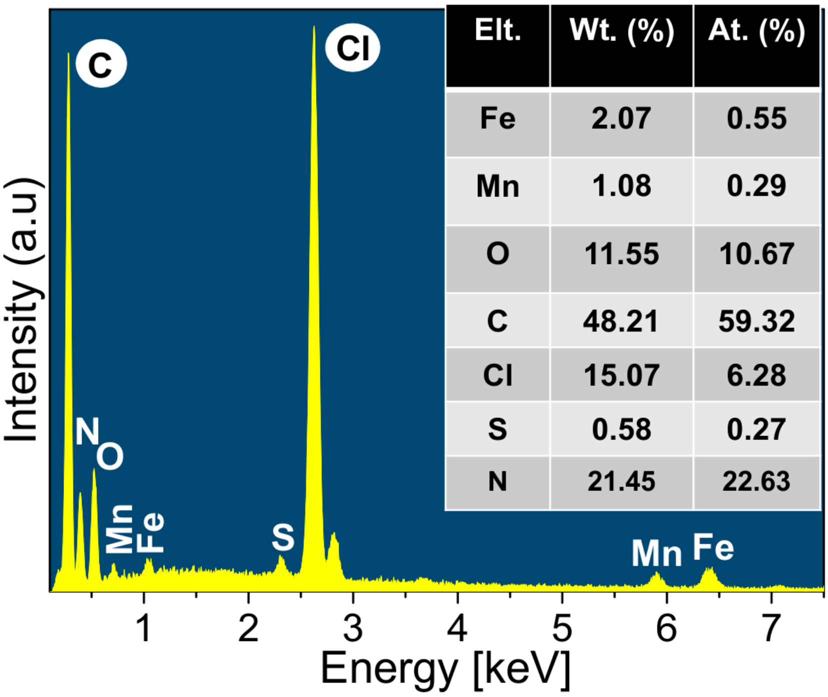

Elt.	Wt. (%)	At. (%)
Fe	2.07	0.55
Mn	1.08	0.29
O	11.55	10.67
C	48.21	59.32
Cl	15.07	6.28
S	0.58	0.27
N	21.45	22.63

Fig. S32. Microanalysis of the chemical composition for MED modified with $MnFe_2O_4$-EDC:NHS. Spectra SEM-EDX and percentage composition values for $MnFe_2O_4$-EDC:NHS|MED .

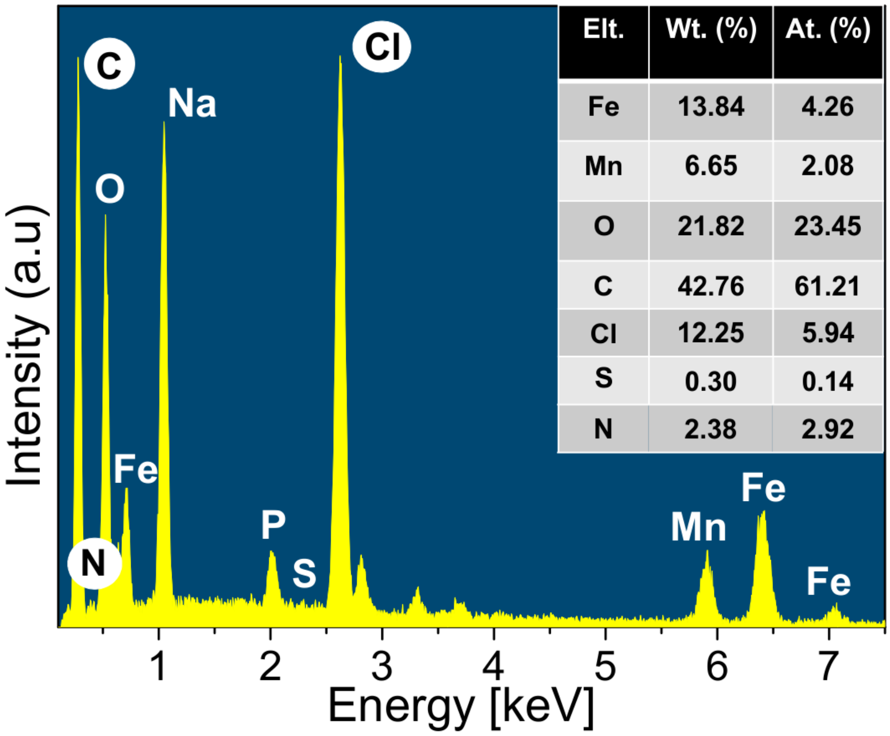

Elt.	Wt. (%)	At. (%)
Fe	13.84	4.26
Mn	6.65	2.08
O	21.82	23.45
C	42.76	61.21
Cl	12.25	5.94
S	0.30	0.14
N	2.38	2.92

Fig. S33. Chemical composition for MED modified with bioconjugate. Spectra SEM-EDX and percentage composition values for $MnFe_2O_4$-EDC:NHS/S1-Ab|MED.

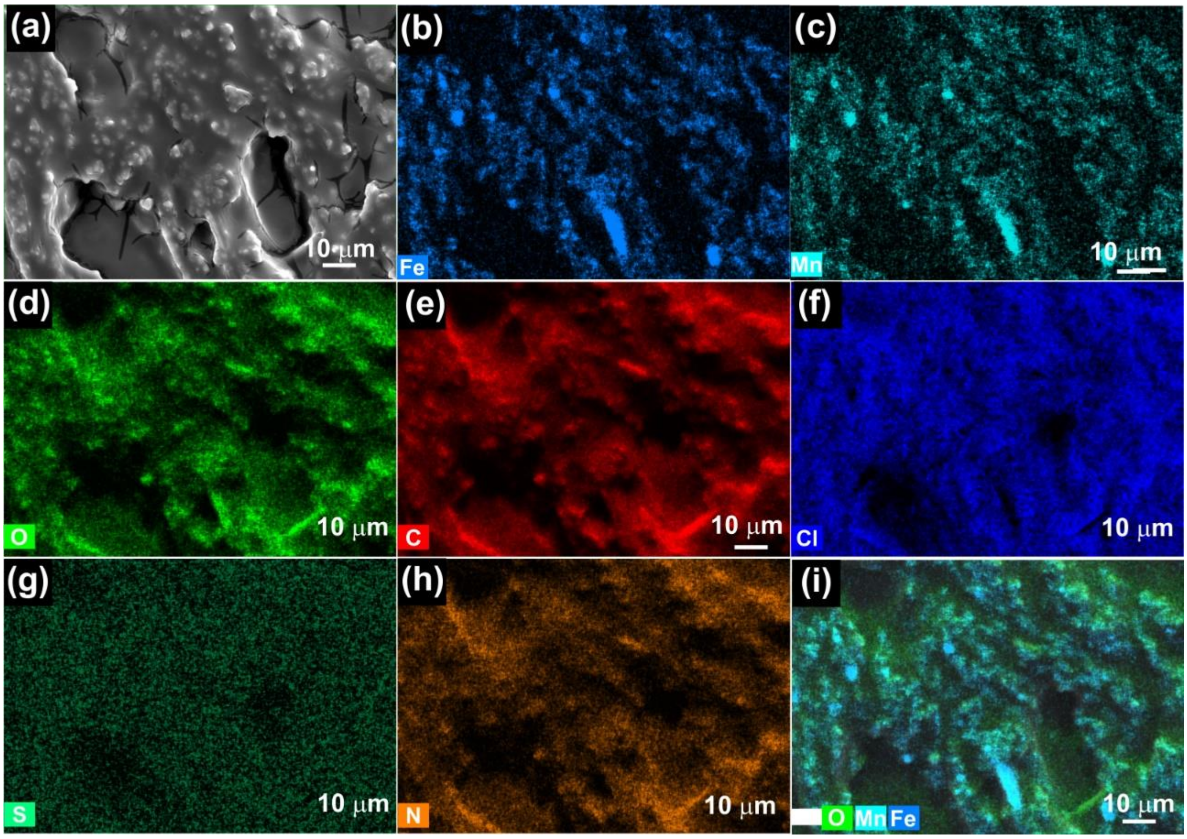

Fig. S34. Chemical mapping of the MED modified with $MnFe_2O_4$-EDC:NHS nanoparticles. (a) SEM of the mapped area (b-i) EDX elemental mapping of Fe, Mn, O, C, Cl, S, N and O-Mn-Fe, and C-N-Cl for $MnFe_2O_4$-EDC:NHS|MED. Conditions: 15kV acceleration voltage and x1,000 magnification.

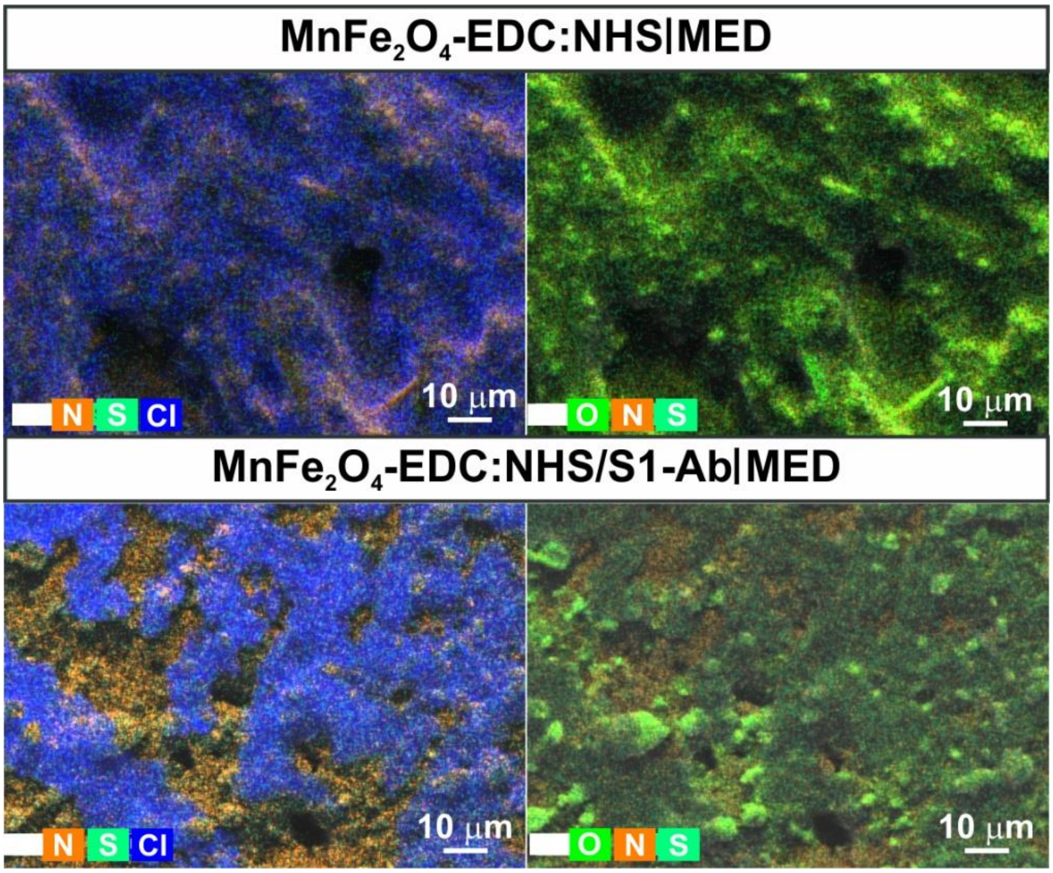

Fig. S35. Chemical micro-mapping of the MED modified with $MnFe_2O_4$-EDC:NHS nanoparticles and bioconjugate.(a) Mapping of N-S-Cl and O-N-S for $MnFe_2O_4$-EDC:NHS|MED and $MnFe_2O_4$-EDC:NHS/S1-Ab|MED. For interpretation purposes we consider the combination of N-S-Cl elements characteristic of the distribution of EDC and NHS. While the combination of O-N-S refers to the distribution of elements that are part of the chemical composition of the S1-Ab antibody. Conditions: 15kV acceleration voltage and x1,000 magnification.

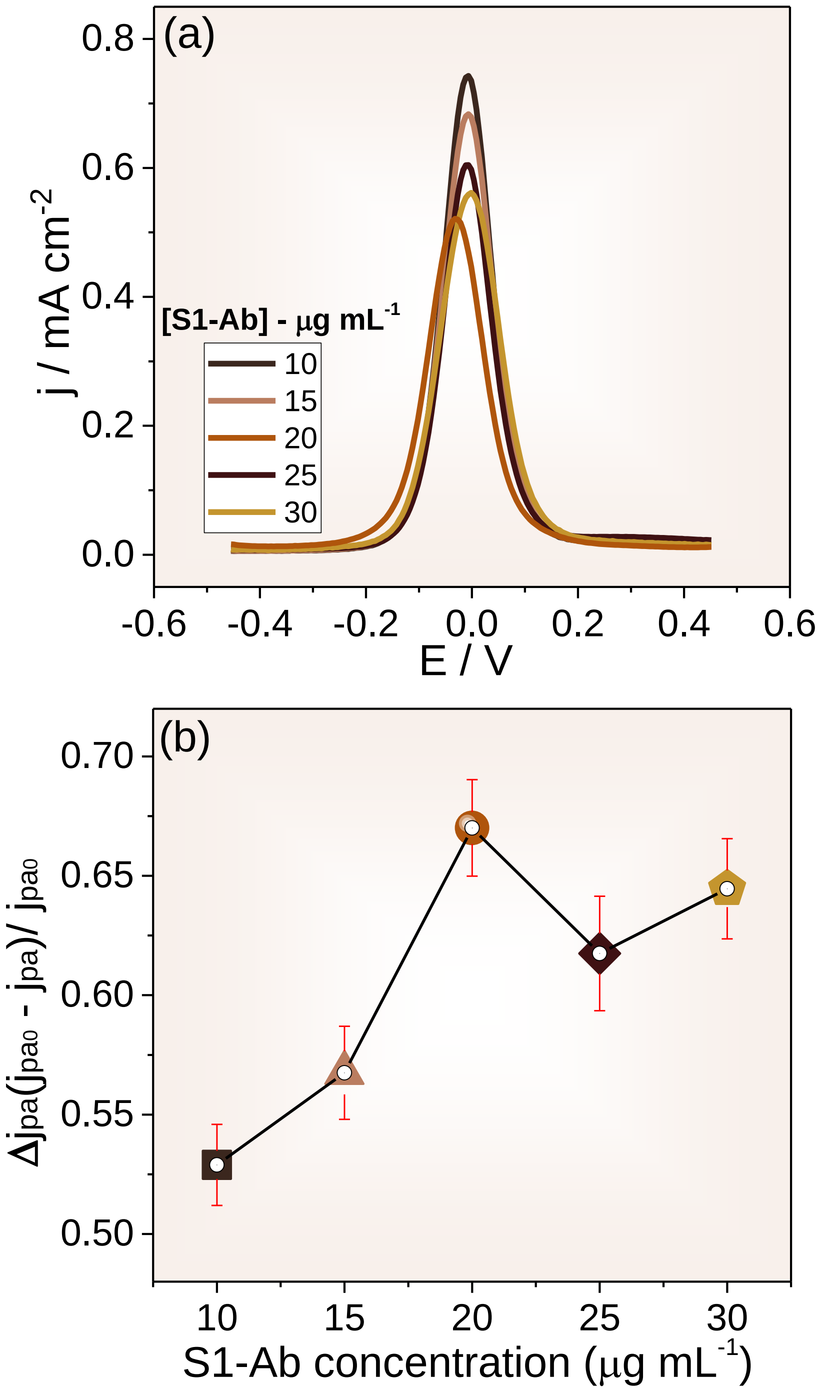

Fig. S36. Study of the optimization experimental conditions. (a) DPV for S1-Ab concentration from 10 μg mL^{-1} to 30 μg mL^{-1}, (b) (jpa_0 - jpa/ jpa_0) *versus* S1-Ab concentration. These experiments are crucial for reducing the costs of detection tests. 95% confidence intervals for all error bars (n = 3).

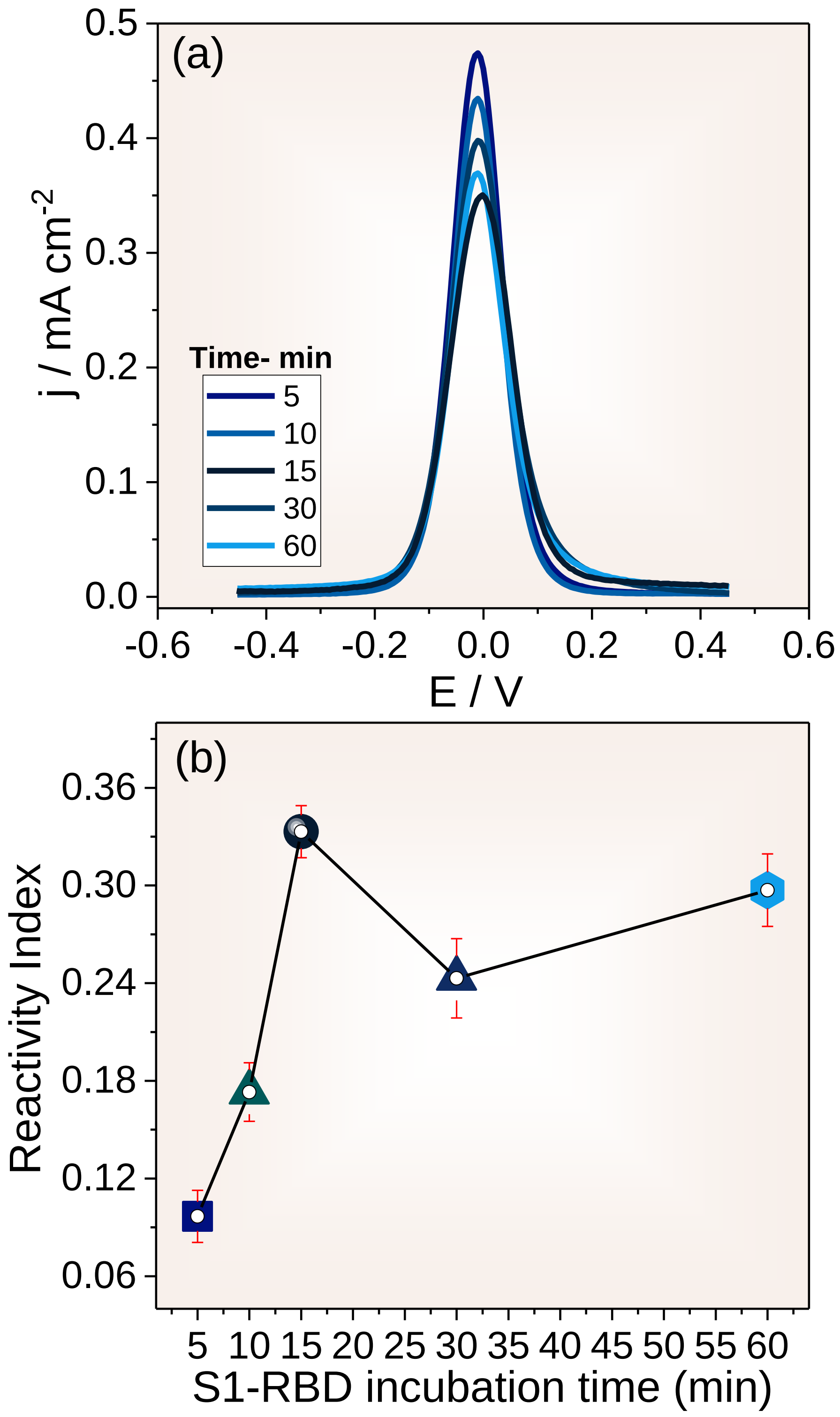

Fig. S37. Optimization of immunoreaction time.(a) DPV for S1-RBD incubation time from 5 to 60 min. (b) reactivity index *versus* S1-RBD (0.5 ng mL^{-1}) incubation time. These experiments are important to find the best time for detection tests. 95% confidence intervals for all error bars (n =3).

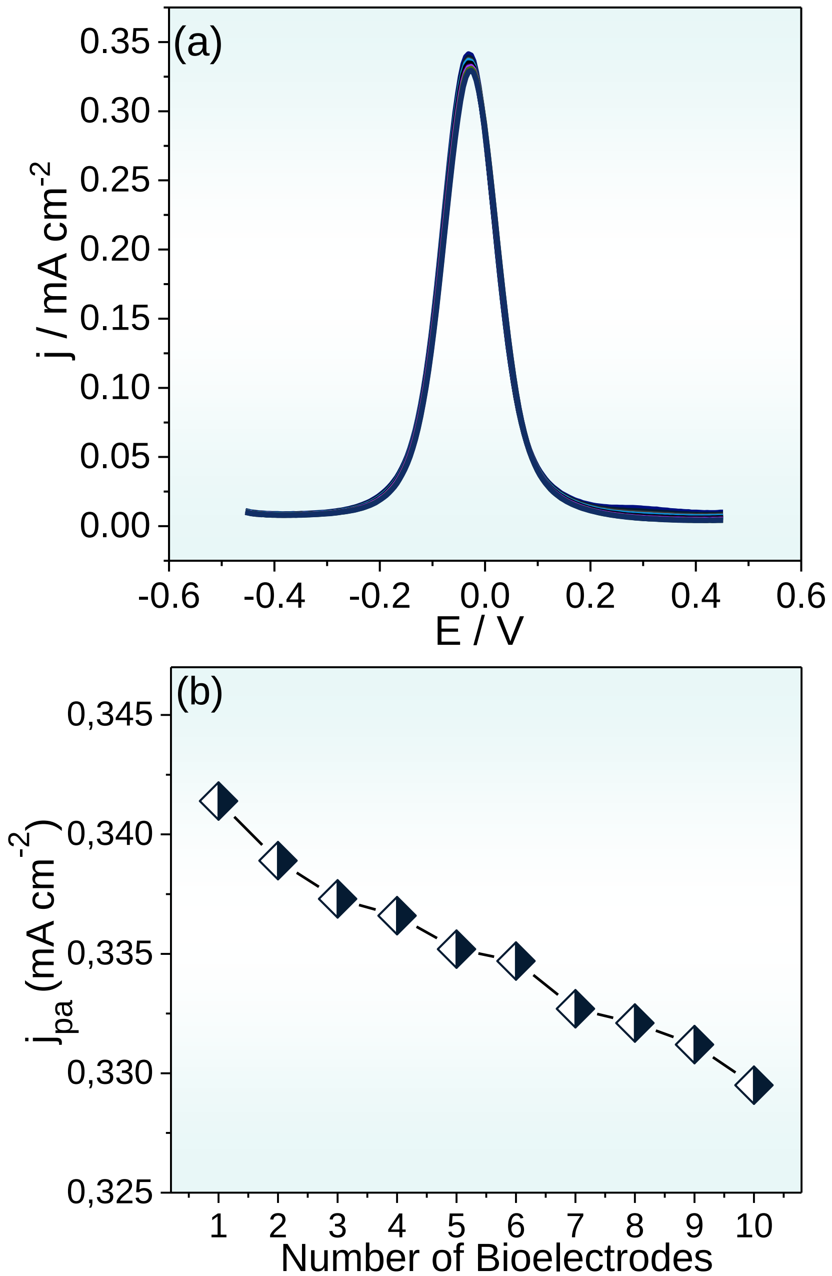

Fig. S38. Electrochemical behavior of the same biosensor for magnetic detection immunoassays. (a) DPV reponses for ten bioelectrodes + RBD (intraelectrode), and (b) corresponding j_{pa} *versus* $MnFe_2O_4$-EDC:NHS/S1-Ab/BSA|MED.

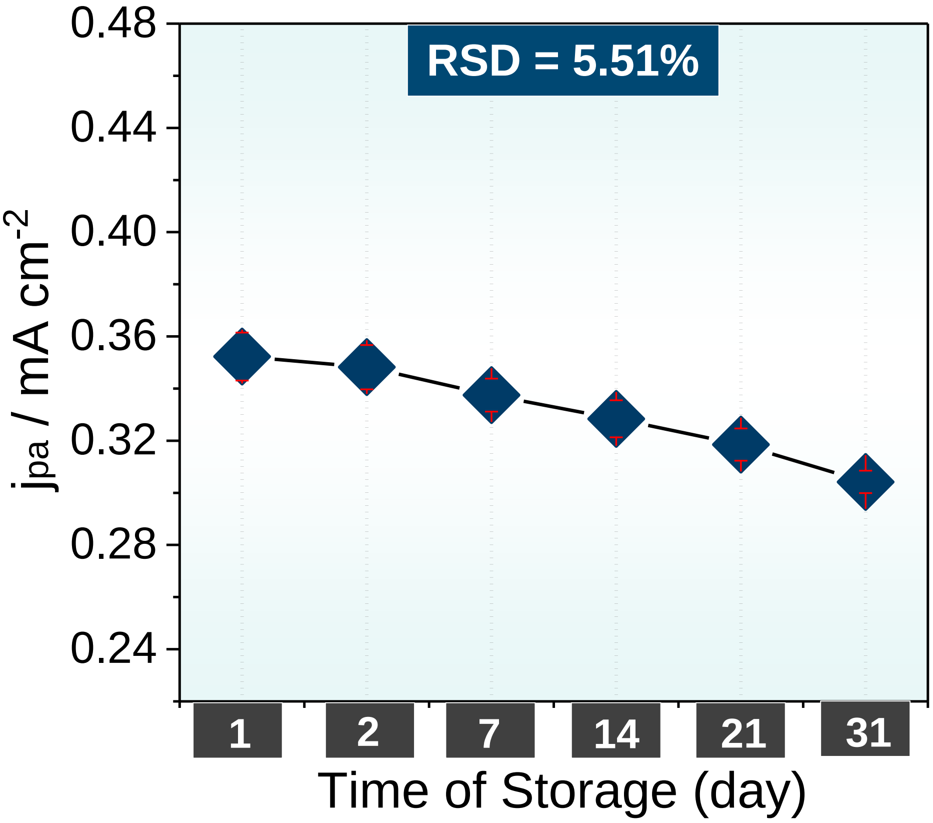

Fig. S39. Profile of current densities of storage stability study for different biosensors. Plot of j_{pa} *versus* time of storage for magnetic immunoassays onto $MnFe_2O_4$-EDC:NHS/S1-Ab/BSA|MED. 95% confidence intervals for all error bars (n = 3).

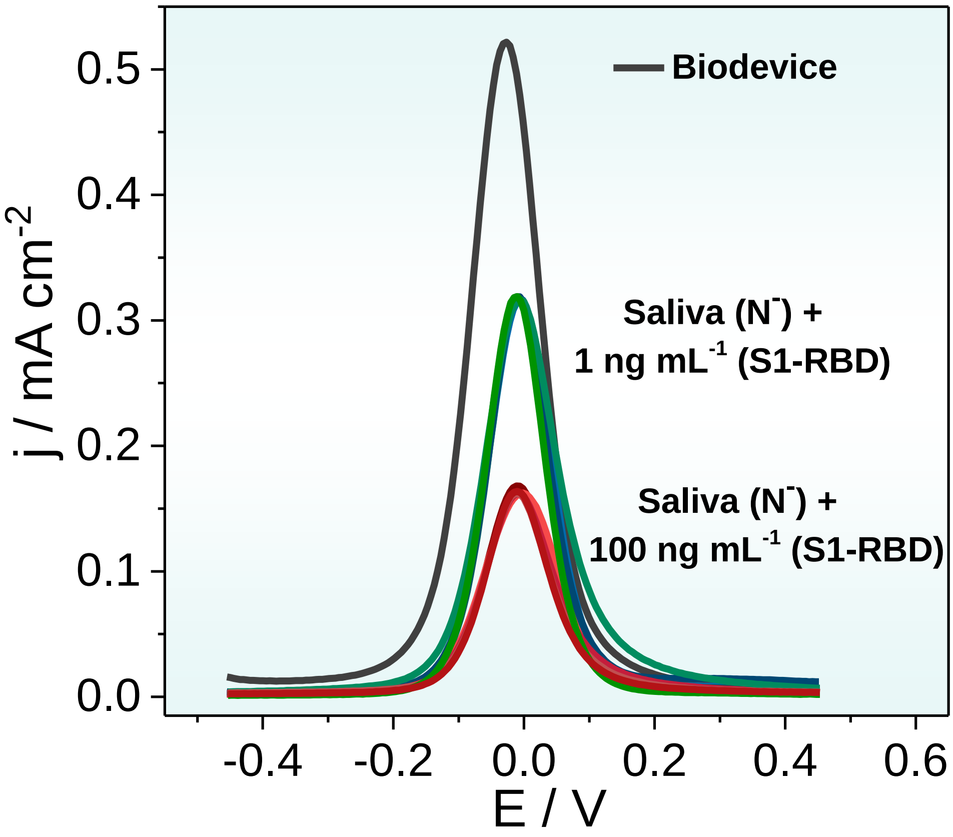

Fig. S40. Electrochemical behavior of the biosensor for recovery assays in saliva samples. DPV Voltammograms obtained from independent measurements with MgNPs-S1-Ab/BSA|MED biosensor in negative saliva samples. To have a representative S1-RBD concentration range, we use a concentration that differs on the order of x100.

Table S1. Experimental data to estimate the recyclability of the spent Zn/C batteries.

Components	Average weights
Upper steel cover	0.177 g
Bottom steel cover	0.22 g
Carbon rod	1.06 g
Plastic film	0.24 g
Cathode mix	7.67 g
Anode zinc	2.98 g
External wrap	2.52 g
Others (non-recyclable)	0.793 g
Mass total Zn/C batteries:	15.64 g
**Recyclability Rate = (14.85 / 15.64) x 100 = 94.95 %**	

Table S2. Comparison of the MED with some articles that used circular economy concept for preparation of electrochemical devices.

Sustainable approach	Composition of the devices		Ref.
	Conductive	Polymeric binder	
Water-based ink	Silver flakes	Polyurethane	[1]
Water-based ink	Graphene and functionalized multi-walled	Mixture of styrene acrylic resins	[2]
Recyclable substrate	Graphite	Nail polish	[3]
Biodegradable materials	Graphen nanoplatelets	Polylactic acid	[4]
Recyclable-based ink	Graphite from spent Zn-C batteries	Polystyrene of disposable cups	This work

Table S3. Values of the main studied magnetic parameters of the investigated nanomaterials.

Nanomaterial	Properties
$MnFe_2O_4$ NPs	Ms = 48.836± 0.152 emu g^{-1}<br>Mr = 0.703± 0.02 emu g^{-1}<br>Hc = 0.489± 0.022 mT
$MnFe_2O_4$-EDC:NHS/S1-Ab/BSA	Ms = 39.069± 0.033 emu g^{-1}<br>Mr = 0.494± 0.04 emu g^{-1}<br>Hc = 0.474± 0.014 mT

Ms: saturation magnetization; Mr: remaining magnetization; Hc: coercive force

Table S4. Electroanalytical performance of the developed electrochemical-magnetic biosensor.

Biosensor (Transducer type)	Method	Linear range	LOD	Sample	Interaction time	Ref.
Ag-rGO|Au electrode	DPV	0.015-158.5 pg mL^{-1}	7.2 fg mL^{-1}	Artificial saliva	40 min.	[5]
Aptamer/AuNP-CNF|CSPE	EIS	0.35 ng mL^{-1}-2.24 µg mL^{-1}	0.24 ng mL^{-1}	Saliva	40 min.	[6]
Aptamer-MB|Au electrode	SWV	0.35-3.5 ng mL^{-1}	0.35 ng mL^{-1}	Artificial saliva	5 min.	[7]
Aptamer gated-MB@MPSF|LEGE	SWV	0.5-250 ng mL^{-1}	0.36 ng mL^{-1}	Saliva	30 min.	[8]
Aptamer/Au_{nano}-S-Si-NPAAO|LEGE	SWV	2.5-40 ng mL^{-1}	0.8 ng mL^{-1}	Saliva	20 min.	[9]
SPCE|ceria/STV/Apt	DPV	5-35 ng mL^{-1}	1.94 ng mL^{-1}	not detected	60 min.	[10]
Aptamer biotinylated/Au_{nano}-MPA|SPE	DPV	10-50 ng mL^{-1}	2.63 ng mL^{-1}	Saliva	40 min.	[11]
MgNPs/S1-Ab/BSA|MED	DPV	5 pg mL^{-1} - 5 µg mL^{-1}	3.46 pg mL^{-1}	Saliva	15 min.	This work

Ag-rGO/Au electrode: gold electrode modified with nanocomposite of silver nanoparticles and reduced graphene oxide

Aptamer/AuNP-CNF/CSPE: carbon-based screen-printed electrode modified with carbon nanofiber–gold nanoparticle and thiol-terminal aptamer

Aptamer-MB/Au electrode: gold electrode modified with derivative of methylene blue and aptamer

Aptamer gated-MB@MPSF/LEGE: laser engraved graphene electrode modified with aptamer-gated methylene blue@mesoporous silica film

Aptamer/Au_{nano}-S-Si-NPAAO/LEGE: laser engraved graphene electrode modified with nanoporous anodic aluminium oxide membrane functionalized by 3-mercaptopropyl trimethoxysilane, gold nanoparticles and thiol-terminal aptamer

SPCE/ceria/STV/Apt: screen-printed carbon electrode modified with ceria nanoparticles, streptavidin and thiol-terminal aptamer

Aptamer biotinylated/Au_{nano}-MPA/SPE: screen-printed carbon modified with gold nanoparticles functionalized by 3-mercaptopropionic acid and biotinylated aptamer

Table S5. Details of data obtained in recovery tests.

**Negative saliva samples (ID patients healthy)**	**$C_{S1\text{-}RBD\,added}$ (ng m L^{-1})**	**$C_{S1\text{-}RBD\,founded}$ (mean±SD; ng m L^{-1})**	**Recovery (%)**	**RSD (%) (n = 3)**
10029246904	1.0	1.02±0.03	102	2.94
	100.00	98.45±3.25	98.45	3.31
1002929252804	1.0	1.03±0.04	103	3.88
	100.00	99.62±2.78	99.62	2.79
100292402504	1.0	0.9835±0.0427	98.35	4.34
	100.00	101.6±3.17	101.6	3.12
120001008304	1.0	1.05±0.05	105	4.76
	100.00	100.9±2.49	100.9	2.47
120001015504	1.0	0.9788±0.0356	97.88	3.64
	100.00	95.74±4.45	95.74	4.65

Table S6. Main parameters obtained in the ROC curve construction procedure.

Criterion	Sensitivity	95% CI	Specificity	95% CI	+LR*	-LR**
≥ 0.0142	100.00	47.8-100	0.00	0.0-21.8	1.00	
> 0.0142	100.00	47.8-100	6.67	0.2-31.9	1.07	0.00
> 0.0216	100.00	47.8-100	13.33	1.7-40.5	1.15	0.00
> 0.0243	100.00	47.8-100	20.00	4.3-48.1	1.25	0.00
> 0.0254	100.00	47.8-100	26.67	7.8-55.1	1.36	0.00
> 0.0284	100.00	47.8-100	33.33	11.8-61.6	1.50	0.00
> 0.0352	100.00	47.8-100	40.00	16.3-67.7	1.67	0.00
> 0.0382	100.00	47.8-100	46.67	21.3-73.4	1.87	0.00
> 0.03925	100.00	47.8-100	53.33	26.6-78.7	2.14	0.00
> 0.0434	100.00	47.8-100	60.00	32.3-83.7	2.50	0.00
> 0.0458	100.00	47.8-100	66.67	38.4-88.2	3.00	0.00
> 0.057	80.00	28.4-99.5	66.67	38.4-88.2	2.40	0.30
> 0.064	80.00	28.4-99.5	73.33	44.9-92.2	3.00	0.27
> 0.085	80.00	28.4-99.5	80.00	51.9-95.7	4.00	0.25
> 0.0892	80.00	28.4-99.5	86.67	59.5-98.3	6.00	0.23
> 0.092	80.00	28.4-99.5	93.33	68.1-99.8	12.00	0.21
> 0.145	80.00	28.4-99.5	100.00	78.2-100.0		0.20
> 0.2415	60.00	14.7-94.7	100.00	78.2-100.0		0.40
> 0.257	40.00	5.3-85.3	100.00	78.2-100.0		0.60
> 0.269	20.00	0.5-71.6	100.00	78.2-100.0		0.80
> 0.2742	0.00	0.0-52.2	100.00	78.2-100.0		1.00

* likelihood ratio positive = sensitivity / 100 – specificity

** likelihood ratio negative = 100 – sensitivity / specificity

Table S7. Average reactivity index values for the saliva samples analyzed.

ID Saliva sample	RT-PCR	Biodevice (1)	Biodevice (2)	Biodevice (3)	Reactive index
10029246904	(N^-)	(N^-)	(N^-)	(N^-)	0.0447
1002929252804	(N^-)	(N^-)	(N^-)	(N^-)	0.0371
100292402504	(N^-)	(N^-)	(N^-)	(N^-)	0.0299
COV92I	(P^+)	(P^+)	(P^+)	(P^+)	0.3710
120001010304	(N^-)	(N^-)	(N^-)	(N^-)	0.0284
120001009604	(N^-)	(N^-)	(N^-)	(N^-)	0.0867
120001007604	(N^-)	(N^-)	(N^-)	(N^-)	0.0481
COV98I	(P^+)	(P^+)	(P^+)	(P^+)	0.3388
120001008304	(N^-)	(N^-)	(N^-)	(N^-)	0.0134
120001007304	(N^-)	(N^-)	(N^-)	(N^-)	0.0867
120001017304	(N^-)	(N^-)	(N^-)	(N^-)	0.0914
COV118I	(P^+)	(P^+)	(P^+)	(P^+)	0.3183
120001019704	(N^-)	(N^-)	(N^-)	(N^-)	0.0420
120001015504	(N^-)	(N^-)	(N^-)	(N^-)	0.0438
10030735404	(N^-)	**(P^+)**	(N^-)	(N^-)	0.1018
COV135I	(P^+)	**(N^-)**	(P^+)	(P^+)	0.2422
10030734904	(N^-)	(N^-)	(N^-)	(N^-)	0.0597
10031123904	(N^-)	(N^-)	(N^-)	(N^-)	0.0628
10031126204	(N^-)	(N^-)	(N^-)	(N^-)	0.0707
COV144I	(P^+)	(P^+)	**(N^-)**	(P^+)	0.2697

(N^-) – classification negative; (P^+) – classification positive

Table S8. Main clinical validation criteria for the proposed biosensor.

Biodevice	RT-PCR		Sensitivity: TP / TP + FN	Specificity: TN / TN + FP	Accuracy: TP + TN / TP + FP + TN + FN	Precision: TP / TP + FP
	Positive (*n* = 15)	Negative (*n* = 45)				
**Positive**	13	01	13 / 13 + 02 = 86.67%		13 + 44 / 13 + 01 + 44 + 02 = 95%	13 / 13 + 01 = 92.86%
**Negative**	02	44		44 / 44 + 01 = 97.78%		
**Total**	15	45				

TP - True Positive; TN - True Negative; FP - False Positive; FN - False Negative

Table S9. Analysis of the economic viability of the biosensor.

Material	Approximate quantity	Estimated price (USD)
MED	1000 uni.	0.106
$MnCl_2.4H_2O$	0.016 g	0.006
$FeCl_3.6H_2O$	0.008 g	0.002
L-cysteine	0.0032 g	0.007
NaOH	0.014 g	0.0005
MES	0.0318 g	0.09
EDC	0.800 g	4.48
NHS	0.217 g	0.79
S1-Ab CR3022	0.00038 L	194.6
BSA	0.004 g	0.108
KH_2PO_4	0.0462 g	0.032
Na_2HPO_4	0.048 g	0.0235
**Value per biosensor/test: 0.20 USD**		**Total: 200.24**

References

[1]. L.-C. Jia, C.-G. Zhou, W.-J. Sun, L. Xu, D.-X. Yan, Z.-M. Li, Water-based conductive ink for highly efficient electromagnetic interference shielding coating. *Chemical Engineering Journal* **384**, 123368 (2020).

[2]. A. Koutsioukis, V. Belessi, V. Georgakilas, Solid phase functionalization of MWNTs: an eco-friendly approach for carbon-based conductive inks. *Green Chemistry* **23**, 5442–5448 (2021).

[3]. I. A. de Araujo Andreotti, L. O. Orzari, J. R. Camargo, R. C. Faria, L. H. Marcolino-Junior, M. F. Bergamini, A. Gatti, B. C. Janegitz, Disposable and flexible electrochemical sensor made by recyclable material and low cost conductive ink. *Journal of Electroanalytical Chemistry* **840**, 109–116 (2019).

[4]. M. Najafi, M. Zahid, L. Ceseracciu, M. Safarpour, A. Athanassiou, I. S. Bayer, Polylactic acid-graphene emulsion ink based conductive cotton fabrics. *Journal of Materials Research and Technology* **18**, 5197–5211 (2022).

[5]. A. Wang, Y. Li, X. You, S. Zhang, J. Zhou, H. Liu, P. Ding, Y. Chen, Y. Qi, Y. Liu, C. Liang, X. Zhu, Y. Zhang, E. Liu, G. Zhang, Electrochemical immunosensor nanoarchitectonics with the Ag-rGO nanocomposites for the detection of receptor-binding domain of SARS-CoV-2 spike protein. *Journal of Solid State Electrochemistry* **27**, 489–499 (2023).

[6]. M. Amouzadeh Tabrizi, P. Acedo, An Electrochemical Impedance Spectroscopy-Based Aptasensor for the Determination of SARS-CoV-2-RBD Using a Carbon Nanofiber–Gold Nanocomposite Modified Screen-Printed Electrode. *Biosensors (Basel)* **12**, 142 (2022).

[7]. A. Idili, C. Parolo, R. Alvarez-Diduk, A. Merkoçi, Rapid and Efficient Detection of the SARS-CoV-2 Spike Protein Using an Electrochemical Aptamer-Based Sensor. *ACS Sens* **6**, 3093–3101 (2021).

[8]. M. Amouzadeh Tabrizi, P. Acedo, Highly sensitive aptasensor for the detection of SARS-CoV-2-RBD using aptamer-gated methylene blue@mesoporous silica film/laser engraved graphene electrode. *Biosens Bioelectron* **215**, 114556 (2022).

[9]. M. Amouzadeh Tabrizi, P. Acedo, An electrochemical membrane-based aptasensor for detection of severe acute respiratory syndrome coronavirus-2 receptor-binding domain. *Appl Surf Sci* **598**, 153867 (2022).

[10]. S. N. Zakiyyah, Irkham, Y. Einaga, N. S. Gultom, R. P. Fauzia, G. T. M. Kadja, S. Gaffar, M. Ozsoz, Y. W. Hartati, Green Synthesis of Ceria Nanoparticles from Cassava Tubers for Electrochemical Aptasensor Detection of SARS-CoV-2 on a Screen-Printed Carbon Electrode. *ACS Appl Bio Mater* **7**, 2488–2498 (2024).

[11]. A. Kurnia Sari, Yeni Wahyuni Hartati, Shabarni Gaffar, Isa Anshori, Darmawan Hidayat, Hesti Lina Wiraswati, The optimization of an electrochemical aptasensor to detect

RBD protein S SARS-CoV-2 as a biomarker of COVID-19 using screen-printed carbon electrode/AuNP. *Journal of Electrochemical Science and Engineering* **12**, 219–235 (2022).
